# A kinetic mathematical model of comprehensive iron metabolism in a respiring yeast cell: a basic-pathways approach to solving a large system dynamically

**DOI:** 10.1007/s10534-025-00758-7

**Published:** 2025-12-10

**Authors:** Paul A. Lindahl, Jay R. Walton

**Affiliations:** 1 Department of Chemistry, Texas A&M University, College Station, TX 77843-3255 USA; 2 Department of Biochemistry and Biophysics, Texas A&M University, College Station, TX 77843 USA; 3 Department of Mathematics, Texas A&M University, College Station, TX 77843-3368 USA

**Keywords:** Basic pathways, Biochemical reaction networks, Constraint-based modeling, Iron sulfur clusters, Hemes, Mutual autocatalysis, Null space, Sloppy models, Stoichiometric matrix, System’s biology

## Abstract

The individual functions of most iron-containing species in *Saccharomyces cerevisiae* are fairly-well understood, but less is known regarding how they function collectively as a unified system. Here, an ODE-based kinetic cell model was developed to reveal system’s-level behavior of iron metabolism. The dimensionally-accurate in silico cell was divided into 5 compartments. It contained 80 components that engaged in 169 reactions. The cell grew on nutrients IRON, CARBON and OXYGEN. All major iron-related processes were represented including the biosynthesis and metallation of iron-containing proteins, trafficking of labile iron pools, homeostatic regulation, respiration, the TCA cycle, iron-sulfur-cluster and heme biosynthesis, the synthesis of DNA, phospholipids, amino acids, and nucleotide triphosphates, and reactions involving oxygen and reactive-oxygen-species. Iron and carbon were conserved in reaction stoichiometries. The time-dependent model was solved using the Basic Pathways approach, despite limited kinetic information. Once regulated appropriately, the system could withstand perturbations in component concentrations by returning to its original steady-state. It responded to changes in nutrient iron and oxygen concentrations and to changes in rate-constants, yielding altered sets of steady-state component concentrations. The latter type of perturbation is tantamount to altering the expression level of a gene. This ability offers the potential to explain phenotypic changes of genetic mutations on the mechanistic molecular level. The model included all established iron-related cellular processes (albeit in combined forms), and a highly interrelated reaction network reflecting a mutually autocatalytic system. Steady-state iron concentrations in the cell, organelles, and components were reasonably near to those observed/estimated experimentally.

**Supplementary Information:**

The online version contains supplementary material available at 10.1007/s10534-025-00758-7.

## Introduction

Iron is an essential transition metal found in nearly all forms of life (Muckenthaler et al. 2017; Braymera et al. 2021; Ramos-Alonso et al. 2020). *Saccharomyces cerevisiae*, the simplest and arguably best understood eukaryotic cell, is known to contain approximately 117 iron-containing proteins (Table S1). These proteins are involved in a multitude of processes, including mitochondrial respiration (TCA cycle and electron transport chain), amino-acid, phospholipid, and protein biosynthesis, and DNA replication and repair (Lindahl 2019; Lindahl and Vali 2022). Upon entering the cell, nutrient iron becomes incorporated into a labile iron pool composed of non-proteinaceous low-mass iron coordination complexes. Iron from the pool is trafficked throughout the cell, assembled into iron-sulfur clusters, hemes, and other cofactors, and ultimately installed into recipient proteins and enzymes. The cell coordinates and regulates all of these activities within the background of carbon-based metabolism as the cell grows and divides. Iron-containing species in cells can also be dangerous, promoting the synthesis of reactive oxygen species (ROS) which can damage nucleic acids, proteins, and membranes. Such damaging processes, and the cell’s attempt to mitigate them, should also be included in any comprehensive descriptions of cellular iron metabolism.

A molecular-level understanding of how cells coordinate all of these aspects of iron metabolism is an unrealized grand challenge. Hindering this achievement is the enormous amount of required information, including the exact reactions involved, the rates of those reactions, and how those rates are affected/influenced by other cellular species and catalysts. Nevertheless, the reward for understanding cellular iron metabolism as a unified system is equally enormous, as it would provide a deeper understanding for treating iron specific diseases such as Friedreich’s Ataxia and hereditary hemochromatosis, and more general diseases such as cancer, Alzheimer’s and Parkinson’s (Jomova et al 2022).

In principle, such a goal could be achieved by developing ordinary-differential-equations (ODE) based kinetic models of comprehensive cellular iron metabolism (Saa and Nielsen 2017). Such systems specify how each iron-related cellular component is affected by other cellular components. Once integrated, such systems could predict the effects of mutating any gene/protein in the system, allowing, for example, the phenotype of a cell harboring a mutation in a disease-associated gene/protein to be understood on the molecular level.

What hinders the achievement of such models is insufficient kinetic-based information needed to solve such an ODE system *uniquely* (Jamshidi and Palsson 2008). This includes: a) the exact reactions involved in cellular iron metabolism; b) the rate-law expression controlling the rate of each reaction; c) rate-constants (*k*_*rxn*_) and Michaelis–Menten parameters (*K*_*m*_) needed for rate-law terms; and d) component concentrations [C_i_]. All such information must be assigned before any such ODE system could be integrated.

A common approach to minimizing this problem is to simplify reaction networks and reduce the number of required parameters. Resulting *course-grain* models can often be solved dynamically (time dependently), but they tend to be highly symbolic, overly simplistic, and difficult to test experimentally. At the other extreme are *GEnome-scale Models* (GEMs) that employ thousands of chemically-defined reactions and components (Fang and Palsson 2020; Lachance et al. 2021). Such models are comprehensive and more realistic, but they cannot be solved dynamically; rather, steady-state solutions are constrained using linear programming by optimizing an objective function (Edwards and Palsson 1999). Such *constraint-based* models can only probe the stoichiometric (***S***) matrix and its null space using flux-balance analysis.

Numerous mathematical models of iron metabolism have been developed. Alves and Sorribas (2007) developed an ODE-based model of mitochondrial iron-sulfur-cluster (ISC) biogenesis involving 41 reactions and 27 components. They used a power-law formalism for rate-law expressions. Amir et al. (2010) developed an ODE-based model that simulates observed dampened oscillations in *E. coli* gene expression levels following a sudden decline in nutrient iron levels. Their model included feedback-regulated cellular iron metabolism and a dilution term to account for exponential cell growth. Chifman et al. (2012) developed an ODE model of iron metabolism in breast epithelial cells, as well as a more complex model of iron homeostasis using a Boolean formalism and a discreet logic-based modeling framework that involved 24 components (Chifman et al 2017). Schirm and Scholz (2020) developed an ODE model of erythropoiesis which included 15 components solved at steady-state using parameters obtained by fitting against clinical data. Konstorum et al. (2020) developed a logic-based multistate coarse-grain model of ferroptosis, a type of programmed-cell-death that is iron-dependent and associated with cancer. Mendes and coworkers developed ODE models of iron sequestration kinetics of ferritin (Masison and Mendes 2023) and of iron physiology in mice suffering from iron-related diseases (Parmer and Mendes, 2019; Mitchell and Mendes 2013).

Constraint-based GEM models that highlight iron metabolism have also been developed. Dikicioglu and Oliver (2019) modified an existing GEM to include metabolic reactions involving hemes, ISCs and mononuclear iron centers. Malina et al. (2021) developed a GEM model of yeast mitochondria that included a multitude of iron-associated processes. The same group modified a GEM of whole yeast cells (Lu et al. 2019; Chen et al. 2021) by adding reactions involving iron trafficking and ISC assembly. Their model included protein synthesis and dilution due to cell growth. Simulations approximately reproduced cellular iron concentrations and predicted the percentage of cellular iron in various proteins as well as effects of iron deficiency.

The ODE-based models developed by Wofford and Lindahl (2015 and 2019), Fernandez et al. (2022), and Thorat et al (2023) represent the evolutionary lineage of the in silico cell developed here. These “ancestral” cells grew on nutrient IRON at a defined oxygenation level. Cell volumes were subdivided into cytosolic, mitochondrial, and vacuolar compartments. They typically contained about 10 or fewer components that participated in a similar number of reactions. The most advanced of these models can mimic the development of *Friedreich’s Ataxia* from a healthy state (Fernandez et al. 2022; Thorat et al 2023).

The Mössbauer-ironome described by Lindahl and Vali (2022) provides another foundation for the current in silico cell. In that study, Mössbauer spectra of whole yeast cells and organelles were simulated by summing contributions of most of the known iron-containing proteins, using concentrations estimated by quantitative proteomics (Ho et al 2018).

Our current goal was to develop an ODE-based in silico cell that approached the complexity of constraint-based GEM models in terms of iron metabolism, yet could be solved dynamically. Doing this was a challenge, due to the large size and complexity of the model and the limited relevant information available. All iron-containing proteins known when the study commenced were organized into 23 protein groups, ultimately affording a biochemical reaction network composed of 169 reactions and 80 components. Each iron-containing protein and non-proteinaceous iron-containing species was assigned a unique function, installed with appropriate iron centers, and housed in the appropriate cellular sub-compartment. Each component was chemically defined in terms of iron and carbon, and each reaction was balanced to adhere to the conservation of matter for those two elements. The focus was on iron, but a skeletal outline of common carbon-based metabolism was included as “housekeeping” reactions. The resulting in silico cell utilized realistic steady-state component concentrations, especially those involving iron, and it grew at the same rate as real cells. The system was solved dynamically and was robust enough to recover from modest perturbations in component concentrations or rate-constants. The dynamical cell system offers a nearly unlimited number of predictions of cellular phenotypes arising from such perturbations. We now describe it, starting from the assumed cell morphology.

### Cell morphology

Haploid fermenting Wild-Type (WT) cells have a median volume *V*_*cell*_ of 42 × 10^–15^ L (Jorgensen et al. 2002), and that was assumed for the in silico cell developed here. The same volume was assumed by Ho et al. (2018) in their proteomics analysis, and by Lindahl and Vali (2022) in their Mössbauer-ironome study. About 17% of cell volume is due to a peptidoglycan-based exterior wall, but this structure is largely devoid of iron in exponentially growing cells (Wofford et al. 2016) and so it was excluded here. On the other hand, respiring yeast cells are ~ 1.5 times larger than fermenting ones (Egner et al. 2002) so the two effects approximately cancel. The assumed *V*_*cell*_ can be viewed as the average steady-state volume of a population of respiring *S. cerevisiae spheroplasts*.

The in silico cell was subdivided into five compartments including cytosol, mitochondria, nucleus, vacuoles, and endoplasmic reticula. The fractional volumes for compartments *i* = *c, m, n, v, and e*, were defined as *f*_*i*_ = *V*_*i*_*/V*_*cell*_ such that $1 = f_{c} + f_{m} + f_{n} + f_{v} + f_{e}$. Fractional volumes for fermenting cells have been determined experimentally (Wei et al. 2012; Walters et al. 2018; Yamaguchi et al. 2011; Uchida et al. 2011). Under respiring conditions, mitochondria in yeast occupy ~ 10% of cell volume (Stevens 1977). Based on these results, the fractional volumes in Table S2 were assigned for the in silico respiring yeast spheroplast developed here.

### Phospholipids and membranes

Once fractional volumes were assigned, the volumes of the corresponding encapsulated phospholipid membranes could be estimated. The nucleus, vacuoles, and the entire cell were assumed to be spherical which allowed corresponding surface areas to be calculated. Resulting areas were multiplied by the thickness of a bilayer (0.006 µm) (Voet and Voet 2011a) to obtain membrane volumes. The nucleus was assumed to possess a double membrane (Voet and Voet 2011b). Mitochondria were assumed to consist of long, thin cylinders, with an inner membrane 3 × the surface area of the outer membrane. No shape was assumed for the ER; only that the membrane volume constituted *half* of the compartment’s total volume. The total volume of membranes in the cell was then taken as the sum of membrane volumes for each compartment, 3.6% of *V*_*cell*_ or 1.51 × 10^–15^ L.

The only cellular component with a defined shape was phospholipid **PL**. (Component names are introduced in bold.) PL had a near-cuboidal shape of volume 1 nm × 1 nm × 3 nm = 3 × 10^–24^ L; its long axis was presumed to be orthogonal to the membrane plane. This size and shape allowed the number of PL molecules per cell to be calculated from the total volume of cellular membranes (Table S2). That volume when divided by the volume of an individual PL molecule afforded 5.03 × 10^8^ PL molecules (or 8.36 × 10^–16^ mol) per cell. For a cell of volume *V*_*cell*_, the resulting cellular phospholipid concentration was nearly 20,000 µM. This and other component concentrations are given in Table S3. For simplicity, all cellular PL molecules were presumed to be in the ER (affording an unrealistic high *local* concentration of 752 mM). Cellular compartments were *not* subdivided into a surrounding membrane and aqueous region; all components in a compartment, including membrane-bound components, were assumed to be homogenously distributed within it.

### DNA

The *S. cerevisiae* genome is composed of 12,156,677 base pairs and 6275 genes; thus on average each gene contains 1937 basepairs (*Saccharomyces Genome Database*; https://www.yeastgenome.org/). Rather than viewing **DNA** as a single large molecule with a cellular concentration of 0.00003 µM, it was assumed to have a concentration of 6275 copies per cell (0.248 µM) each containing 1937 base-pairs (Table S3). In brief, the cell contained that many copies of a *generic* gene composed of a single nucleotide base (derived from component **NUCM**). This number of base-pairs implies that 3875 nucleotides were used for its synthesis. However, that number was doubled in our calculations to account for the additional nucleotides transiently required for both processes. Greater precession was unnecessary since DNA replication and transcription were only background or housekeeping processes in a model focused on iron metabolism. The carbon content of other cellular components was also approximated, as exact accounting was prohibitively complicated and unnecessary for the scope of this study.

### Protein groups

The ironome in *S. cerevisiae* has been used along with proteomic data (Ho et al. 2018) to simulate Mössbauer spectra of whole cells and each organelle (Lindahl and Vali 2022). However, constructing a kinetic model that included ~ 100 distinct iron-containing proteins would have been prohibitively large, so these proteins, along with other iron-free proteins involved in iron metabolism, were organized into 23 protein groups. Proteins were grouped (Table S4) according to function, cellular location, and according to whether deleting any member of the group would likely result in a common overall phenotype. Justification for organizing individual proteins into groups is provided in Appendix A.

Functional properties of individual member proteins have been described (Lindahl and Vali 2022) so here only the assumed properties of each protein group are summarized. Protein group **AFT** regulates the cell’s response to iron deficiency, including cellular iron import, the degradation of iron-rich components, and the trafficking of cytosolic iron into vacuoles and mitochondria. Group **ATM** catalyzes the export of an iron-sulfur cluster (ISC) from mitochondria to cytosol. **CAT** combats the effects of oxidative stress by catalyzing the degradation of ROS. **CCC** transports cytosolic iron to vacuoles. **CIA** uses the ISC exported by ATM to assemble Fe_4_S_4_ clusters and then install such clusters into various apo client proteins in cytosol/nuclei. **ETC** represents the mitochondrial Electron Transport Chain which catalyzes the reduction of O2 (indirectly) by NADH. **FT3** catalyzes the import of nutrient iron into the cytosol. **FT5** catalyzes the movement of vacuolar iron into the cytosol. **GRX** is a chaperone for ISC transfer reactions. **HEM** catalyzes heme formation and regulates heme trafficking and metabolism. **HMX** catalyzes heme degradation in the ER. **ISA** converts [Fe_2_S_2_] clusters to [Fe_4_S_4_] clusters in mitochondria and installed the latter into various apo client proteins. **ISU** assembles [Fe_2_S_2_] clusters in mitochondria and installs them into various apo client proteins. **LEU** catalyzes the synthesis of the amino acid AA in the cytosol. **LYS** catalyzes the synthesis of AA in mitochondria. **MEM** catalyzes the synthesis of PL in endoplasmic reticula. **MRS** catalyzes the import of cytosolic iron into mitochondria. **NUC** catalyzes the synthesis of the nucleotide used in the synthesis of DNA. **POL** catalyzes the synthesis of DNA in the nucleus. **PRO** includes all other proteins that are not otherwise involved in the model. Apart from consuming amino acids and ATP, this protein group has no influence on any reaction. **RIB** along with DNA catalyzes the synthesis of all protein groups. **TCA** represents the TriCarboxylic Acid cycle in mitochondria, which is involved in respiration. **YAP** catalyzes the synthesis of the vacuolar iron importer CCC, and the iron chaperone aGRX under high-iron conditions.

Each protein group was synthesized in an iron-free apo state. Protein groups that were active in that state were indicated by three capital letters (e.g. ATM). For groups that developed activity upon metallation, prefix “*a”* indicated the inactive apo form. For groups that were metallated in two steps, the double “aa” prefix designated the metal-free form (e.g. aaCIA) while the single “a” prefix designated the form housing only permanent iron centers (e.g. aCIA). No prefix was used for fully loaded versions which contained both permanent and transiently-bound iron centers (e.g. CIA). Apo forms were installed with some combination of 6 different iron cofactors including hemes, nonheme iron, [Fe–O–Fe], [Fe_2_S_2_], [Fe_3_S_4_], and [Fe_4_S_4_] clusters. These cofactors contained 1, 1, 2, 2, 3, and 4 irons, respectively, and were abbreviated *FH*, *FO*, *FF*, *F2*, *F3*, and *F4* also respectively.

For iron-containing protein groups not involved in trafficking or regulation (e.g. CAT), 90% of the protein molecules in the steady-state cell were assumed to be in the holo form and 10% in the apo form. For proteins involved in trafficking or regulation (e.g. AFT), holo and apo forms were each assumed to represent 50% of the total protein group concentration. For proteins with 3 forms (e.g. aaCIA, aCIA, and CIA), respective concentrations were assigned as 10%, 45% and 45% of the total protein group concentration.

The concentration of each protein group in a respiring cell was calculated as described in Table S4 and summarized in Table S3. For each member *i* of a group, reported *copies per cell* (Ho et al. 2018) were converted into µM cellular concentration (*[P*_*i*_*]*_*fer*_) as described (Lindahl and Vali 2022. Concentrations under respiration conditions (*[P*_*i*_*]*_*res*_) were obtained by multiplying mitochondrial proteins by 3 × or 9 × to account for an increased fractional volume of this organelle under respiring conditions and higher expression levels, respectively. The group concentration in a respiring cell *[P*_*g*_*]*_*res*_ was taken as the average of the member’s concentrations,

1 $$[{\mathrm{P}}_{{\mathrm{g}}} ]_{res} = \frac{1}{n}\sum\limits_{n} {[P_{i} ]_{res} }$$

*Local* concentrations, referring to those within a cellular compartment, were obtained by dividing *cellular* concentrations by the fractional volume of the compartment ([local] = [cellular]/*f*_*i*_). The ratio *[P*_*i*_*]*_*res*_/*[P*_*g*_*]*_*res*_ reflects the degree to which a member protein contributed to the group. This weighting factor was used to calculate the mass and carbon content of each group (see below).

### Non-proteinaceous iron components

The model contained 7 non-proteinaceous iron-containing species including labile iron pools in the cytosol (**FC**) and mitochondria (**FM**), Fe^III^ and Fe^II^ polyphosphate complexes in vacuoles (**F3** and **F2**), free heme centers (**HEME**), and mitochondrial and cytosolic nanoparticles (**MP** and **CP**). Each pool component was defined to contain 1 Fe per molecule. The cellular concentrations of these species were determined/estimated from previous Mössbauer spectra and from iron concentrations of whole cells and organelles (Lindahl and Vali 2022; SI of Thorat et al. 2023). For the healthy iron-replete wild-type conditions assumed here, the concentrations of MP and CP were presumed to equal zero.

### Housekeeping metabolites

Besides PL and DNA, the cell contained additional non-iron “housekeeping” metabolites. The same molecular component in different locations was counted as different components. One generic amino acid was included in the cell, called **AA** in the cytosol and **AAM** in mitochondria. Acetyl-CoA in the mitochondria (**ACA**) was **ACAC** in the cytosol. **ADP** and **ATP** in the cytosol were **ADPM** and **ATPM** in mitochondria. NAD(P)^+^ and NAD(P)H were **NAD** and **NAH**, respectively in cytosol, but **NADM**/**NAHM** and **NADV**/**NAHV** in mitochondria and vacuoles, respectively. The sole metabolite used to generate DNA was **NUCM** in the cytosol and **NUCMN** in the nucleus. Molecular oxygen in the cytosol, mitochondria, and vacuoles was **O2**, **O2M,** and **O2V**, respectively. Reactive oxygen species in the cytosol, ER, mitochondria, and nucleus were **ROS, ROSE**, **ROSM**, and **ROSN**, respectively. The sole metabolite used to drive the TCA cycle was **TCAM** in mitochondria and **TCAMC** in cytosol. Metabolite concentrations (Table S3) were estimated from reported values (Jewison et al. 2012; Cai et al. 2011; Boer et al. 2010; Park et al. 2016).

### Iron content of components

Although the names of many components imply a chemical species, their elemental compositions had to be explicitly defined before reaction stoichiometries could be assigned. The composition of each component was only defined in terms of iron and carbon (Tables S5–S7). Calculating this was challenging for protein groups since group members contained different numbers and types of iron centers. Moreover, the iron composition of protein groups was defined in terms of the 6 types of iron centers considered in the model. To do this, the iron content of member protein *i* was subdivided according to the type of center (Table S5), with the number of each type of center per protein, called *C*_*i,FH*_, *C*_*i,FO*_, *C*_*i,FF*_ etc. taken directly from (Lindahl and Vali 2022). These *molar iron coefficients* were multiplied by member weight to afford the number of iron centers per group protein contributed by that member. For example, the number of [Fe_2_S_2_] clusters per group contributed by member *i* would be

2 $$C_{g(i)F2} = C_{i,F2} \frac{{[P_{i} ]_{res} }}{{[{\mathrm{P}}_{{\mathrm{g}}} ]_{res} }}$$

Considering the first entry of Table S5, member protein Aft1 contained 0.5 [Fe_2_S_2_] clusters per monomer (one cluster per homodimer), and its weight within the AFT protein group was 1.54866. The number of such clusters in the group was then 0.5⋅1.54866 = 0.77433. The total number of [Fe_2_S_2_] clusters in a group was the sum of the individual molar coefficients for that type of center

3 $$C_{g,F2} = \sum\limits_{i} {C_{g(i),F2} }$$

In this example, member Aft2 contributed 0.07901 F2 clusters to AFT, whereas the other 2 members contributed nothing. The total number of F2 clusters per AFT monomer, symbolized *C*_*AFT,F2*_, was 0.77433 + 0.07901 + 0 + 0 = 0.85334.

The total number of irons per group (molar *iron* coefficient) was the sum of these molar iron-center coefficients multiplied by the number of irons per center, as in (4).

4 $$C_{g,Fe} = C_{g,FH} \cdot 1 + C_{g,FO} \cdot 1 + C_{g,FF} \cdot 2 + C_{g,F2} \cdot 2 + C_{g,F3} \cdot 3 + C_{g,F4} \cdot 4$$

Since AFT has no other Fe centers besides F2, *C*_*AFT,Fe*_ = 0.85334⋅2 = 1.70668.

### Carbon content of components

Although the model focused on iron, reaction stoichiometries also conserved carbon. Determining the exact carbon conservation would have been excessively difficult so approximate carbon contents were assigned for each component (Tables S7 and S8). For example, amino acid AA and acetyl-CoA were both defined to contain 5 carbons. ATP, ADP, NAD, NAH, NUCN, and TCAM each contained 10 carbons, and HEME contained 35 carbons. Such approximations were acceptable because carbon only served as a housekeeping element in the model.

For each protein group, the molar amino acid coefficient *C*_*aa,g*_ was calculated similar to the way that the iron content of such groups was calculated, namely by the equation

5 $$C_{aa,g} = \sum\limits_{i} {aa_{i} \frac{{[P_{i} ]_{res} }}{{[P_{g} ]_{res} }}}$$

where aa_*i*_ is the number of amino acids in member protein *i*. *C*_*aa,g*_ indicates the number of amino acids (AA) assumed to be used in the biosynthesis of the group. As an example, consider the first entry of Table S8 involving group AFT. Member protein Aft1 contains 690 amino acid residues (all aa data from Saccharomyces Genome Database). With a weighting factor of 1.54866, Aft1 contributed 690⋅1.54866 = 1223.35 amino acids to the group. Other members Aft2, Cth1, and Cth2 contributed 75.25, 388.05, 407.95, respectively, totaling 2197.75 amino acids in group AFT. Since each amino acid contained 5 carbons, the molar carbon coefficient for AFT was 10,988.75 (Table S7).

### Model reactions

**Fig. 1:**
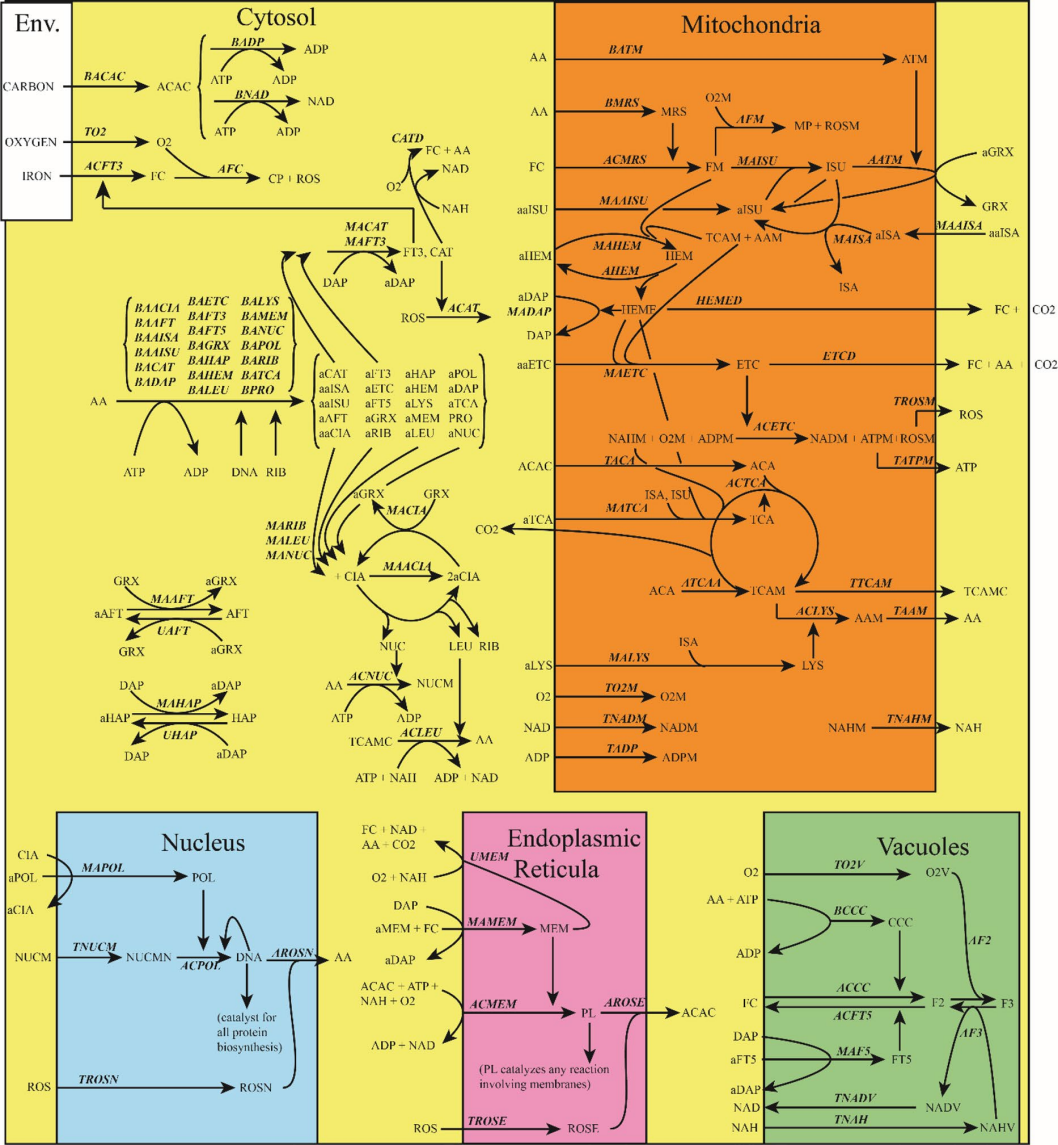
Overview of the biochemical reaction network assumed. See text for a general description and Tables S2 (cell morphology), S3 (component and their concentrations), and S9 (model reactions) for details

The 169 reactions occurring in the cell are listed in Table S9. An overview of the entire reaction network (Fig. 1) shows that compartments *c*, *m*, *n*, *v*, and *e* housed 43, 20, 5, 7, and 5 components, respectively. One hundred and twenty-seven reactions were *intracompartmental* which meant that they occurred entirely in one compartment (81, 27, 6, 9, and 4 reactions in the compartments listed respectively above). The remaining 42 *intercompartmental* reactions occurred at the interface of cytosol and another compartment. The other compartment was *m*, *n*, *v*, and *e* for 22, 5, 7, and 8 reactions, respectively. Sixteen intercompartmental reactions merely indicated the transfer of a component from one compartment to another.

Reactions (introduced in bold italics) were named according to a reaction category. ACtivities were prefaced with “AC”. Protein Biosynthesis reactions were prefaced with “B”, Metallation reactions with “M”, Transfer reactions with “T”, Unmetallation reactions with “U”, and Dilution reactions with “D”. The name of the reaction doubled for its rate. The following is a sampling of the reactions.

### Nutrient import

The cell grew exclusively on IRON, CARBON, and OXYGEN; these nutrients were converted into ***all*** cellular components according to the reaction network of Table S9. They were imported into the cell according to the reactions

***ACFT3***:{$IRON\mathop{\longrightarrow}\limits^{FT3}FC$}, ***BACAC:***{CARBON → 0.2 ACAC}, and ***TO2***:{OXYGEN → O2}. IRON enters the cell through the FT3 transporter which was a catalyst for the reaction. The coefficient 0.2 in reaction *BACAC* arose because CARBON was defined to contain 1 carbon atom and ACAC to contain 5. The conservation of mass demands that

6 $$a \cdot CARBON\left( {\frac{{\text{1 mol C}}}{{\text{1 mol CARBON}}}} \right) = b \cdot ACAC\left( {\frac{{\text{5 mol C}}}{{\text{1 mol ACAC}}}} \right)$$

where *a* and *b*, the stoichiometric coefficients of CARBON and ACAC, were assigned values of 1 and 0.2, respectively. Equivalent relationships were applied to each reaction to conserve iron and carbon. IRON and FC molecules were each defined to contain 1 iron, so the stochiometric coefficients for this reactant and product in reaction ACTF3 were 1.

### PL biosynthesis

Membrane component PL was synthesized from ACAC according to the MEM-catalyzed reaction ***ACMEM***: {10⋅ACAC + 28⋅ATP + 36⋅NAH + 4⋅O2 $\mathop{\longrightarrow}\limits^{MEM}$ PL + 28⋅ADP + 36⋅NAD} (see the ER compartment of Fig. 1). Given the molar carbon content of component (Table S7), the carbon balance for this reaction was {10⋅5 + 28⋅10 + 36⋅10 + 4⋅0 = 1⋅50 + 28⋅10 + 36⋅10}. PL was defined to contain 50 carbon atoms, similar to a real phospholipid composed of two C_20_ unsaturated fatty acid chains, a C_3_ glycerol core, and a C_7_ headgroup. *ACMEM* symbolized fatty acid biosynthesis, which begins with acetyl-CoA and involves reducing equivalents from NADPH, molecular oxygen, and free energy generated from ATP hydrolysis.

### Amino acid biosynthesis

Two protein groups catalyzed the biosynthesis of AA, namely LYS in mitochondria and LEU in cytosol. Both used TCAM as substrate. AA was synthesized in mitochondria by reaction ***ACLYS:*** {TCAM + ATPM + NAHM $\mathop{\longrightarrow}\limits^{LYS}$ 2⋅AAM + ADPM + NADM}. Product AAM (AA in mitochondria) was transferred to the cytosol for use in protein biosynthesis. AA was generated in the cytosol according to reaction ***ACLEU***: {TCAMC + ATP + NAH $\mathop{\longrightarrow}\limits^{LEU}$ 2⋅AA + ADP + NAD}. Although this AA did not require a subsequent transfer step, the TCA metabolite used as a substrate in the reaction had to be imported from mitochondria.

### Nucleotide biosynthesis

In real cells, nucleotides are synthesized primarily starting from amino acids. In the in silico cell, the same process was symbolized by reaction ***ACNUC*** {2⋅AA + 5⋅ATP $\mathop{\longrightarrow}\limits^{NUC}$ NUCM + 5⋅ADP} which was catalyzed by protein group NUC. This energy-consuming reaction consumes 2 amino acids and 5 ATP’s, and generates NUCM, the sole nucleotide metabolite in the cell.

### DNA biosynthesis

DNA catalyzed the synthesis of itself and all proteins. Protein group POL was a co-catalyst for the DNA replication reaction ***ACPOL***: {7750⋅NUCMN $\mathop{\longrightarrow}\limits^{DNA,\,POL}$ DNA} in which NUCMN was the substrate. The reaction occurred in the nucleus.

### Protein biosynthesis

All protein groups were biosynthesized in the cytosol. Using the CIA as an example, a typical reaction was ***BAACIA***: {4667.9⋅AA + 14,003.7⋅ATP $\mathop{\longrightarrow}\limits^{DNA,\,RIB}$ aaCIA + 14,003.7⋅ADP}. All such reactions were co-catalyzed by DNA and RIB (representing ribosomes). Membrane-bound proteins were additionally catalyzed by PL. In this capacity, the PL concentration was considered to be a reporter for the integrity of the membrane. The number of amino acids in each reaction corresponded to the number of residues in the protein group (Table S8, see *C*_*aa,g*_ for CIA). The installation of each AA into the growing chain was assumed to require the hydrolysis of 3 ATPs. One was used to activate each tRNA, and two were used to activate an elongation factor and to induce ribosome binding (Voet and Voet 2011c). Resulting biosynthesized group proteins were in their apo forms. In most protein biosynthesis reactions, the apo form was generated in the cytosol, and a subsequent metallation step was combined with a transport step to transport the holo protein into its final location.

### Iron trafficking reactions

The labile Fe^II^ pool in the cytosol (FC) had many fates. Under the healthy Fe-replete conditions considered here, some FC was trafficked into mitochondria and vacuoles via protein transporters MRS and CCC, respectively. PL was a co-catalyst for these processes since they involved membranes. Iron import into mitochondria was presumed to be catalyzed by a mitochondrial membrane potential. Membrane potential was not explicitly included in the model but was reflected by the concentration of ETC, and so this protein group was assumed to be a catalyst for the import reaction ***ACMRS***: {FC $\mathop{\longrightarrow}\limits^{MRS,ETC,PL}$ FM} where the product of the reaction represented the labile Fe^II^ pool in that organelle.

Import of FC into vacuoles, reaction *ACCC* (Activity of CCC), resulted in an Fe^II^ species (F2) which could be oxidized to Fe^III^ polyphosphate (F3) by molecular O2 (reaction ***AF2***). Vacuolar Fe^III^ could be re-reduced to Fe^II^ using NAH as the reductant (reaction ***AF3***). Vacuolar Fe^II^ could be exported back into the cytosol through transporter FT5, feeding into the cytosolic FC pool (reaction ***ACFT5***).

### Mitochondrial iron-sulfur cluster reactions

FM was used as feedstock for building [Fe_2_S_2_] clusters on the mitochondrial protein scaffold ISU. Once assembled, such clusters were transferred to apo forms of various client proteins. FM was installed into aISU according to reaction ***MAISU***: {aISU + 7.59143⋅FM → ISU}. Each aISU monomer contained 0.521673 permanent [Fe_4_S_4_] clusters and 0.475566 Fe^II^ centers with O/N ligands but no transient [Fe_2_S_2_] clusters (Tables S6). The fully metallated ISU monomer contained these same centers plus 3.795715 [Fe_2_S_2_] clusters. Those clusters were assembled in this reaction. Since each FM molecule contained 1 Fe, the indicated stoichiometric coefficient was required to build the [Fe_2_S_2_] clusters. The [Fe_2_S_2_] clusters on ISU were transferred in other reactions to various apo-proteins e.g. the mitochondrial respiratory complex aETC (see below).

Mitochondrial [Fe_4_S_4_] clusters were generated by the reductive coupling of two [Fe_2_S_2_] clusters, according to reaction ***MAISA:*** {aISA + 0.9416092⋅ISU + 1.787040⋅NAHM → ISA + 0.9416091⋅aISU + 1.787040⋅NADM}. Each aISA monomer contained 1.669610 [Fe_2_S_2_] clusters and no [Fe_4_S_4_] clusters whereas ISA contained the same number of [Fe_2_S_2_] cluster and 1.787040 [Fe_4_S_4_] clusters. These clusters were built using [Fe_2_S_2_] clusters from ISU. The following calculation was used to determine the stoichiometric coefficient on ISU.

7 $$\frac{1\,mol\;aISA}{1}\left( {\frac{1.787040\;mol\;F4}{{1\,mol\;aISA}}} \right) = \frac{b\,mol\;ISU}{1}\left( {\frac{3.795715\;mol\;F2}{{1\,mol\;ISU}}} \right)\left( {\frac{1\;mol\;F4}{{2\;mol\;F2}}} \right)\,b = 0.9416092$$

The coefficient on NAHM was defined in accordance with the requirements of the reductive-coupling reaction {$2[Fe_{2} S_{2} ]^{2 +} + 2e^{-} \mathop{\longrightarrow}\limits[Fe_{4} S_{4}]^{2 +}$} (Kunichika et al. 2021). Loaded ISA proteins transferred [Fe_4_S_4_] clusters to various client proteins e.g. aLYS, in reaction ***MALYS***: {aLYS + 0.3725193⋅ISA + 0.8127375⋅ISU + 0.249366⋅FM → LYS + 0.3725193⋅aISA + 0.8127375⋅aISU}. Each stoichiometric coefficient was determined as described in Table S9.

### Heme-related reactions

Porphyrin was synthesized using substrates TCAM and AAM, and reductant NAHM, reminiscent of the real process. Iron from the FM pool was installed into porphyrin to generate the HEME cofactor within the protein HEM, according to reaction ***MAHEM***: {aHEM + 7.054408⋅FM + 16.46029⋅TCAM + 16.46029⋅AAM + 7.054408⋅NAHM → HEM + 7.054408⋅NADM}.

Heme trafficking and regulation are poorly understood and so the corresponding reactions used in the model are less certain than most. Whether hemes move through membranes via protein transporters is uncertain; hemes are soluble in membranes so this does not seem to be required. Whether hemes migrate through cytosol and other aqueous regions of the cell alone or escorted by protein chaperones is also unknown. Free hemes tend to be toxic and highly reactive. Using genetically-encoded fluorescent probes, Hanna et al. (2016) detected 20–40 nM of free heme in the cytosol of yeast; concentrations in other cellular compartments were detectable but an order-of-magnitude lower. Dap1 is a heme-binding protein that may be involved in heme trafficking; however, it localizes to the ER and seems to function exclusively to install hemes into ER-proteins (Mallory et al. 2005). Heme regulation involves the Hap proteins (Hap1-5) but many molecular-level details remain uncertain. Heme binding to Hap1 stimulates expression of respiration-related (and often heme-containing) proteins and mitochondria themselves (Barba-Aliaga and Alepuz 2022). Heme degradation involves heme oxygenase (HMX) but many details are missing.

Our model assumed the simplest mechanism consistent with these observations. Accordingly, heme was synthesized in mitochondria by the ferrochelatase-containing protein group HEM, which then transferred the cofactor directly to the apo forms of all mitochondrial heme proteins. Other HEM molecules released free heme (HEME) into the cytosol via reaction ***UHEM***: {HEM → aHEM + 7.054408⋅HEME}. Free HEME diffused through the cytosol (at very low concentrations, to minimize toxicity) and was ultimately installed into all other heme-containing proteins in the cell.

### Respiration

The TCA cycle was represented by reaction ***ACTCA:*** {ACA + 3⋅NADM + ADPM $\mathop{\longrightarrow}\limits^{TCA,\,TCAM}$ 3⋅NAHM + ATPM + 5⋅CO2}. This housekeeping reaction was catalyzed by the TCA protein group and metabolite TCAM. TCAM symbolized all TCA cycle metabolic intermediates including citrate, isocitrate, α-ketoglutarate etc. In this reaction, the sole carbon-based substrate for the TCA cycle, acetyl-CoA (ACA), was oxidized to CO_2_, a waste product. Electrons from that oxidation generated NAH from NAD. In the same reaction, ATP was generated from ADP. In a related reaction (**ATCAA**) symbolizing anaplerotic reactions, ACA was the substrate used to replenish TCAM as it was depleted due to its use in the synthesis of the generic amino acid AA and nucleotide triphosphate NUCM.

The NAH generated by the TCA cycle became reoxidized to NAD by the electron transport chain (protein group ETC). The electrons thereby generated were used to reduce O2 to water in reaction ***ACETC***: {2⋅NAHM + O2M + 4⋅ADPM → $\mathop{\longrightarrow}\limits^{ETC,\,PL}$ 2⋅NADM + 4⋅ATPM + 0.05⋅ROSM}. This reaction was responsible for generating *all* cellular ATP. During actual respiration, protons are pumped across the mitochondrial inner membrane and that gradient is used to drive the synthesis of ATP from ADP. In the assumed reaction, ATP was generated directly; ETC and PL, whose concentrations reflected the integrity of cellular membranes, were catalysts. A side-product of respiration was reactive oxygen species ROS. The extent of ROS generation, assumed here to be 5% of the O2 that is reduced, is uncertain in real cells.

### Cytosolic ISC reactions

Some mitochondrial iron, commonly thought to be an [Fe_2_S_2_] cluster, is exported to the cytosol through the inner membrane transporter ATM for subsequent use by the CIA protein group in cytosolic ISC assembly. In the model, an [Fe_2_S_2_] cluster was transferred directly from ISU to the cytosolic glutaredoxin aGRX according to reaction ***AATM:*** {ISU + 1.538601⋅aGRX + 1.538601⋅ATPM $\mathop{\longrightarrow}\limits^{ATM}$ aISU + 1.538601⋅GRX + 1.538601⋅ADPM}. The reaction, also catalyzed by PL, involved the hydrolysis of ATP. Then the cluster was transferred from GRX to aCIA via reaction ***MACIA:***{aCIA + 3.816669⋅GRX + 4.7078421⋅NAH → CIA + 3.816669⋅aGRX + 4.7078421⋅NAD}. The transient cluster on CIA is an [Fe_4_S_4_] type. NAH provided the electrons required for the reductive coupling of two [Fe_2_S_2_] clusters. Then the cluster on CIA was transferred into various cytosolic apo- proteins, e.g. DNA polymerase aPOL, according to reaction ***MAPOL***: {aPOL + 1.6447644⋅CIA + 3.256706⋅FC → POL + 1.6447644⋅aCIA}. Other cytosolic apo-client proteins included those that catalyzed the synthesis of the amino acid (aLEU), the nucleotide (aNUC), and proteins (aRIB).

### Oxygen-related reactions

The in silico cell performed many reactions involving oxygen. Under healthy conditions, some cytosolic O2 moved into mitochondria (called O2M) and vacuoles (O2V). O2M was consumed by respiration and could, at sufficiently high concentrations, react with the mitochondrial Fe^II^ pool FM. O2V served to oxidized vacuolar F2 (Fe^II^) to F3 (Fe^III^). O2 was also used in the biosynthesis of PL.

The ROS generated in mitochondria as a side-product of respiration damaged the cell. Some cytosolic ROS was transported into the ER where it damaged membranes according to reaction **AROSE**. Some ROS moved to the nucleus where it damaged DNA according to reaction **AROSN**. Opposing this damage, heme-containing catalase CAT degraded cytosolic ROS via reaction **ACAT**.

### Regulation reactions

Real cells are homeostatically regulated to respond to iron deficiency and to minimize its effects, and in the model, the two well-known regulatory systems, AFT and YAP, were included. Under iron-replete conditions, the apo form of AFT (aAFT) reversibly accepts an [Fe_2_S_2_] cluster that was exported from mitochondria and bound to GRX. Under iron-deficient conditions, aAFT dominated. Balance was achieved by assuming two opposing reactions ***MAAFT*** {aAFT + 0.3459033⋅GRX → AFT + 0.3459033⋅aGRX} and ***UAFT*** {AFT + 0.3459033⋅aGRX → aAFT + 0.3459033⋅GRX}. In principle, under iron-deficient conditions, the rate of cluster export from the mitochondria would be slowed such that the GRX concentration would be low. Dominating aAFT would activate genes that encode the import of nutrient IRON and the export of vacuolar iron which stores iron under iron-replete conditions. These gene expression changes were designed to increase the flow of (scarce) nutrient IRON into the cytosol, decrease the flow of cytosolic iron into vacuoles for storage, and increase the flow of vacuolar iron into the cytosol. Numerous additional reactions were catalyzed by aAFT as these should be stimulated under iron-deficient conditions. One of these reactions recovered scarce iron by degrading iron-rich proteins including iron-rich MEM (***UMEM***). The other iron regulatory system involved YAP. Under iron-replete conditions, the holo YAP form should dominate. This group catalyzes the synthesis of CCC and aGRX; the former was used to import excess cytosolic iron into vacuoles for safe storage. The current model exclusively focused on the healthy Fe-replete respiring state, and so these regulatory mechanisms were not optimized. They were included to allow future studies of the Fe-deficient and Yfh1-deficient states.

### Cell growth and dilution reactions

Each simulation run assumed a population of cells in which growth and division were balanced such that their average volume and component concentrations were time-invariant. Growth of real cells in batch culture can be monitored by the optical density at 600 nm, in which case the exponential growth rate is given by the slope of ln(OD600) vs time, called α_cell_. The overall volume of a *population* of cells changes according to the equation $V_{t} = V_{0} e^{{\alpha_{cell} \cdot t}}$ but this was not simulated. Each simulation run was defined by: a) α_cell_; b) the average cell volume *V*_*cell*_; c) the concentration of each component in the cell, [C_i_]; and d) the concentrations of nutrients. α_cell_ was fixed at 0.003333 min^−1^, similar to that obtained for real wild type cells respiring on iron-sufficient aerobic minimal media (Moore et al 2018). Steady-state concentrations of components and nutrients are given in Table S3. IRON concentration for healthy Fe-replete conditions was 40 µM, as is typically used experimentally (using Fe^III^ citrate).

Cell growth was simulated by including a dilution term (with form -α_cell⋅_[C_i_]) in the ODE for each component *C*_*i*_. In words, the rate of dilution for each component equaled *α*_*cell*_ multiplied by the steady-state concentration of that component in its “home” compartment (called the ***local*** concentration). Dilution was treated as a chemical reaction even though the conservation of matter does not hold; reactants simply disappeared, e.g. reaction ***DAA*** (AA → …). There were 80 such reactions, one for each component.

### Solving the steady-state system

**Fig. 2:**
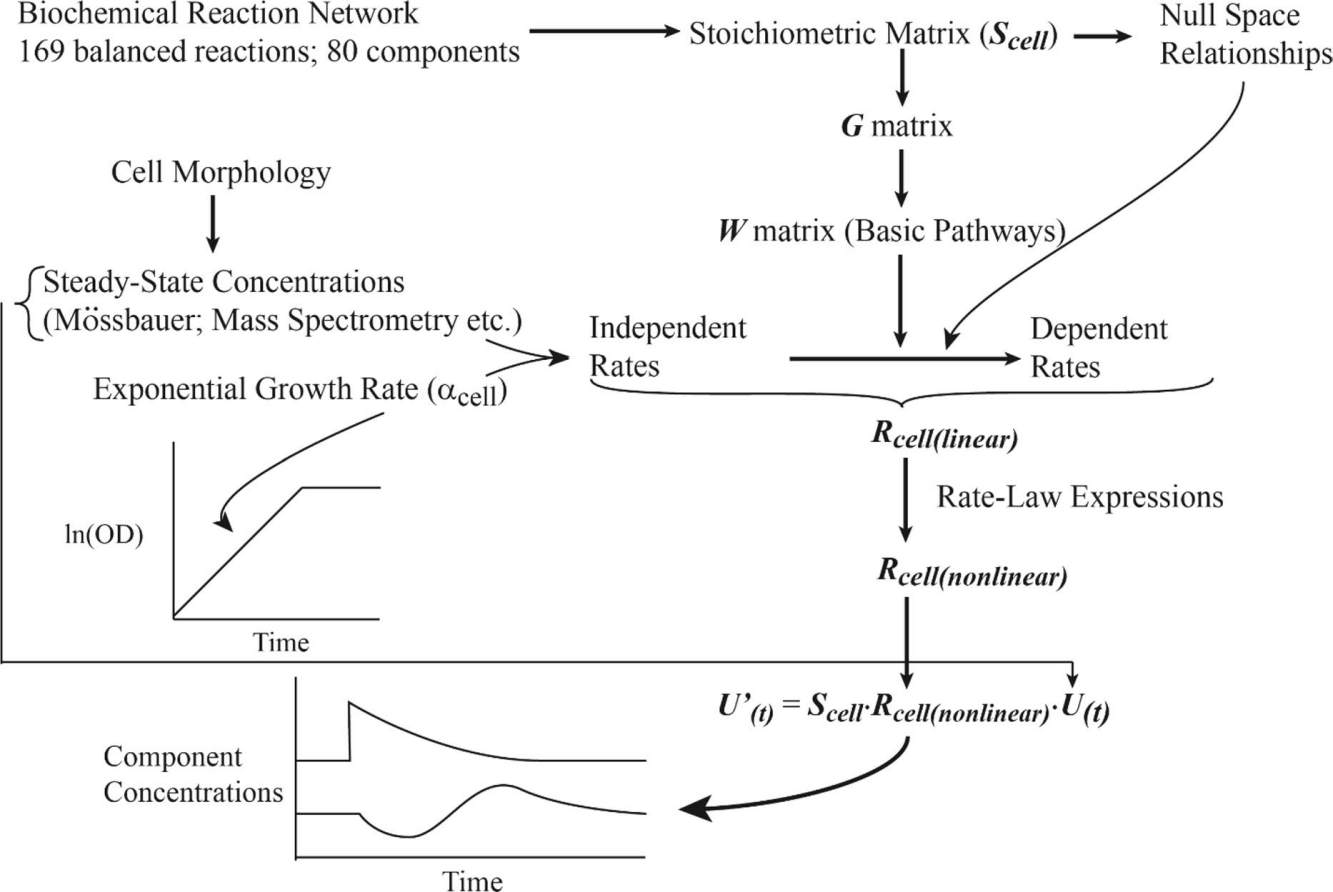
Overview of the approach used

With the cell morphology, model components, and biochemical reaction network of the model described, the next step was to develop the ODE system and solve it under an expanding steady state condition in which the growth rate is balanced by the dilution rate. An overview of our approach is given in Fig. 2.

### Reaction rates and the stoichiometric matrix

The stoichiometric matrix associated with the reaction network, ***S***_***cell***_, is given in the separate SI document called *Smatrix*. To understand how this matrix was constructed, consider the generic irreversible *cytosolic* reaction (8)

8 $$aA + bB \to cC + dD \ldots + X$$

in which *a*–*d* are stoichiometric coefficients for reactants and products *A*–*D*. *X* symbolizes the “extent of the reaction”, an imaginary product of the reaction always with stoichiometric coefficient 1. The rate of the reaction is defined as the incremental time-dependent change in the extent of reaction, namely *R*_*rxn*_ = d[X]/dt, where [X] equals *n*_*X*_/*V*_*cyt*_. Parameter *n*_*X*_ is the moles of *X* generated by the reaction and *V*_*cyt*_ is the cytosolic volume. The rate of reaction is related to the rate of change of each component of the reaction according to (9)

9 $$R_{rxn} = \frac{d[X]}{{dt}}\; = - \frac{1}{a}\frac{d[A]}{{dt}}\; = - \frac{1}{b}\frac{d[B]}{{dt}}\; = \frac{1}{c}\frac{d[C]}{{dt}}\; = \frac{1}{d}\frac{d[D]}{{dt}}$$

which can be rearranged into matrix form (10).

10 $$\left[ {\begin{array}{*{20}c} { - a} \\ { - b} \\ c \\ d \\ \end{array} } \right]\left[ {R_{rxn} } \right]\; = \left[ {\begin{array}{*{20}c} {\frac{d[A]}{{dt}}} \\ {\frac{d[B]}{{dt}}} \\ {\frac{d[C]}{{dt}}} \\ {\frac{d[D]}{{dt}}} \\ \end{array} } \right]$$

The matrix on the far left is the ***S*** matrix, with rows and columns equal to the number of components and reactions, respectively.

The matrix elements of ***S*** are affected by intercompartmental reactions and multiple cellular compartments in the model. For intercompartmental reactions, some components will be in the cytosol and others in a different cellular compartment, rendering ambiguous how reaction rates should be defined. Since rates of both intracompartmental and intercompartmental reactions are included in the null-space relationships of ***S***_***cell***_, all rates must be defined consistently. We define the rate of each reaction as it would occur in the cytosol, *even when it occurs in a different compartment or at the interface of cytosol and a different compartment*. Defining the reaction rate as occurring in the cytosol is tantamount to imagining that *X* for each reaction is in the cytosol *even if the actual components of the reaction are located elsewhere*. The rates of the same reaction as it occurs (or would occur) in another compartment *i* can be obtained by multiplying the cytosolic rate by the ratio *V*_*cyt*_*/V*_*i*_.

The concentration-change of a component (left-hand side of the ODE’s) due to a reaction refers to the *local* concentration of the component. If *A*, *B*, *C*, and *D* of (8) are located in the cytosol, mitochondria, nucleus, and vacuoles, respectively, the conservation of matter requires that

11 $$\frac{{dn_{X} }}{dt}\; = - \frac{1}{a}\frac{{dn_{A} }}{dt}\; = - \frac{1}{b}\frac{{dn_{B} }}{dt}\; = \frac{1}{c}\frac{{dn_{C} }}{dt}\; = \frac{1}{d}\frac{{dn_{D} }}{dt}$$

Multiplying each term by the volume ratio *V*_*i*_*/V*_*i*_, where *i* indicates the compartment in which component *i* is located, yields

12 $$\frac{{V_{cyt} }}{{V_{cyt} }}\frac{{dn_{X} }}{dt}\; = - \frac{1}{a}\frac{{V_{cyt} }}{{V_{cyt} }}\frac{{dn_{A} }}{dt}\; = - \frac{1}{b}\frac{{V_{mit} }}{{V_{mit} }}\frac{{dn_{B} }}{dt}\; = \frac{1}{c}\frac{{V_{nuc} }}{{V_{nuc} }}\frac{{dn_{C} }}{dt}\; = \frac{1}{d}\frac{{V_{vac} }}{{V_{vac} }}\frac{{dn_{D} }}{dt}$$

Converting into local concentrations and dividing both sides by *V*_*cyt*_ allows us to define the rate of the reaction

13 $$R_{rxn(cyt)} = \frac{{d[X]_{cyt} }}{dt}\; = - \frac{1}{a}\frac{{d[A]_{cyt} }}{dt}\; = - \frac{1}{b}\frac{{V_{mit} }}{{V_{cyt} }}\frac{{d[B]_{mit} }}{dt}\; = \frac{1}{c}\frac{{V_{nuc} }}{{V_{cyt} }}\frac{{d[C]_{nuc} }}{dt}\; = \frac{1}{d}\frac{{V_{vac} }}{{V_{cyt} }}\frac{{d[D]_{vac} }}{dt}$$

Rearranging (13) into matrix form yields

14 $$\left[ {\begin{array}{*{20}c} { - a} \\ { - b\frac{{V_{cyt} }}{{V_{mit} }}} \\ {c\frac{{V_{cyt} }}{{V_{muc} }}} \\ {d\frac{{V_{cyt} }}{{V_{vac} }}} \\ \end{array} } \right]\left[ {R_{rxn} } \right]\; = \left[ {\begin{array}{*{20}c} {\frac{{d[A]_{cyt} }}{dt}} \\ {\frac{{d[B]_{mit} }}{dt}} \\ {\frac{{d[C]_{nuc} }}{dt}} \\ {\frac{{d[D]_{vac} }}{dt}} \\ \end{array} } \right]$$

Thus, any ***S***_***cell***_ matrix elements associated with non-cytosolic components are the product of the stoichiometric coefficient for that reaction and the volume ratio *V*_*cyt*_*/V*_*i*_.

A similar situation holds for the rates of dilution reactions since they are also included in the null-space relationships. The dilution rate will depend on the local concentration of the component undergoing dilution. Once again, all dilution rates refer to those that would be obtained if the component were in the cytosol, even if it is located elsewhere. This is obtained by multiplying the non-cytosol component concentration by the volume ratio *V*_*cyt*_/*V*_*i*_*.*

### Basic pathways associated with the reaction network

We next constructed a basis for the null space of ***S***_***cell***_ that consisted entirely of chemically meaningful reaction rate vectors each of which represents a “Basic Pathway” (BP) within the reaction network (Walton and Lindahl 2023). We then used those BPs along with null space relationships and an assigned set of independent rates to obtain a complete set of steady-state reaction rates, one for each of the 169 reactions in the network (Table S10). To understand this process, let ***R***_***cell***_ be an *n-*vector (in this case *n* = 169) of steady-state reaction rates that lies in the null space of ***S***_***cell***_, that is ***S***_***cell***_***⋅R***_***cell***_ = 0 (Schilling et al. 2000; Palsson 2006). To be chemically meaningful, ***R***_***cell***_ will consist exclusively of nonnegative entries, but that is not guaranteed simply by being in the null space. To achieve this additional condition, a special feature of ***S***_***cell***_ was exploited arising from the assumed exponential growth of the cell. Such stoichiometric matrices have the structure

15 $$S_{cell} = (S_{0} | - {\mathbf{D}}_{m} )$$

where ***D***_*m*_ denotes an *m* × *m* diagonal matrix and ***S***_***0***_ denotes the *m* × *r* stoichiometric sub-matrix of ***S***_***cell***_. The -***D***_***m***_ submatrix arises from the assumption that the cell is part of an exponentially growing culture. The ODE for each component *C*_*i*_ includes a term *–α*_*cell*_*⋅[C*_*i*_*]*. This term is effectively a rate-law expression for dilution “pseudo-reactions” of the form {C_i_ →}. Dilutions were treated as reactions in the ODE for each C_i_ with a stochiometric coefficient of -1. All dilution terms were organized on the right-side of the ***S***_***cell***_ matrix to create the *-D*_***m***_ submatrix.

We previously showed (Walton and Lindahl 2023) that ***S***_***cell***_ matrices such as in (15) are full rank (rank *m*) and consequently have an *r-*dimensional null-space of the *n-*dimensional Euclidean space of reaction pathway vectors with an algebraic basis consisting of the column vectors of the matrix

16 $${\boldsymbol{G}}=\left(\begin{array}{c}{I}_{r}\\ \dots \\ {D}^{-1}{S}_{0}\end{array}\right)=({\boldsymbol{g}}1,\dots ,{\boldsymbol{g}}r)$$

For our system, ***S***_***cell***_ has 169 columns and 80 rows.

These null space basis vectors ***g***_***1***_…***g***_***r***_ may not be chemically feasible if they contain negative entries. Through a process similar to Gaussian elimination called *Singleton Theory* (Walton and Lindahl 2023), the ***G*** matrix can be transformed into the ***W*** matrix

17 $$W \, = \, \left( {w_{1} , \, \ldots \, ,w_{r} } \right)$$

whose column vectors ***w***_***1***_…***w***_***r***_, also called *Basic Pathways BP*_*1*_…*BP*_*r*_, are all nonnegative. The ***W*** matrix used here is given as an SI document called *Wmatrix*. These column vectors form an equivalent basis for the null space of ***S***_***cell***_ consisting of chemically feasible reaction pathways*.* Because of this, every steady-state reaction-rate vector ***R***_***cell***_ = *(R*_*j*_*)*, *j* = *1,…,169,* has a unique representation, as shown in (18),

18 $$R_{cell} = {\mathrm{c}}_{{1}} w_{1} + {\mathrm{c}}_{{2}} w_{2} ...{\mathrm{c}}_{{\mathrm{r}}} w_{r} \quad \left[ {\begin{array}{*{20}c} {{\mathrm{R}}_{{1}} } \\ \vdots \\ {R_{cell} } \\ \vdots \\ {{\mathrm{R}}_{{{169}}} } \\ \end{array} } \right]\; = {\mathrm{c}}_{{1}} \left[ {\begin{array}{*{20}c} {} \\ \vdots \\ {BP01} \\ \vdots \\ {} \\ \end{array} } \right]\; + {\mathrm{c}}_{{2}} \left[ {\begin{array}{*{20}c} {} \\ \vdots \\ {BP02} \\ \vdots \\ {} \\ \end{array} } \right]\; + ...{\mathrm{c}}_{{{89}}} \left[ {\begin{array}{*{20}c} {} \\ \vdots \\ {BP89} \\ \vdots \\ {} \\ \end{array} } \right]\;$$

where *c*_*1*_…*c*_*r*_ are coefficient weights of the independent Basic Pathway vectors. The right-hand-side expansion of equation (18) highlights the vector nature of BPs, with matrix elements that are often (but not always) 0 or 1. ***R***_***cell***_ will be chemically feasible if all *c*_*1*_*,…,c*_*r*_ are nonnegative. Under certain conditions, linear combinations (18) can produce chemically feasible pathways ***R***_***cell***_ with some weights *c*_*j*_ being negative. With the aid of Singleton Theory, necessary and sufficient conditions on the weights *c*_*1*_*,…,c*_*r*_ that guarantee when ***R***_***cell***_ is a chemically feasible pathway are derived and proved in Walton and Lindahl (2023).

In general, ***S***_***cell***_***⋅R***_***cell***_ = 0 is an underdetermined algebraic system whose solution set is an *(n-m)-*dimensional subspace of *n-*dimensional Euclidean space with *n* > *m*. As discussed above, every stoichiometric null space vector ***R***_***cell***_ has a unique representation (18) which can be rewritten using the ***W*** matrix as

19 $$R_{cell} = \, W \times C_{BP}$$

where ***C***_***BP***_ denotes the *r*-dimensional vector with components *c*_*1*_*,…,c*_*r*_. Linear system (19) is in general overdetermined, necessitating *m* compatibility constraints on the rates of ***R***_***cell***_ in order to solve for ***C***_***BP***_. As discussed below, the singleton structure of the ***W*** matrix was exploited to solve (19) for the vector ***C***_***BP***_ and to determine analytical expressions for the associated compatibility constraints on ***R***_***cell***_, as listed in Appendix B.

### Determining steady-state rates

Relations (18) and (19) express how ***R***_***cell***_ is related to the Basic Pathways basis *{w*_*1*_*,…,w*_*r*_*}*, and these were used to determine the steady-state reaction rates in ***R***_***cell***_ (Table S10). Specifically, relations were derived giving the weight coefficients *{c*_*1*_*,…,c*_*r*_*}* as functions of *r* specific components *{R*_*k(1)*_*,…,R*_*k(r)*_*}* of ***R***_***cell***_. These components of ***R***_***cell***_ are the 89 *independent* rates. The derivation made extensive use of certain structural properties of the ***W*** matrix. Firstly, ***W*** had high sparsity, defined as the proportion of zero entries. The sparsity of the ***W*** matrix used here was 0.89. Secondly, every row and column of ***W*** contains at least 1 nonzero entry. A row of ***W*** is called a *Singleton Row* if it contains exactly 1 nonzero entry. If a Singleton Row *i* has its sole nonzero entry in the *j*^*th*^ column of ***W***, it follows from (18) that weight *c*_*j*_ satisfies the relation

20 $$c_{j} = R_{i} /w_{ij}$$

where *w*_*ij*_ denotes the *ij*^*th*^ component of ***W*** and *R*_*i*_ denotes the *i*-th rate of ***R***_***cell***_. The *Singleton Row Rank* of ***W*** is defined as the proportion of its rows that are Singleton Rows; ***W*** has 88 Singleton Rows so its rank is 88/169 = 0.52. Basic Pathway *j* (***w***_***j***_, the *j*^*th*^ column of ***W***) is said to have a Singleton Element *w*_*ij*_ if row *i* is a Singleton Row with *w*_*ij*_ being its only nonzero element. ***W*** would be *Singleton Complete* if all of its columns have at least one Singleton Element, otherwise it is *Singleton Deficient* with a *Deficiency Index* equal to the number of its Singleton Deficient columns. Of the 89 columns in the ***W*** matrix, 15 were Singleton Deficient. Thus, in representation (20), 74 of the 89 *c*_*j*_ weights could be solved-for if *R*_*i*_ in (20) were chosen to be one of the 89 independent rates selected from the 169 rates in ***R***_***cell***_, and so these were so chosen. The remaining 15 weights in (18) were found by solving 15 linear equations that, because of the high sparsity of ***W***, had simple structures. Seventy-six of the 80 dilution reactions were selected to be independent because their rates could be calculated from the growth rate of the cell, which was easily measured experimentally, multiplied by the steady-state concentration of the component, which could be either measured or reasonably estimated. The four dilution reactions chosen as dependent were DCO2, DNAD, DROS, and DROSM. The required set of 89 independent reactions was completed by including the 13 non-dilution reactions {ACAT, ACFT5, AROSE, AROSN, BNAD, CATD, ETCD, TAAM, TNADV, TROSM, UAFT, UHMX, UMEM}with 76 dilution reactions. Of these non-dilution reactions, 7 could be justifiably set to zero including ACFT5, CATD, ETCD, TNADV, UAFT, UHMX, and UMEM (justified because the reactions will be inactive under healthy Fe-replete conditions). This left ACAT, AROSE, AROSN, BNAD, TAAM, and TROSM unassigned. We assumed that the two transfer reactions and the degradation of ROS by CAT were relatively fast, the degradation of PL by ROS and the synthesis of NAD were intermediate, and the degradation of DNA by ROS was slow (Table S9).

Once all independent rates were assigned, then ***C***_***BP***_ was known and the remaining unknown components of ***R***_***cell***_, representing *dependent* rates, could be calculated from (19). This strategy, detailed in Appendix C of the Supplemental Information, was used to calculate a complete set of steady-state reaction rates for the model (Table S10).

Viewed informally, *BPs form a basis for the stoichiometric null-space that consists of chemically feasible reaction pathways.* Every chemically feasible steady-state reaction pathway can be expressed as a unique linear combination of BPs. BPs often describe a *stoichiometric flow* from nutrients to *destination* components. Unlike most flows, stoichiometric flow is not time-dependent. Rather, it describes how molecular species work together autonomously in a reaction sequence or pathway. Each BP reveals the exact combination of reactions, based on the stoichiometry of the reactions, that are involved in converting nutrients into specific destination components. A tell-tale sign of a destination component is that it is *diluted* in the BP (since it’s made in excess). Intermediates in the process might also be generated in excess, in which case they are also diluted. In rare cases, a BP may neither consume nutrients nor generate destination components; for example, it may represent a cyclical process.

**Fig. 3:**
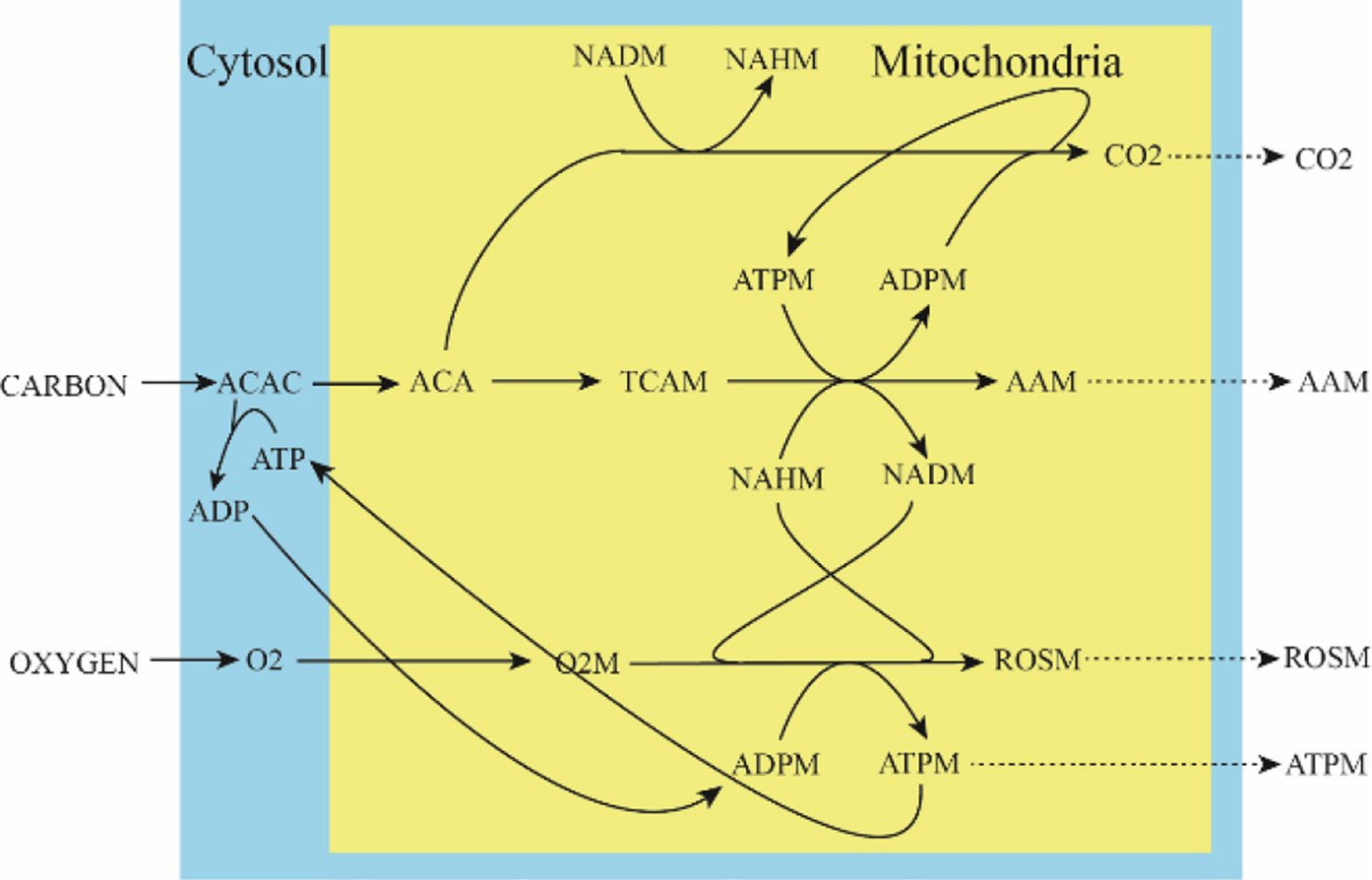
Illustration of a Basic Pathway (BP08). See text for a description

All 89 BPs in the ***W*** matrix are given in a separate SI document called *BasicPathways*. These pathways are illustrated in Fig. 3 using BP08 which describes the flow of nutrient CARBON to the destination component AAM, the amino acid generated in mitochondria. To generate AAM, CARBON is converted into acetyl-CoA in the cytosol (ACAC) which is transferred into mitochondria and converted to the TCA intermediate TCAM. TCAM is converted to AAM which is then diluted, as expected for a destination component. This process requires energy in the form of ATP, which in turn requires nutrient OXYGEN and NADH in mitochondria (called O2M and NAHM, respectively) to react. The latter species was generated by burning acetyl-CoA into CO2 using the TCA cycle in mitochondria. The NADH thus generated was oxidized by O2 via the electron transport chain. This generated the ATP needed for the synthesis of AAM. ROSM was generated as a biproduct, and so it must also be diluted.

### Advantages offered by the basic pathways (BP) approach

The BP approach assures that all reaction rates are positive. It must be emphasized that requiring reaction rates to be positive is a modeling choice. The main issue here is whether or not reaction reversibility is considered. For the sake of simplicity, we chose to ignore reaction reversibility. There are multiple modeling mechanisms for treating reversible reactions. One popular approach to modeling reversible reactions is to model the reverse direction by simply reversing the signum of the rate-law expression used for the forward reaction. Another approach is to include both the forward reaction and the reverse reaction as separate reactions in the network where the rate-law expressions for forward and reverse reactions need not differ simply via a signum change. There are a number of reasons why the first option is ill-advised. For example, if the model contains just the single reaction with reversibility consisting of a signum change, then the flux cone characterizing chemically feasible rate vectors is not pointed and hence not confined to the nonnegative orthant of the stoichiometric null-space. This greatly complicates devising a complete characterization of the full set of chemically feasible rate vectors. The second approach to modelling reversible reactions leads to enlargement of the stoichiometric matrix and dimensionality of the flux cone, but the cone is pointed which, as discussed in Walton and Lindahl (2023), simplifies the problem of calculating all chemically feasible steady-state reaction rate vectors. We chose to not address these complications in this paper.

The BP approach readily allows the full reaction network to be decomposed into subnetworks called basic pathways each of which can be evaluated separately for stability. We relied on this aspect to help diagnose which reactions led to the instabilities that we observed (see below), and thus allowed us to correct those problems through regulation of relevant biosynthesis reactions. The basic pathways approach greatly facilitated constructing a chemically feasible steady-state, identified as the foundational wild-type rate vector *wt*, which was exploited to calculate a wild-type set of component steady-state concentrations used in defining the rate-law expressions needed for the dynamic ODE simulations. Moreover, the W-matrix whose columns are the basic pathways is sparse, so that each basic pathway, corresponding to a small subnetwork, can readily be interpreted chemically. The rate-law expressions used to define the ODE system governing the network dynamics of our model makes use of the *wt* state in such a way that the corresponding *wt* component concentration vector ***U***_*ss*_ is a locally, asymptotically stable steady-state of the nonlinear ODE system. The basic pathways theory and application facilitated this whole approach to modeling network dynamics in a way that readily generalizes to genome-sized networks. Additionally, the BP approach is more straightforward and convenient to implement than any of the related methods such as extreme pathways, elementary modes, minspan, etc. All of the techniques used to implement the BP theory are standard tools from linear algebra as explained and illustrated in Walton and Lindahl (2023).

Another approach to studying the steady-state structure of a reaction network is flux balance analysis (FBA). In our opinion, FBA is more cumbersome and inefficient for the task at hand than the BP approach. For constructing the *wt* steady-state reaction flux vector for our 80 × 169 dimensional stoichiometric matrix making use of known values for steady-state component concentrations, we make critical use of the BP theory and algorithms developed in Walton and Lindahl (2023). The BPs form a basis for the stoichiometric null space and they are elements of the flux cone in the positive orthant of the stoichiometric null space consisting of all chemically feasible steady-state pathways. The construction of the BP matrix ***W*** uses only elementary methods from linear algebra and scales linearly with system size. In contrast, the FBA method relies on solving linear programming problems whose computational complexity scales in polynomial time and would be challenging to solve for large, genome scale reaction networks. Moreover, since FBA uses linear programming, the feasibility region for solving such problems must be a compact, convex polytope, whereas the BP approach gives a complete characterization of the full, infinite feasibility cone for the metabolic network.

### Solving the dynamical system

With all steady-state rates in ***R***_***cell***_ assigned, the next step was to solve the system under time-dependent *dynamical* conditions. Doing so required that a rate-law expression be assigned to each reaction in ***R***_***cell***_. Since these expressions had not been determined experimentally, we assumed simple expressions based on the following rules. Mass-action kinetics were assumed for the few reactions that were presumed not to be enzyme-catalyzed. In these cases, substrate concentrations were assumed to be first-order and were normalized to the concentrations assigned to them for steady-state healthy, iron-replete wild-type conditions, abbreviated [C_i_]_*w*_. Reactions presumed to be enzyme-catalyzed employed a Michaelis–Menten-like term for each substrate. In the event of multiple substrates, each term was multiplied together as in the first equation of (21) which would be the rate-law expression for the generic two-substrate reaction in (8).

21 $$\begin{gathered} R_{rxn(t)} = \frac{{k_{rxn} \cdot [A]_{(t)} }}{{K_{MA} + [A]_{(t)} }} \cdot \frac{{[B]_{(t)} }}{{K_{MB} + [B]_{(t)} }} \cdot \frac{{[ENZ]_{(t)} }}{{[ENZ]_{w} }} \hfill \\ R_{rxn(ss)} = k_{rxn} \frac{1}{2} \cdot \frac{1}{2} \cdot 1 \hfill \\ \end{gathered}$$

Time-dependent parameters are indicated by subscript (*t*). In this example, the reaction is catalyzed by enzyme ENZ. All catalytic influences were presumed to be first-order and normalized to the local steady-state WT concentration of the enzyme (Table S3). *K*_*M*_ values were uniformly equated to the WT concentration of the associated substrate. When the concentrations of both substrates A and B and enzyme ENZ are those for WT conditions, the expression simplifies to the second equation of (21). This allowed rate-constant *k*_*rxn*_ to be determined from the corresponding rate in ***R***_***cell***_ (Table S10).

Rates of transport reactions (when a component moves from one compartment to another) were defined to be proportional to the ratio of concentrations of the component in the “donating” compartment divided by that in the “receiving” compartment. For example, O2 was transported from cytosol to mitochondria for use in respiration which occurs in that organelle. In this case, the rate-law expression for reaction TO2 was assigned

22 $$TO2M = k_{TO2M} \left( {\frac{{[O2]_{(t)} }}{{[O2M]_{(t)} }} - 1} \right)$$

Such expressions significantly improved the stability of the dynamical system to perturbations. Assumed rate-law expressions for each reaction are given in Table S11.

The dynamic state was represented by a nonlinear system of ODEs. The state variables were defined to be the 80-dimensional vector ***U(t)*** consisting of component concentrations. The dynamic state corresponded to the solution to the system of ODEs

23 $$U_{(t)}^{'} = S_{cell} \cdot R(U_{(t)} )$$

where ***U***^***′***^_*(t)*_ denotes time differentiation and ***R(U***_*(t)*_*)* denotes the 169-dimensional rate vector containing the rate-law expressions, each a function of state variable ***U***_*(t)*_*.* Solving this ODE system starting at *t* = 0 required that an initial state ***U***_*(0)*_ (Table S3) be used. The resulting initial-value problem was then be solved numerically since all kinetic parameters in ***R(U***_***(t)***_*)* had been assigned. The symbolic software package *Mathematica* (https://www.wolfram.com/mathematica/) was chosen as the computational environment for all symbolic and numerical calculations.

We first verified theoretically that assigning the initial state to be the steady-state concentration vector ***U***_***ss(wt)***_, that is ***U***_***(0)***_ = ***U***_***ss(wt)***_, resulted in ***U***_***(t)***_ = ***U***_***ss(wt)***_ for all time. We then explored the domain of attraction of the steady-state solution ***U***_***ss(wt)***_. Unfortunately, ***U***_***ss(wt)***_ was unstable to any perturbation; when the right-hand-side of (23) equaled zero at t = 0, when ***U***_***(0)***_ = ***U***_***ss(wt)***_, any perturbation in the initial state ***U***_***(0)***_ resulted in chaotic dynamics. We conjectured that this behavior might be due to destabilizing feedback loops, perhaps associated with our efforts to make the entire system mutually autocatalytic. The lack of local asymptotic stability of the ***U***_***ss(wt)***_ state manifested in a somewhat peculiar fashion. Taking ***U***_***ss(wt)***_ as the initial state for the dynamical system (23), the computed solution ***U***_***(t)***_ remained equal to ***U***_***ss(wt)***_ for several hundreds or thousands of minutes before round-off error was sufficiently large to cause the dynamics to depart from that state. Examining which components were most responsible for the instability led to the recognition that various biosynthesis reactions needed to be regulated to prevent mutual autocatalytic blow-up. The instability appeared to be associated with a dramatic increase in the dimensionless normalized concentration [FM]_(t)_/[FM]_ss(wt)_. This led us to inspect the ODE governing the evolution of [FM](t) (namely [FM]´(t) = 6.43 ACMRS – 19.29 AFM – 6.43 DFM—3.058 MAAISU – 2.79 MAETC – 45.36 MAHEM – 48.81 MAISU – 1.6 MALYS + 4.73 MATCA) and to discover that the term involving ACMRS was most responsible for the blow up. We then examined the rate-law expression for ACMRS, and discovered that growth in [MRS](t) and [ETC](t) were most responsible for the rate increase of ACMRS. The ODEs controlling these two concentrations were: [MRS]´(t) = 6.43 BMRS – 6.43 DMRS and [ETC]´(t) = 6.43 MAETC – 6.43 ETCD – 6.43 DETC. The rate-law expression for MAETC suggested that the concentration [aETC](t) had the greatest influence on the growing rate of MAETC. The ODE governing the evolution of [aETC](t) ([aETC]´(t) = BAETC – MAETC – DAETC) suggested that the instability might be minimized or eliminated by regulating the biosynthesis reactions BMRS and BAETC. Rerunning the dynamic simulation with these regulatory modifications confirmed that they prevented the blow up of [FM](t). A similar process of investigation was used to identify the other four biosynthesis reactions that needed regulation in order to stabilize the *wt* steady-state ***U***_***ss(wt)***_.

**Fig. 4:**
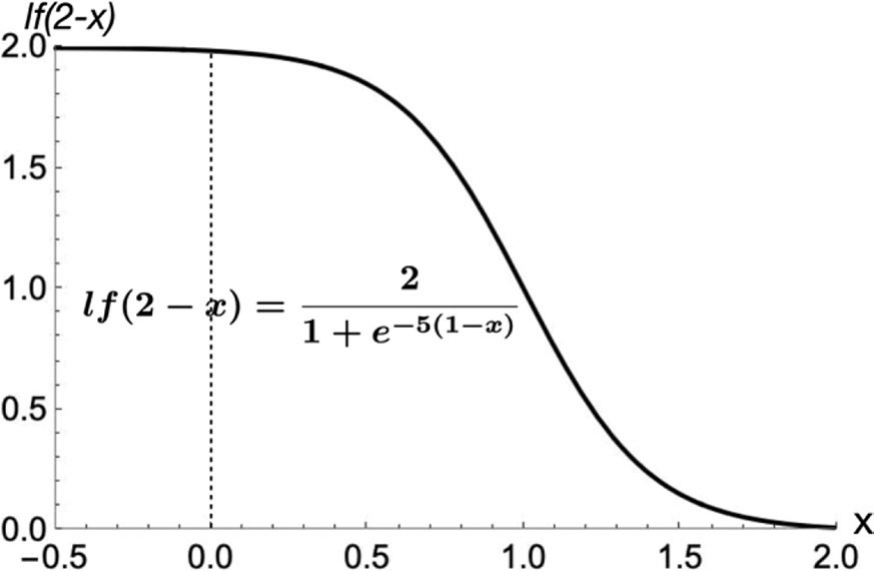
Logistic function defined by (24)

The regulation used here took the form of a logistic function that dampens the rate of the affected reactions when certain key component concentrations depart too fast from their initial steady-state values. The logistic function *lf(x)* had the form

24 $$lf(x) = \frac{2}{{1 + e^{ - 5(x - 1)} }}$$

where *x* is a function involving the component of the model that is being sensed. Figure 4 shows the graph of lf(2-x), the form used to regulate reactions, over the interval − *0.5* < *x* < *2.* The value 5 in the exponent, which controls the slope of the function, was empirically assigned to create the desired steepness of the curve.

These functions were used to regulate biosynthesis reactions *BAAFT, BAETC, BAFT5, BAGRX, BAHMX* and *BMRS.* In biological terms, this was tantamount to regulating the expression of the genes/proteins produced by those reactions. The regulation of *BAAFT* consisted of multiplying its rate-law expression in Table S11 by *lf(2-[FC(t)]/[FC*_*ss*_*])* where *[FC*_*ss*_*]* denotes the steady-state value of *[FC]* in ***U***_*ss*_. In similar fashion, *BAETC* was regulated by *lf(2-[*ETC*(t)]/[ETC*_*ss*_*])*, *BAFT5* by *lf(2-[aFT5(t)]/[aFT5*_*ss*_*])*, *BAGRX* by *lf(2-[GRX(t)]/[GRX*_*ss*_*])*, and *BAHMX* by *lf(2-[aHMX(t)]/[aHMX*_*ss*_*])*. *BMRS* was double regulated by the product *lf(2-[FM(t)]/[FM*_*ss*_*])⋅ lf(2-[HEME(t)]/[HEME*_*ss*_*]).* In biological terms, this is tantamount to assuming that the expression of: a) AFT (Aft1/2) is regulated by the cytosolic labile Fe pool; b) the electron transport chain complexes are autoregulated; c) FET5 (vacuolar iron exporter) is regulated by its apo form; d) GRX (glutaredoxins Grx3/4) is autoregulated; d) heme oxygenase is autoregulated; and e) MRS (Mrs3/4, importers of iron into mitochondria) is regulated by the labile iron pool in mitochondria and also by free heme in the cytosol. All of these imposed regulatory effects should be viewed as predictions of the model as they require experimental confirmation.

**Fig. 5:**
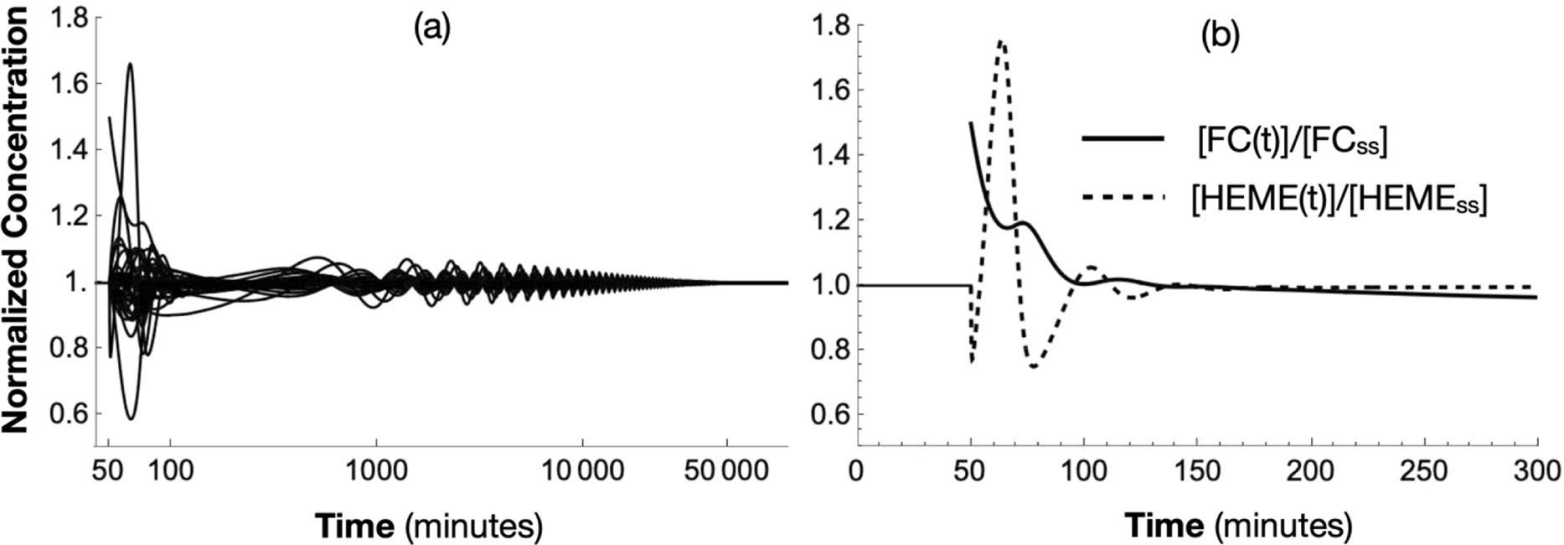
Normalized solution U(t)/Uss to (23) due to a 50% increase in [FC(0)] (**a)** at t = 50 min and normalized concentration [FC(t)]/[FCss] versus [HEME(t)]/[HEMEss] (**b)**

This regulatory strategy kept ***U***_***ss(wt)***_ as a locally stable steady-state of the dynamical system (23). To illustrate this stability, consider the system (23) with initial state given by ***U***_***ss(wt)***_. The system is stable (Fig. 5) until the concentration of *[FC]* is increased by 50% at t = 50 min*.* This figure graphs the solution ***U(t)*** to (23), normalized by ***Uss(wt)*** and defined to be ***U(t)/Uss***, where division of the two vectors is interpreted as being component-wise. This primary perturbation influences the concentrations of all other components in the in silico cell (as a secondary response) in a diversity of ways, illustrating the interconnectivity of all components within the reaction network. Then, as the cell grows, it recovers and returns to the original ***U***_***ss(wt)***_ state, showing the robust stability of the ***U***_***ss(wt)***_ state and the entire ODE system. A similar response is obtained when the concentration of [FS] is abruptly decreased by 50%. Figure 5b shows the dynamical behavior of the normalized concentrations of *FC* and *HEME* with the concentration of *HEME* showing the greatest effect from the initial *50%* increase of *[FC].*

The regularized model was used as well to study the response of the system (23) to perturbations in nutrient levels IRON and OXYGEN, and to perturbations in reaction rate constants *kACMRS* and *kMATCA*. When nutrient levels and rate-constants are perturbed in (23), ***U***_***ss(wt)***_ is no longer a system steady-state. Large nonlinear dynamical systems such as (23) can exhibit a broad range of responses to changes in system parameters. Solutions can converge to a new steady-state, or converge to a periodic orbit (Hopf bifurcation, e.g.), exhibit blowup or chaotic behavior. For all of the examples presented below, the initial state for the system (23) is taken to be the ***U***_***ss(wt)***_ state.

**Fig. 6:**
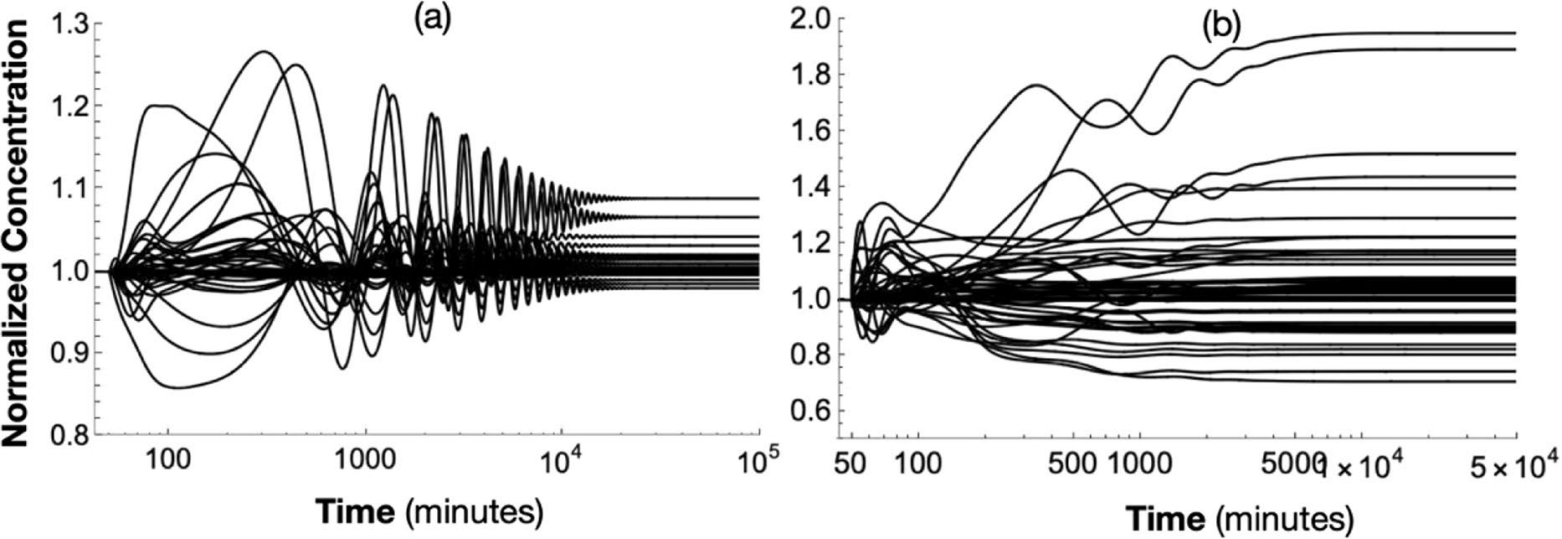
Normalized solutions to (23) due to a 20% decrease in IRON (**a)** at t = 50 min and a 20% decrease in OXYGEN (**b)**

Figure 6a and b illustrate the normalized solution to (23) resulting from a *20%* decrease in nutrient IRON and a *20%* decrease in nutrient OXYGEN, respectively at t = 50 min. In both cases, the dynamics converge to new steady-states, as given in Table S12. The graphs illustrate the unrealistically long times required for system (23) to stabilize. Reactions that are not included in the assumed network likely speed recovery rates in real cells.

**Fig. 7:**
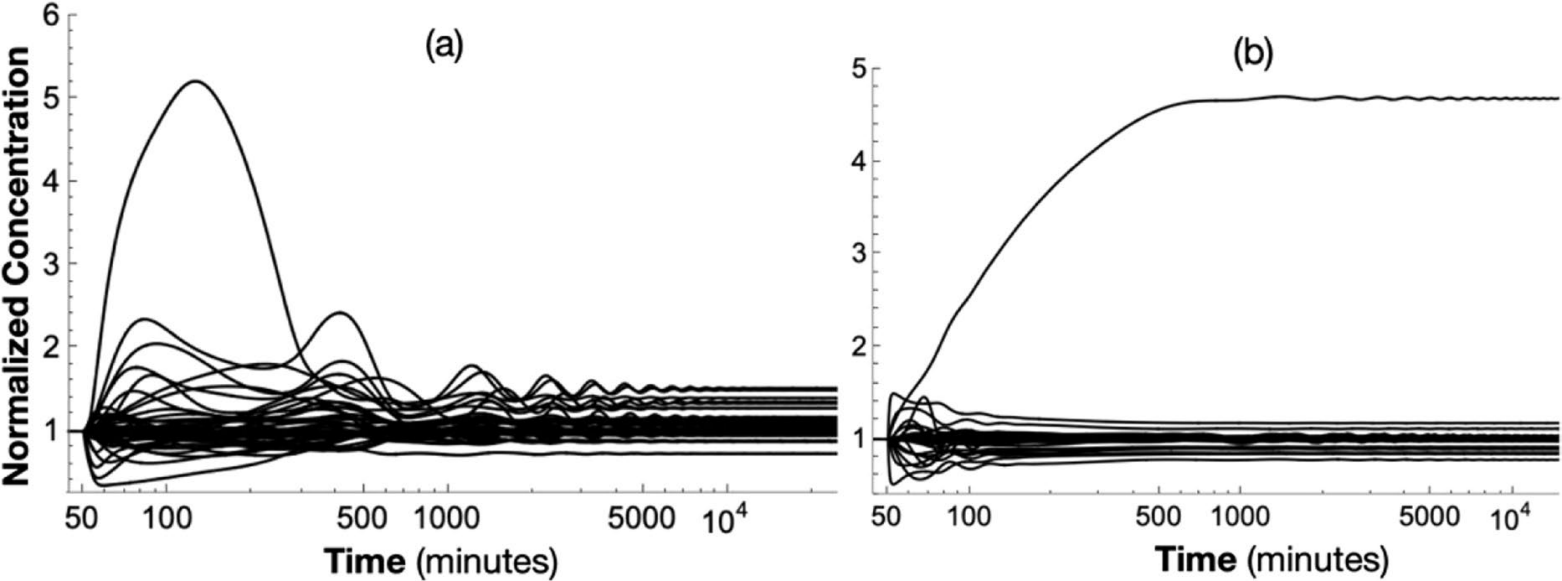
Normalized solution to (23) due to a 50% decrease in kACMRS (**a)** and a 50% decrease in kMATCA (**b)**

Figure 7a,b shows the normalized response of the solution to (23) due to a *50%* decrease in *kACMRS* and *kMATCA*, respectively at t = 50 min. The system converges to the steady-states given in Table S13. In Fig. 7b, one sees that the *50%* decrease in *kMATCA* gives rise to a *460%* increase in the steady-state concentration of the component *aTCA.* One readily observes that this unrealistically large increase of the steady-state concentration of *aTCA* is due to the unregulated reaction *BATCA.* It was then shown that applying logistic regulation, as a function of *[aTCA](t)/[aTCA]*_*ss*_, to the rate-law expression for *BATCA* prevents this unrealistically large blow up in the steady-state concentration of *aTCA,*

### Model verification

Due to its size and complexity, verifying the in silico cell model systematically was not possible so we focused on the predicted iron content of the cell under various conditions. This involved multiplying the simulated cellular steady-state concentration attained for each component under various perturbations by the iron content of the component (Table S15), and then summing all contributions. For WT conditions (**U**_**ss(wt)**_), the sum of cellular iron concentrations was 556 µM, similar to those determined by Morales et al (2010) and Holme-Hampton et al. (2013). About 45% of cellular iron was found as F3 (vacuolar Fe^III^), as observed for iron-replete conditions. The following components were major contributors to cellular iron content: TCA (20% of cellular Fe), FC (10%), LEU (5%), ETC (4%), and LYS (4%). Compartmental contributions for cytosol (105 µM), mitochondria (166 µM), vacuoles (275 µM), endoplasmic reticulum (7 µM) and nuclei (3 µM) are near to those iron concentrations determined/estimated from Mössbauer/proteomics results (Lindahl and Vali 2022).

We assessed changes due to a 20% decrease in nutrient IRON by considering the ratio of steady-state concentrations for each component. The ratio for aAFT (the apo form of the AFT regulator) increased slightly (compare Tables S3 vs. S14), consistent with experiments showing that this form activates the iron regulon under Fe-deficient conditions. The ratio for FT3 (Fe importer on the plasma membrane) declined nearly 7%, and the ratio for FC (cytosolic labile Fe pool), F2 (vacuolar Fe^II^), and F3 increased by ~ 4%, 2%, and 3% respectively. Ratios of virtually all Fe-containing proteins (except CIA) were > 1, which was either consistent with experimental results or made intuitive sense.

Under hypoxic conditions, the reduced form of vacuolar iron (F2) in real cells increases (Fernandez 2023). The model simulates this, with F2 increasing 54% when nutrient OXYGEN decreases 20%. O2 promotes the degradation of CAT and ETC, generating FC as iron is released. Under hypoxic conditions, these reactions should be attenuated, causing CAT and ETC to increase and FC to decline, as observed. Other changes due to hypoxic conditions were more difficult to explain. For example, we had expected ATP and ROS levels to decline, but the former increased and the latter group was unchanged.

## Discussion

In this study, an ODE-based kinetic model of iron metabolism in a respiring iron-replete yeast cell was developed using the recently developed basic-pathways approach (Walton and Lindahl 2023). The model, interpreted as an in silico growing yeast cell, including 80 components interacting in a network of 169 reactions, included all aspects of iron metabolism at a level of detail that *approached* (but did not attain) genome scale. The more than 100 iron-containing proteins and complexes in yeast cells represented in the model were organized into 23 protein groups and 7 nonproteinaceous complexes. The cell had realistic dimensions and was divided into established sub-compartments including cytosol, mitochondria, vacuoles, nucleus, and endoplasmic reticula. Carbon-based central metabolism was included as a means of providing a function for each iron-containing group (see discussion of Mutual Autocatalysis below). These carbon-based background or housekeeping processes were included in outline forms in which many reactions were combined; thus, they should not be considered to be realistic or that their derived rates are accurate.

As is typical for complex biochemical reaction networks operating within cells, we had limited knowledge of: a) the exact reactions involved; b) the rate-law expressions for the reactions; c) the magnitudes of essential kinetic parameters such as rate-constants and Michaelis–Menten parameters; and d) component steady-state concentrations. Despite these limitations, best estimates were made and the system was solved under steady-state growth conditions, as would occur in exponentially growing cell cultures. Experimental data associated with that state (growth rates, whole-cell and organellar iron concentrations, Mössbauer spectra of whole-cells and isolated organelles, and steady-state concentrations for various component) (Hudder et al. 2007; Morales et al. 2010; Holmes-Hampton, 2010; Holmes-Hampton 2013) were used. Each reaction was stoichiometrically balanced in terms of iron (as exact as possible) and carbon (approximate). Analyzing the stoichiometric matrix using the Basic Pathways approach allowed us to identify a complete set of chemically-meaningful non-negative steady-state rates. *Dependent* rates were distinguished from *independent* ones, and particular reaction rates were strategically assigned to each category. Known rates (such as those associated with dilution and those expected to be 0) were generally defined to be independent. Rates of 7 non-dilution independent reactions had to be guessed. This was the least reliable aspect of the approach, but it also highlighted the reactions whose rates would be most profitably measured in future studies. Once all independent rates were assigned, dependent rates were immediately calculated and the steady-state system was solved. From these steady-state rates, the associated steady-state dilution reactions were used to form the WT steady-state component vector ***U***_***ss(wt)***_.

To solve the dynamical system, generic rate-law expressions for the reactions and *K*_*m*_ values for Michaelis–Menten-like terms were assigned according to simple explicit rules which allowed corresponding rate-constants to be calculated. With all kinetic parameters assigned or approximated, the nonlinear dynamical ODE system was solved numerically using ***U***_***ss(wt)***_ as the initial state. However, steady-state ***U***_***ss(wt)***_ was not locally stable; for a time the solution ***U(t)*** of (23) remained equal to its initial state ***U***_***ss(wt)***_ but eventually accumulated roundoff error caused that solution to depart from the steady-state ***U***_***ss(wt)***_. Due to mutual autocatalytic processes in the dynamical system (23), the instability associated with ***U***_***ss(wt)***_ elicited either blow-up or Hopf bifurcation.

Through observing which components and reactions initiated the instability associated with ***U***_***ss(wt)***_, judicious use of logistic regulatory functions applied to the six reactions *BAAFT, BAETC, BAFT5, BAGRX, BAHMX* and *BMRS* stabilized the dynamical system (23). This was illustrated by showing that the solution ***U(t)*** of (23) with ***U***_***ss(wt)***_ as initial state for all components except for *FC(0)* which given a *50%* increase or decrease from its steady-state value in ***U***_***ss(wt)***_ quickly converged back to ***U***_***ss(wt)***_ after a rather complex initial transient phase. The initial transient phase of *HEME* and *aMEM* showed the greatest perturbation from their initial values of any other components which is not surprising given the form of the right-hand-side of the ODEs for *HEME(t)* and *aMEM(t)* in (23).

The regulated system (23) was then used to assess the sensitivity of the solution ***U(t)*** to perturbations in system parameters. Changes to system parameters resulted in ***U***_***ss(wt)***_ no longer being a steady-state of (23). What was observed was that changes to system parameters, keeping ***U***_***ss(wt)***_ as the initial state, can result in the solution ***U(t)***: (a) converging to a steady-state different from ***U***_***ss(wt)***_; (b) blowup; (c) Hopf bifurcation. Chaotic behavior was not observed.

### *Sloppy* models

Large numbers of unknown or uncertain kinetic parameters plague virtually all models of complex biochemical processes occurring within a cell. For these reasons, reaction networks of this complexity and uncertainty are typically solved using constraint-based approaches. However, Gutenkunst et al. (2007) have argued that precise determinations of kinetic parameters may not be as critical as is often assumed. They evaluated 17 published models in which collective fitting yielded well-constrained and valid predictions, even though individual parameters were poorly constrained or unknown. Gutenkunst et al. suggested that modelers facing such problems should focus on collective phenotypic predictions rather than on rigorous parameter determinations, arguing that models that are incorrect at some level of detail may still help understand high-level cellular behavior. They suggested that, despite the difficulties, modelers should build “*incomplete tentative falsifiable models in the most expressive and predictive fashion feasible*”, which we have done here. Future studies can now be conducted to test the model against predicted mutant phenotypes and growth conditions, and then modify/expand it as needed to improve fidelity to those perturbing conditions, as well as to improve model stability and robustness.

### Mutual autocatalysis

Living systems are membrane-encapsulated mutual autocatalysts in which their components collectively facilitate the process of self-replication (Wächtershäuser 1988; Lindahl 2004). Designing in silico cells that possess this essential characteristic would provide an additional constraint in developing viable whole-cell models. Consistent with autocatalytic growth, every ODE included a dilution term reflecting cell growth. We attempted to include mutual autocatalysis by requiring that the components of the reaction network be heavily *interrelated*. We designed each component (with a few exceptions) to influence and be influenced by at least one other component of the system. Reactants or catalysts of a reaction serve to influence the products of the reaction. Exceptions included nutrients which influenced other components but were not influenced by them, and waste products which were influenced by components but did not influence them. A few components were *antagonists* to cell growth, including ROS in all of its forms. The protein group PRO had no function apart from consuming precious metabolites such as ATP and amino acids during its biosynthesis. Generating these influences between components required that various carbon-based housekeeping functions and components be included. These efforts to generate a mutual autocatalytic system may have inadvertently generated some destabilizing feedback loops. More work is required to understand how to include this fundamental property without negatively impacting overall stability.

### Connection to iron metabolism

Steady-state concentrations for each component of the model, especially those involving iron-containing species, were assigned to be consistent with experimentally determined concentrations as measured using Mössbauer spectroscopy and analytically-determined iron concentrations. In essence, rates and rate-constants were found that largely ensured these iron-associated concentrations under steady-state WT conditions. Under the conditions assumed (aerobic respiring *wt* yeast cells grown with 40 µM iron in the medium), the steady-state iron concentrations “predicted” by the model for the entire cell, for each organelle, and for each individual iron-containing species were near to those determined experimentally. Moreover, the model distinguished different *types* of iron centers, as well as realistic mechanisms for their trafficking, assembly into proteins, and regulation. On the other hand, the model lacked degradation processes for any cellular component. Thus, recovery rates from perturbations would be faster than those calculated here if products decomposed faster than only by dilution due to cell growth. This may be especially problematic for iron-sulfur proteins, as their clusters tend to be kinetically labile (Thompson et al 2023).

### Comparison to previous models

Our earlier models included on the order of 10 components and about twice as many reactions. They typically involved three cellular compartments, including cytosol, mitochondria, and vacuoles (Wofford and Lindahl 2015; Wofford and Lindahl (2019); Fernandez et al. 2022; Thorat et al. 2023). The objective of those studies was to highlight iron metabolism in mitochondria, especially trafficking and regulation. We were mainly interested in the effect of Yfh1 (yeast frataxin homolog) deletion and the effects of hypoxia, as this relates closely to the disease Friedreich’s ataxia. The objective of the current model was far greater in scope, namely to describe all the various iron-related processes occurring in a yeast cell and show how they interact as a unified integrated system in the cell. It represents the first *comprehensive integrative mechanism of iron metabolism* in a growing yeast cell.

The model of Chen et al. (2021), called CofactorYeast, may be the most comparable to our model. It includes iron metabolism comprehensively, and at a comparable level of detail. The greatest difference is that they used constraint-based modeling and flux-balance-analysis whereas we solved a system of ODEs to generate a stable dynamical system. Both models included many of the same processes (protein synthesis, different types of iron centers, metallation, the TCA cycle, amino acid biosynthesis, ergosterol/phospholipid/membrane biosynthesis, and cell growth/dilution). Both models estimated the iron concentration in whole cells and in individual proteins, as well as changes caused by iron deficiency. Chen introduced a flexible parameter θ to adjust the rate of enzyme activity according to the occupancy of the metal; we assumed simple rules for assigning iron occupancies. The Chen model underestimates cellular Fe concentrations because it does not include nonproteinaceous labile iron pools; our model includes 7 such pools and simulates about half of total cellular Fe to be present as non-proteinaceous Fe in vacuoles, as is observed for iron-replete conditions. The Chen model predicts that Lys4 represents about half of cellular iron whereas our model predicts that the LYS group contributes only 8% of cellular protein-bound iron. The Chen model assumes that all iron cofactors are generated in the cytosol; our model correctly assumes that Fe/S clusters are primarily assembled in the mitochondria, with a portion exported to cytosol.

### A mechanistic underpinning for omics data

Abundant transcriptomics, metabolomics, and proteomics datasets are routinely generated in cell biological studies, yet molecular-level mechanistic analyses of such datasets are lacking. As a result, genetic phenotypes can often only be characterized empirically, phenomenologically, or statistically. This is often sufficient for understanding the primary cause of a genetic mutation but not for understanding how a mutation affects the entire cell *as a system*. The dynamical model developed here could, in principle, be used to analyze omics data on the system’s level, as it could, in principle, predict the complete set of component concentrations expected by mutating any gene/protein in the system. Similarly, changes in overall cellular properties due to changes in growth conditions are currently treated empirically or only as local effects (e.g. cellular iron increases when iron is added to the growth media). Again, dynamical models could simulate how such environmental changes affect cellular metabolism as a system. More advanced and detailed models of iron metabolism are required to accurately predict such changes. However, the ability to solve such a complicated system dynamically as we have done here with limited information opens-up an opportunity to predict the overall cellular effects of a multitude of genetic states and growth conditions.

## Supplementary Information

Below is the link to the electronic supplementary material.

Supplementary file1 (DOCX 701 KB)

## Data Availability

All relevant data are within the main text and Supporting Information.

## Abbreviations

ER: endoplasmic reticulum
GEMs: genome-scale models
ISC: iron-sulfur cluster
ODE: ordinary differential equation
***R***_***cell***_: vector of reaction rates
ROS: reactive oxygen species
***S***_***cell***_: stochiometric matrix
*wt*: wild-type
