# Supplementary material for: A kinetic mathematical model of comprehensive iron metabolism in a respiring yeast cell: a basic-pathways approach to solving a large system dynamically
Source: Biometals. 2025 Dec 10;39(1):231–57. doi: 10.1007/s10534-025-00758-7 (PMC12852300; doi:10.1007/s10534-025-00758-7)
Supplement: Supplementary file 1 — Supplementary file1 (DOCX 701 KB) [file 10534_2025_758_MOESM1_ESM.docx]

**Supplemental Information**

Title: A kinetic mathematical model of comprehensive iron metabolism in a respiring yeast cell: a basic-pathways approach to solving a large system dynamically

Authors: P.A. Lindahl and J.R. Walton

**Table of Contents:**

Table S1: Iron-containing proteins in *S. cerevisiae*.

Table S2: Cell compartments and volumes

Table S3: Component concentrations

Table S4: Derivation of protein groups

Table S5: Derivation of iron content of protein groups.

Table S6: Summary of iron centers and amino acid molar equivalents for protein groups.

Table S7: Summary of iron and carbon content for all components.

Table S8: Derivation of amino acid content of protein groups from members.

Table S9: Model reactions.

Table S10: Steady-state rates for each reaction.

Table S11: Assumed rate-law expressions for each reaction.

Table S12: Change in component concentrations due to a 50% decrease in kACMRS.

Table S13: Change in component concentrations due to a 50% decrease in kMATCA.

Table S14: Change in component concentrations due to a 20% decrease in IRON.

Table S15: Change in component concentrations due to a 20% decrease in OXYGEN.

Table S16: Steady-State iron concentrations generated by the model.

Appendix A: Justification for organizing protein groups

Appendix B: Independent reaction-rate selections and compatibility relationships.

Appendix C: Basic Pathways expansion of general pathway.

Separate SI Documents:

**Smatrix:** Stoichiometric matrix (***S_cell_***). Columns refer to reactions, rows to components. Interregional reactions are color-coded, with volume ratios indicated. For example, column B describes interregional reaction AATM involving cytosol and mitochondria. Stoichiometric coefficients are explained in Table S9, right side.

**Wmatrix:** The ***W*** matrix used in this study.

**BasicPathways:** An illustration of the BPs obtained from the W matrix.

**ReactionList.txt:** A complete list of reactions including the name of each reaction, the reaction itself, and any catalysts. The equal sign should be interpreted as a forward arrow.

**Table S1: Known or suspected iron-containing proteins in *S. cerevisiae*.** The list of 117 Fe proteins were obtained from the database of Lindahl and Vali (2022) and supplemented by Table S2 of Chen et al. (2021). Also included are the types of iron center in the protein. The 15 entries called “guests” were not included in the developed model. References provide an entry to the protein.

| Number | Member (guest) | Identifier | C_i(F4)_ | C_i(F3)_ | C_i(F2)_ | C_i(HE)_ | C_i(FF)_ | C_i(FO)_ | Reference |
| --- | --- | --- | --- | --- | --- | --- | --- | --- | --- |
| 1 | Aco1 | YLR304C | 1 | 0 | 0 | 0 | 0 | 0 | Lindahl and Vali (2022) |
| 2 | Aco2 | YJL200C | 1 | 0 | 0 | 0 | 0 | 0 | ″ |
| 4 | Aft1 | YGL071W | 0 | 0 | 0.5 | 0 | 0 | 0 | ″ |
| 5 | Aft2 | YPL202C | 0 | 0 | 0.5 | 0 | 0 | 0 | ″ |
| 6 | Aim32 | YML050W | 0 | 0 | 1 | 0 | 0 | 0 | ″ |
| 7 | Apd1 | YBR151W | 0 | 0 | 1.0 | 0 | 0 | 0 | ″ |
| 8 | Ate1 (guest) | YGL017W | 1 | 0 | 0 | 0 | 0 | 0 | Van et al. 2023 |
| 9 | Bio2 | YGR286C | 1 | 0 | 0 | 0 | 0 | 0 | Lindahl and Vali (2022) |
| 10 | Bna1 | YJR025C | 0 | 0 | 0 | 0 | 0 | 1 | ″ |
| 11 | Bna2 (guest) | YJR078W | 0 | 0 | 0 | 1 | 0 | 0 | Chen et al. 2021 |
| 12 | Bol1 | YAL044W-A | 0 | 0 | 0.5 | 0 | 0 | 0 | Lindahl and Vali (2022) |
| 13 | Bol2 | YGL220W | 0 | 0 | 0.5 | 0 | 0 | 0 | ″ |
| 14 | Bol3 | YAL046C | 0 | 0 | 0.5 | 0 | 0 | 0 | ″ |
| 15 | Ccp1 | YKR066C | 0 | 0 | 0 | 2 | 0 | 0 | ″ |
| 16 | Cfd1 | YIL003W | 0.5 | 0 | 0 | 0 | 0 | 0 | ″ |
| 17 | Chl1 | YPL008W | 1 | 0 | 0 | 0 | 0 | 0 | ″ |
| 18 | Cir2 (guest) | YOR356W | 1 | 0 | 0 | 0 | 0 | 0 | Chen et al. 2021  Leo et al. 2018 |
| 19 | Cob1 | Q0105 | 0 | 0 | 0 | 2 | 0 | 0 | Lindahl and Vali (2022) |
| 20 | Coq7 | YOR125C | 0 | 0 | 0 | 0 | 1 | 0 | ″ |
| 21 | Cox1 | Q0045 | 0 | 0 | 0 | 2 | 0 | 0 | ″ |
| 22 | Cox10 | YPL172C | 0 | 0 | 0 | 1 (uncertain) | 0 | 0 | ″ |
| 23 | Cox15 | YER141W | 0 | 0 | 0 | 2 (uncertain) | 0 | 0 | ″ |
| 24 | Cta1 | YDR256C | 0 | 0 | 0 | 1 | 0 | 0 | ″ |
| 25 | Ctt1 | YGR088W | 0 | 0 | 0 | 1 | 0 | 0 | ″ |
| 26 | Cyb2 | YML054C | 0 | 0 | 0 | 1 | 0 | 0 | ″ |
| 27 | Cyb5 | YNL111C | 0 | 0 | 0 | 1 | 0 | 0 | ″ |
| 28 | Cyc1 | YJR048W | 0 | 0 | 0 | 1 | 0 | 0 | ″ |
| 29 | Cyc7 | YEL039C | 0 | 0 | 0 | 1 | 0 | 0 | ″ |
| 30 | Cyp51 | YHR007C | 0 | 0 | 0 | 1 | 0 | 0 | ″ |
| 31 | Cyt1 | YOR065W | 0 | 0 | 0 | 1 | 0 | 0 | ″ |
| 32 | Dap1 | YPL170W | 0 | 0 | 0 | 1 | 0 | 0 | ″ |
| 33 | Dbr1 | YKL149C | 0 | 0 | 0 | 0 | 0 | 1 | ″ |
| 34 | Dit2 (guest) | YDR402C | 0 | 0 | 0 | 1 | 0 | 0 | Chen et al. 2021  Leo et al. 2018 |
| 35 | Dna2 | YHR164C | 1 | 0 | 0 | 0 | 0 | 0 | Lindahl and Vali (2022) |
| 36 | Dph1 | YIL103W | 0.5 | 0 | 0 | 0 | 0 | 0 | ″ |
| 37 | Dph2 | YKL191W | 0.5 | 0 | 0 | 0 | 0 | 0 | ″ |
| 38 | Dph3 | YBL071W-A | 0 | 0 | 0 | 0 | 0 | 1 | ″ |
| 39 | Dph4 | YJR097W | 0 | 0 | 0 | 0 | 0 | 1 | ″ |
| 40 | Dre2 | YKR071C | 0.5 | 0 | 1.5 | 0 | 0 | 0 | ″ |
| 41 | Elp3 | YPL086C | 1 | 0 | 0 | 0 | 0 | 0 | ″ |
| 42 | Erg3 | YLR056W | 0 | 0 | 0 | 0 | 1 | 0 | ″ |
| 43 | Erg5 | YMR015C | 0 | 0 | 0 | 1 | 0 | 0 | ″ |
| 44 | Erg25 | YGR060W | 0 | 0 | 0 | 0 | 1 | 0 | ″ |
| 45 | Exo5 | YBR163w | 1 | 0 | 0 | 0 | 0 | 0 | ″ |
| 46 | Fmp12 (guest) | YHL021C | 0 | 0 | 0 | 0 | 0 | 1 | Chen et al. 2021  Nishida et al. 2016 |
| 47 | Fre1 | YLR214W | 0 | 0 | 0 | 2 | 0 | 0 | Lindahl and Vali (2022) |
| 48 | Fre2 | YKL220c | 0 | 0 | 0 | 1 | 0 | 0 | ″ |
| 49 | Fre3 | YOR381w | 0 | 0 | 0 | 1 | 0 | 0 | ″ |
| 50 | Fre4 | YNR060w | 0 | 0 | 0 | 1 | 0 | 0 | ″ |
| 51 | Fre5 | YOR384w | 0 | 0 | 0 | 1 | 0 | 0 | ″ |
| 52 | Fre6 | YLL051C | 0 | 0 | 0 | 2 | 0 | 0 | ″ |
| 53 | Fre7 | YOL152w | 0 | 0 | 0 | 1 | 0 | 0 | ″ |
| 54 | Fre8 | YLR047c | 0 | 0 | 0 | 1 | 0 | 0 | ″ |
| 55 | Glt1 | YDL171C | 2 | 1 | 0 | 0 | 0 | 0 | ″ |
| 56 | Grx3 | YDR098C | 0 | 0 | 0.5 | 0 | 0 | 0 | ″ |
| 57 | Grx4 | YER174C | 0 | 0 | 0.5 | 0 | 0 | 0 | ″ |
| 58 | Grx5 | YPL059W | 0.5 | 0 | 1.5 | 0 | 0 | 0 | ″ |
| 59 | Grx6 | YDL010W | 0 | 0 | 0.5 | 0 | 0 | 0 | ″ |
| 60 | Hap1 | YLR256W | 0 | 0 | 0 | 1 | 0 | 0 | ″ |
| 61 | Hap4 | YKL109W | 0 | 0 | 0 | 1 | 0 | 0 | ″ |
| 62 | Hem15 | YOR176W | 0 | 0 | 0 | 1 | 0 | 0 | ″ |
| 63 | Hmx1 | YLR205C | 0 | 0 | 0 | 1 | 0 | 0 | ″ |
| 64 | Ilv3 | YJR016C | 0 | 0 | 1 | 0 | 0 | 0 | ″ |
| 65 | Isa1 | YLL027W | 0.5 | 0 | 0.5 | 0 | 0 | 0 | ″ |
| 66 | Isa2 | YPR067W | 0.5 | 0 | 0.5 | 0 | 0 | 0 | ″ |
| 67 | Isu1 | YPL135W | 0 | 0 | 1 | 0 | 0 | 0 | ″ |
| 68 | Isu2 | YOR226C | 0 | 0 | 1 | 0 | 0 | 0 | ″ |
| 69 | Jhd1 (guest) | YER051W | 0 | 0 | 0 | 0 | 0 | 1 | Chen et al. 2021  Jia et al. 2007 |
| 70 | Jhd2 (guest) | YJR119C | 0 | 0 | 0 | 0 | 0 | 1 | Chen et al. 2021  Shapiro et al. 2023  Soloveychik et al. 2016 |
| 71 | Jlp1 (guest) | YLL057C | 0 | 0 | 0 | 0 | 0 | 1 | Chen et al. 2021 |
| 72 | Leu1 | YGL009C | 1 | 0 | 0 | 0 | 0 | 0 | Lindahl and Vali (2022) |
| 73 | Lia1 | YJR070C | 0 | 0 | 0 | 0 | 0 | 1 | ″ |
| 74 | Lip5 | YOR196C | 2 | 0 | 0 | 0 | 0 | 0 | ″ |
| 75 | Lto1 | YNL260C | 0 | 0 | 0 | 0 | 0 | 0 | ″ |
| 76 | Lys4 | YDR234W | 1 | 0 | 0 | 0 | 0 | 0 | ″ |
| 77 | Met5 | YJR137C | 1 | 0 | 0 | 1 | 0 | 0 | ″ |
| 78 | Met8 | YBT213W | 0 | 0 | 0 | 0 | 0 | 0 | ″ |
| 79 | Mpo1 | YGL010W | 0 | 0 | 0 | 0 | 0 | 1 | ″ |
| 80 | Mss51 | YLR203C | 0 | 0 | 0 | 1 | 0 | 0 | ″ |
| 81 | Nar1 | YNL240C | 2 | 0 | 0 | 0 | 0 | 0 | ″ |
| 82 | Nbp35 | YGL091C | 1.5 | 0 | 0 | 0 | 0 | 0 | ″ |
| 83 | Ncs6 | YGL211W | 0 | 1 | 0 | 0 | 0 | 0 | ″ |
| 84 | Nfu1 | YKL040C | 0.5 | 0 | 0 | 0 | 0 | 0 | ″ |
| 85 | Ntg2 | YOL043C | 1 | 0 | 0 | 0 | 0 | 0 | ″ |
| 86 | Ole1 | YGL055W | 0 | 0 | 0 | 1 | 1 | 0 | ″ |
| 87 | Pol1 | YNL102W | 1 | 0 | 0 | 0 | 0 | 0 | ″ |
| 88 | Pol2 | YNL262W | 1 | 0 | 0 | 0 | 0 | 0 | ″ |
| 89 | Pol3 | YDL102W | 1 | 0 | 0 | 0 | 0 | 0 | ″ |
| 90 | Pri2 | YKL045W | 1 | 0 | 0 | 0 | 0 | 0 | ″ |
| 91 | Rad3 | YER171W | 1 | 0 | 0 | 0 | 0 | 0 | ″ |
| 92 | Rev3 | YPL167C | 1 | 0 | 0 | 0 | 0 | 0 | ″ |
| 93 | Rip1 | YEL024W | 0 | 0 | 1 | 0 | 0 | 0 | ″ |
| 94 | Rli1 | YDR091C | 2 | 0 | 0 | 0 | 0 | 0 | ″ |
| 95 | Rnr2 | YJL026W | 0 | 0 | 0 | 0 | 1 | 0 | ″ |
| 96 | RSM22 (guest) | YKL155C | 1 | 0 | 0 | 0 | 0 | 0 | Ast et al. 2024 |
| 97 | Scs7 | YMR272C | 0 | 0 | 0 | 1 | 1 | 0 | Lindahl and Vali (2022) |
| 98 | Sdh2 | YLL041C | 1 | 1 | 1 | 0 | 0 | 0 | ″ |
| 99 | Sdh3 | YKL141W | 0 | 0 | 0 | 0.5 | 0 | 0 | ″ |
| 100 | Sdh4 | YDR178W | 0 | 0 | 0 | 0.5 | 0 | 0 | ″ |
| 101 | Sfh5 | YJL145W | 0 | 0 | 0 | 1 | 0 | 0 | ″ |
| 102 | Shh3 (guest) | YMR118C | 0 | 0 | 0 | 1 | 0 | 0 | Chen et al. 2021 |
| 103 | Shh4 (guest) | YLR164W | 0 | 0 | 0 | 1 | 0 | 0 | Chen et al. 2021 |
| 104 | Sur2 | YDR297W | 0 | 0 | 0 | 0 | 1 | 0 | Lindahl and Vali (2022) |
| 105 | Tdh3 | YGR192C | 0 | 0 | 0 | “1” | 0 | 0 | ″ |
| 106 | Thi4 | YGR144W | 0 | 0 | 0 | 0 | 0 | 1 | ″ |
| 107 | Thi5 | YFL058W | 0 | 0 | 0 | 0 | 0 | 1 | ″ |
| 108 | Thi11 (guest) | YJR156C | 0 | 0 | 0 | 0 | 0 | 1 | Chen et al. 2021  Wightman and Meacock 2003 |
| 109 | Thi12 (guest) | YNL332W | 0 | 0 | 0 | 0 | 0 | 1 | Chen et al. 2021  Wightman and Meacock 2003 |
| 100 | Thi13 (guest) | YDL244W | 0 | 0 | 0 | 0 | 0 | 1 | Chen et al. 2021  Wightman and Meacock 2003 |
| 111 | Tpa1 | YER049W | 0 | 0 | 0 | 0 | 0 | 1 | Lindahl and Vali (2022) |
| 112 | Tyw1 | YPL207W | 2 | 0 | 0 | 0 | 0 | 0 | ″ |
| 113 | Yah1 | YPL252C | 0 | 0 | 1 | 0 | 0 | 0 | ″ |
| 114 | Yap5 | YIR018W | 0.5 | 0 | 1.5 | 0 | 0 | 0 | ″ |
| 115 | Yap7 (guest) | YOL028C | 0 | 0 | 1 | 0 | 0 | 0 | Chen et al. 2021  Rodrigues-Pousada 2019 |
| 116 | Yhb1 | YGR234W | 0 | 0 | 0 | 1 | 0 | 0 | Lindahl and Vali (2022) |
| 117 | Yno1 | YGL160w | 0 | 0 | 0 | 2 | 0 | 0 | ″ |

**Table S2:** **Cell Compartments and Volumes.** The table includes fractional and absolute volumes of compartments assumed for the *in silico* cell model.

| Compartment | Fraction Cellular Volume  (excluding cell wall) | Absolute compartment volume  (×10^-15^ L or ×1 µm^3^) | Membrane volume estimate  (×10^-15^ L or ×1 µm^3^) |
| --- | --- | --- | --- |
| Cell | *f_cell_* = 1.000 | 42.00 | 1.51 |
| C | *f_c_* = 0.643 | 27.00 | 0.35 |
| N | *f_n_* = 0.151 | 6.34 | 0.20 |
| V | *f_v_* = 0.082 | 3.44 | 0.07 |
| M | *f_m_* = 0.100 | 4.20 | 0.39 |
| E | *f_e_* = 0.024 | 1.01 | 0.50 |

**Table S3: Component concentrations in cells and compartments.** Each component was located in one of cellular compartments, including cytosol (C), mitochondria (M), vacuoles (V), nucleus (N), and endoplasmic reticulum (E). Concentrations below are for respiring WT cells grown under iron-sufficient conditions. Local concentrations were determined by dividing the corresponding cellular concentration by the fractional volume of the compartment (see Table S2). For some transport reactions (when a component moves from one cellular compartment to another), the model assumed that the rate of transport was related to the concentration ratio [Donor]/[Receiver] > 1. In cases where that ratio was < 1, local concentrations was redefined so that the ratio became > 1 while maintaining the conservation of matter.

“Fraction in the specified form” refers to protein groups that are found in more than one form. Group proteins that are permanently installed with iron were presumed to be 90% holo and 10% apo. Group proteins that transfer iron centers or are involved in regulation were presumed to be 50% holo and 50% apo. For protein groups with three forms, the fully apo (“aa”) form was assumed to be 10%, the partial apo (“a”) form 45%, and the holo form 45%. Concentrations of protein groups were taken from Table S4.

| Number | Component | Fraction in the specified form | Compartment | Steady State *Cellular* Concentrations (μM)  for WT Conditions used for normalization in  ***U_ss(wt)_*** | Steady-State *Local* Concentrations (μM) for WT Conditions used for normalization in ***U_ss(wt)_*** |
| --- | --- | --- | --- | --- | --- |
| 01 | AA | 1 | C | 4197 | 6527 |
| 02 | aaCIA | 0.1 | C | 0.01556 | 0.024199 |
| 03 | aAFT | 0.5 | C | 0.03278 | 0.05098 |
| 04 | aaISA | 0.1 | C | 0.03924 | 0.061026 |
| 05 | aaISU | 0.1 | C | 0.11772 | 0.183079 |
| 06 | AAM | 1 | M | 979 | 9790 |
| 07 | ACA | 1 | M | 8.2 | 82 |
| 08 | ACAC | 1 | C | 91.8 | 142.7683 |
| 09 | aCAT | 0.1 | C | 0.045643 | 0.0709844 |
| 10 | aCIA | 0.45 | C | 0.0700362 | 0.108921 |
| 11 | ADP | 1 | C | 551 | 856.9207 |
| 12 | ADPM | 1 | M | 49 | 490 |
| 13 | aETC | 0.1 | C | 0.144868 | 0.2253 |
| 14 | AFT | 0.5 | C | 0.03278 | 0.05098 |
| 15 | aFT3 | 0.5 | C | 0.046177 | 0.071815 |
| 16 | aFT5 | 0.5 | C | 0.06505 | 0.101166 |
| 17 | aGRX | 0.5 | C | 0.10007 | 0.15563 |
| 18 | aHEM | 0.5 | C | 0.127297 | 0.1979735 |
| 19 | aHMX | 0.1 | E | 0.01213 | 0.5054167 |
| 20 | aISA | 0.45 | M | 0.17658 | 1.7658 |
| 21 | aISU | 0.45 | M | 0.52974 | 5.2974 |
| 22 | aLEU | 0.1 | C | 0.09236 | 0.143639 |
| 23 | aLYS | 0.1 | C | 0.180952 | 0.281418 |
| 24 | aMEM | 0.1 | C | 0.0317858 | 0.0494336 |
| 25 | aNUC | 0.1 | C | 0.110745 | 0.172232 |
| 26 | aPOL | 0.1 | C | 0.0103512 | 0.016098 |
| 27 | aRIB | 0.1 | C | 0.025241 | 0.039255 |
| 28 | aTCA | 0.1 | C | 0.412449 | 0.641445 |
| 29 | ATM | 1 | M | 0.17841 | 1.7841 |
| 30 | ATP | 1 | C | 2018.7 | 3139.502 |
| 31 | ATPM | 1 | M | 180.5 | 1805 |
| 32 | aYAP | 0.5 | C | 0.01204 | 0.079735 |
| 33 | CAT | 0.9 | C | 0.410787 | 0.638860 |
| 34 | CCC | 1 | V | 0.14328 | 1.747317 |
| 35 | CIA | 0.45 | C | 0.0700362 | 0.108921 |
| 36 | CO2 | 1 | C | 100000 | 155520.9953 |
| 37 | CP | 1 | C | 0 | 0 |
| 38 | DNA | 1 | N | 0.248 | 1.642384 |
| 39 | ETC | 0.9 | M | 1.303812 | 13.03812 |
| 40 | F2 | 1 | V | 25 | 304.8780 |
| 41 | F3 | 1 | V | 250 | 3048.7805 |
| 42 | FC | 1 | C | 54 | 83.98134 |
| 43 | FT3 | 0.5 | C | 0.046177 | 0.071815 |
| 44 | FT5 | 0.6 | V | 0.06505 | 0.79329268 |
| 45 | FM | 1 | M | 9 | 90 |
| 46 | GRX | 0.5 | C | 0.10007 | 0.15563 |
| 47 | HEM | 0.5 | C | 0.127297 | 0.1979735 |
| 49 | HEME | 1 | C | 0.03 | 0.0466563 |
| 48 | HMX | 0.9 | E | 0.109136 | 4.547325 |
| 50 | ISA | 0.45 | M | 0.17658 | 1.7658 |
| 51 | ISU | 0.45 | M | 0.52974 | 5.2974 |
| 52 | LEU | 0.9 | C | 0.83123 | 1.292737 |
| 53 | LYS | 0.9 | M | 1.62857 | 16.2857 |
| 54 | MEM | 0.9 | E | 0.2860731 | 11.9197125 |
| 55 | MP | 1 | M | 0 | 0 |
| 56 | MRS | 1 | M | 0.193515 | 1.93515 |
| 57 | NAD | 1 | C | 1444 | 2245.4 |
| 58 | NADM | 1 | M | 140 | 1400 |
| 59 | NADV | 1 | V | 276 | 3368.3 |
| 60 | NAH | 1 | C | 477 | 741.5 |
| 61 | NAHM | 1 | M | 111 | 1112 |
| 62 | NAHV | 1 | V | 65 | 792.68293 |
| 63 | NUC | 0.9 | C | 0.996805 | 1.550241 |
| 64 | NUCM | 1 | C | 30 | 46.6563 |
| 65 | NUCMN | 1 | N | 6.7 | 44.37086 |
| 66 | O2 | 1 | C | 50 | 77.8 |
| 67 | O2M | 1 | M | 0.06 | 0.6 |
| 68 | O2V | 1 | V | 4.2 | 52 |
| 69 | PL | 1 | E | 19,900 | 829190.6748 |
| 70 | POL | 0.9 | N | 0.0931609 | 0.6169596 |
| 71 | PRO | 1 | C | 0.1036279 | 0.161163 |
| 72 | RIB | 0.9 | C | 0.2271681 | 0.353294 |
| 73 | ROS | 1 | C | 3.4 | 5.287714 |
| 74 | ROSE | 1 | E | 0.12 | 5.00 |
| 75 | ROSM | 1 | M | 3.0 | 30 |
| 76 | ROSN | 1 | N | 0.76 | 5.03311258 |
| 77 | TCA | 0.9 | M | 3.712047 | 37.12047 |
| 78 | TCAM | 1 | M | 399 | 3990 |
| 79 | TCAMC | 1 | C | 1710.9 | 2660.8 |
| 80 | YAP | 0.5 | N | 0.01204 | 0.0797351 |

**Table S4: Iron-Associated Proteins and Protein Groups:** All 5858 proteins in *S. cerevisiae* (37) were technically included in the model, but only 142 were named and organized into 22 groups. Each group was designated by 3 capitalized alphanumeric symbols typically reflecting their most prominent member. The 142 proteins listed below were obtained from (Lindahl and Vali 2022). “Copies per cell” (CPC) were from Ho et al (2018), and were converted into cellular concentrations (in µM) using the relationship

$$[\text{P}_{\text{i}}]_{fer} (\text{in μM})=\frac{copies}{cell}\frac{mole}{6.022\times10^{23}molecules}\frac{cell}{42\times10^{-15}L}\frac{10^{6}\mu M}{M}$$

Published data were typically obtained using cells grown under fermenting conditions, indicated by *fer* in table entries. For cells grown under respiring conditions, as modeled here, concentrations of group proteins in mitochondria were presumed to be 3× higher (indicated by *res*). Resulting member and group concentrations, *[P_i_]_res_* and *[P_g_]_res_* respectively, were tabulated. Certain group proteins were presumed to have different forms; percentages of each form are indicated.

The remaining 5716 unnamed and iron-free proteins in the cell were treated as a single protein group (PRO) that did not contain iron and was not involved in any reaction apart from its own biosynthesis. Each member of PRO was assumed to be 500 amino acid residues long, the approximate average length of proteins in yeast (Saccharomyces Genome Database). The total length of PRO was assumed to be approximately 5716 times the average length. There are 2621 copies (mean abundance) of the average protein per cell in yeast, implying that PRO should have a cellular concentration of 0.1036 µM. PRO was located in cytosol.

| Group Protein (location) | Member Protein  (location) | Identifier | Copies per cell | $[P_{i}]_{fer}$  (cellular) | $\frac{[P_{i}]_{fer}}{[P_{g}]_{fer}}=\text{wt}$ | $[P_{i}]_{res}$  (cellular) | $\frac{[P_{i}]_{res}}{[P_{g}]_{res}}=\text{wtr}$ |
| --- | --- | --- | --- | --- | --- | --- | --- |
| **1. AFT (c,n)** | Aft1(c, n) | YGL071W | 2568.5 | 0.10153 | 1.54866 | - | - |
|  | Aft2 (c, n) | YPL202C | 261.9 | 0.01036 | 0.15802 | - | - |
|  | Cth1(c) | YDR151C | 1729.5 | 0.06838 | 1.04301 | - | - |
|  | Cth2 (c) | YLR136C | 2073.6 | 0.08198 | 1.25046 | - | - |
|  | n = 4 |  |  | [AFT]_ave_ = 0.06556 |  |  |  |
|  |  |  | Forms (50%:50%) | [AFT] = 0.03278  [aAFT] = 0.03278 |  |  |  |
|  |  |  |  |  |  |  |  |
| **2. ATM (m)** | Atm1 (m) | YMR301C | 2223.4 | 0.08791 | 1.4782 | 3× = 0.2637 | 1.4782 |
|  | Mmt1 (m) | YMR177W | 1491.9 | 0.05898 | 0.99176 | 3× = 0.17694 | 0.99176 |
|  | Mmt2 (m) | YPL224C | 797.1 | 0.03151 | 0.52985 | 3× = 0.09453 | 0.52985 |
|  | n = 3 |  |  | [ATM]_ave_ = 0.05947 |  | [ATM]_ave_ = 0.17841 |  |
|  |  |  | Forms: | (none) |  |  |  |
|  |  |  |  |  |  |  |  |
| **3. CAT (c)** | Aim32 (m) | YML050W | 1175 | 0.046556 | 0.110888 | - | - |
|  | Ccp1 (c) | YKR066C | 8440.8 | 0.33372 | 0.794863 | - | - |
|  | Cta1(peroxisomes) | YDR256C | 1241.8 | 0.049097 | 0.11694 | - | - |
|  | Ctt1 (c) | YGR088W | 5783.2 | 0.22865 | 0.544604 | - | - |
|  | Grx6 (golgi) | YDL010W | 2629.0 | 0.10394 | 0.247567 | - | - |
|  | Yhb1 (c) | YGR234W | 44442.3 | 1.75711 | 4.18513 | - | - |
|  | Jlp1 (Guest) | YLL057C | 573 | 0.022655 | --- |  |  |
|  | n = 6 |  |  | [CAT]_ave_ = 0.45643 |  |  |  |
|  |  |  | Forms (10:90): | [aCAT] = 0.045643  [CAT] = 0.410787 |  |  |  |
|  |  |  |  |  |  |  |  |
| **4. CCC (v)** | Ccc1 (v) | YLR220W | 3623.9 | 0.14328 | 1.000 | - | - |
|  | n = 1 |  |  | [CCC]_ave_ = 0.14328 |  |  |  |
|  |  |  | Forms: | (none) |  |  |  |
|  |  |  |  |  |  |  |  |
| **5. CIA (c)** | Cfd1 (c) | YIL003W | 1515.1 | 0.05990 | 0.384872 | - | - |
|  | Cia1 (c) | YDR267C | 2906.6 | 0.11492 | 0.73839 | - | - |
|  | Cia2 (c) | YHR122W | 3033.4 | 0.11993 | 0.77058 | - | - |
|  | Met18 (c) | YIL128W | 3235 | 0.127904 | 0.821815 | - | - |
|  | Dre2 (c) | YKR071C | 4827.2 | 0.19085 | 1.226259 | - | - |
|  | Mms19 (c) was discovered to be identical to Met18 post-analysis | YIL128W | 3235.7 | 0.12793 | 0.821982 | - | - |
|  | Nar1 (c) | YNL240C | 1419.0 | 0.05610 | 0.360456 | - | - |
|  | Nbp35 (c) | YGL091C | 8348.8 | 0.33009 | 2.12091 | - | - |
|  | Tah18 (c) |  | 6907.6 | 0.27310 | 1.754735 | - | - |
|  | n = 9 |  |  | [CIA]_ave_ = 0.155636 |  |  |  |
|  |  |  | Forms:  (10:45:45) | [aaCIA] = 0.01556  [aCIA] = 0.0700362  [CIA] = 0.0700362 |  |  |  |
|  |  |  |  |  |  |  |  |
| **6. ETC (m)** | Cob1 (m) | Q0105 | 8283 | 0.374897 | 1.77286 | 9× = 3.37407 | 2.329058 |
|  | Coq6 (m) | YGR255C | 4718 | 0.186538 | 0.882123 | 3× = 0.55961 | 0.3862885 |
|  | Coq7 (m) | YOR125C | 2646 | 0.104616 | 0.494720 | 3× = 0.313848 | 0.2166435 |
|  | Cox1 (m) | Q0045 | 2382 | 0.094178 | 0.445360 | 9× = 0.847606 | 0.585087 |
|  | Cox10 (m) | YPL172C | 734 | 0.029020 | 0.1372332 | 3× = 0.08706 | 0.060096 |
|  | Cox15 (m) | YER141W | 5872 | 0.2321646 | 1.0978874 | 3× = 0.696494 | 0.480777 |
|  | Cyb2 (m) | YML054C | 6968 | 0.275498 | 1.3028076 | 9× = 2.47948 | 1.711539 |
|  | Cyc1(m) | YJR048W | 9225 | 0.364734 | 1.724797 | 9× = 3.28266 | 2.265988 |
|  | Cyc2(m) | YOR037W | 1319 | 0.052150 | 0.246613 | 3× = 0.15645 | 0.1079945 |
|  | Cyc7(m) | YEL039C | 3192 | 0.126204 | 0.596808 | 3× = 0.37861 | 0.261347 |
|  | Cyt1 (m) | YOR065W | 6247 | 0.246991 | 1.168000 | 9× = 2.22291 | 1.534434 |
|  | Mss51(m) | YLR203C | 6426 | 0.2540684 | 1.201469 | 3× = 0.762205 | 0.526136 |
|  | Rip1(m) | YEL024W | 10319 | 0.407988 | 1.9293419 | 9× = 3.67189 | 2.534638 |
|  | Cir2 (guest) | YOR356W | 4764 | 0.188350 | --- | --- | --- |
|  | n = 13 |  |  | [ETC]_ave_ = 0.211465 |  | [ETC]_ave_ = 1.44868 |  |
|  |  |  | Forms:  (10:90) | [aETC] = 0.021146  [ETC] = 0.190318 |  | [aETC] = 0.144868  [ETC] = 1.303812 |  |
|  |  |  |  |  |  |  |  |
| **7. FT3 (c)** | Fet3 (c) | YMR058W | 8058.8 | 0.31862 | 3.44994 | - | - |
|  | Fet4 (c) | YMR319C | 808.2 | 0.031953 | 0.345979 | - | - |
|  | Fre1(c) | YLR214W | 2143.5 | 0.084747 | 0.091762 | - | - |
|  | Fre2(c) | YKL220c | (1277) | 0.050488 | 0.546672 | - | - |
|  | Fre3(c) | YOR381w | (1277) | 0.050488 | 0.546672 | - | - |
|  | Fre4(c) | YNR060w | 1141.0 | 0.04511 | 0.488441 | - | - |
|  | Fre5 (m) | YOR384w | 2212.0 | 0.0874558 | 0.946951 | - | - |
|  | Fre7 (c) | YOL152w | 120.3 | 0.0047563 | 0.051500 | - | - |
|  | Ftr1 (c) | YER145C | 6019.5 | 0.237993 | 2.576933 | - | - |
|  | Smf1 (c) | YOL122C | 302.0 | 0.011940 | 0.129283 | - | - |
|  | n = 10 |  |  | [FT3]_ave_ = 0.092355 |  |  |  |
|  |  |  | Forms:  (50:50) | [aFT3] = 0.046177  [FT3] = 0.046177 |  |  |  |
|  |  |  |  |  |  |  |  |
| **8. FT5 (v)** | Fet5 (v) | YFL041W | 4051.2 | 0.16017 | 1.2311 | - | - |
|  | Fre6 (v) | YLL051C | 2071.3 | 0.08189 | 0.62944 | - | - |
|  | Fth1 (v) | YBR207W | 4175.1 | 0.16507 | 1.26879 | - | - |
|  | Smf3 (v) | YLR034C | 2865.2 | 0.11328 | 0.87071 | - | - |
|  | n = 4 |  |  | [FT5]_ave_ = 0.1301 |  |  |  |
|  |  |  | Forms  (50:50) | [aFT5] = 0.06505  [FT] = 0.06505 |  |  |  |
|  |  |  |  |  |  |  |  |
| **9. GRX (c)** | Bol2 (c) | YGL220W | 3262.4 | 0.12898 | 0.644423 | - | - |
|  | Grx3 (c) | YDR098C | 7999.3 | 0.31627 | 1.580181 | - | - |
|  | Grx4 (c) | YER174C | 4259.6 | 0.16841 | 0.841427 | - | - |
|  | Apd1 (c, n) | YBR151W | 4728 | 0.186933 | 0.933974 | - | - |
|  | n = 4 |  |  | [GRX]_ave_ = 0.200148 |  |  |  |
|  |  |  | Forms  (50:50) | [aGRX] = 0.10007  [GRX] = 0.10007 |  |  |  |
|  |  |  |  |  |  |  |  |
| **10. HEM (m, n)** | Hem15 | YOR176W | 8709.4 | 0.34434 | 2.043452 | 3×=1.03302 | 4.057519 |
|  | Hap1 | YLR256W | 1412.2 | 0.05583 | 0.331318 | 0.05583 | 0.21929 |
|  | Hap2 | YGL237C | 1656.9 | 0.06551 | 0.388763 | 0.06551 | 0.257312 |
|  | Hap3 | YBL021C | 1706.4 | 0.06746 | 0.400335 | 0.06746 | 0.264971 |
|  | Hap4 | YKL109W | 789.6 | 0.03122 | 0.185272 | 0.03122 | 0.122627 |
|  | Hap5 | YOR358W | 1435.3 | 0.05675 | 0.336777 | 0.05675 | 0.222904 |
|  | Rox1 | YPR065W | 1290.5 | 0.05102 | 0.302773 | 0.05102 | 0.200397 |
|  | Tdh3 (c) | YGR192C | 1011608 (×0.0169) | 0.67594 | 4.011299 | 0.67594 | 2.654972 |
|  | n = 8 |  |  | [HEM]_ave_ = 0.168509 |  | [HEM]_ave_ = 0.254594 |  |
|  |  |  | Forms  (50:50) | [aHEM] = 0.0842545  [HEM] = 0.0842545 |  | [aHEM] = 0.127297  [HEM] = 0.127297 |  |
|  |  |  |  |  |  |  |  |
| **11. HMX (e)** | Hmx1 | YLR205C | 3067 | 0.121262 | 1.000 | - | - |
|  | n = 1 |  |  | [HMX]_ave_ = 0.121262 |  |  |  |
|  |  |  | Forms  (10:90) | [aHMX] = 0.01213  [HMX] = 0.109136 |  |  |  |
|  |  |  |  |  |  |  |  |
| **12. ISA (m)** | Bol1 | YAL044W-A | 4236.0 | 0.16748 | 1.28043 | 3× = 0.50244 | 1.28043 |
|  | Bol3 | YAL046C | 2649.3 | 0.10473 | 0.800688 | 3× = 0.31419 | 0.800688 |
|  | Iba57 | YJR122W | 1140.9 | 0.045108 | 0.344862 | 3× = 0.135324 | 0.344862 |
|  | Isa1 | YLL027W | 1541.9 | 0.06097 | 0.466131 | 3× = 0.182910 | 0.466131 |
|  | Isa2 | YPR067W | 2619.7 | 0.10359 | 0.791972 | 3× = 0.310770 | 0.791972 |
|  | Nfu1 | YKL040C | 7661.7 | 0.30293 | 2.315978 | 3× = 0.90879 | 2.315978 |
|  | RSM22 (guest) | YKL155C | 2144 |  |  |  |  |
|  | n = 6 |  |  | [ISA]_ave_ = 0.13080 |  | [ISA_t_] = 0.3924 |  |
|  |  |  | Forms  (10:45:45) | [aaISA] = 0.01308  [aISA] = 0.05886  [ISA] = 0.05886 |  | [aaISA] = 0.03924  [aISA] = 0.17658  [ISA] = 0.17658 |  |
|  |  |  |  |  |  |  |  |
| **13. ISU (m)** | Acp1 | YKL192C | 21858.0 | 0.86421 | 3.08857 | 3× = 2.59263 | 3.08857 |
|  | Arh1 | YDR376W | 2224.7 | 0.087959 | 0.314353 | 3× = 0.263877 | 0.314353 |
|  | Grx5 | YPL059W | 7362.5 | 0.291095 | 1.0403346 | 3× = 0.873285 | 1.0403346 |
|  | Isd11 | YER048W-A | 7264.6 | 0.287225 | 1.0265038 | 3× = 0.84675 | 1.0265038 |
|  | Isu1 | YPL135W | 7702.1 | 0.304522 | 1.0883209 | 3× = 0.913566 | 1.0883209 |
|  | Isu2 | YOR226C | 4517.5 | 0.178611 | 0.6383319 | 3× = 0.535833 | 0.6383319 |
|  | Jac1 | YGL018C | 2725.9 | 0.107775 | 0.3851734 | 3× = 0.323325 | 0.3851734 |
|  | Mge1 | YOR232W | 13580.2 | 0.536928 | 1.9189090 | 3× = 1.610784 | 1.9189090 |
|  | Nfs1 | YCL017C | 7264.6 | 0.287225 | 1.0265038 | 3× = 0.861675 | 1.0265038 |
|  | Ssq1 | YLR369W | 3459.8 | 0.136792 | 0.4888763 | 3× = 0.410376 | 0.4888763 |
|  | Yah1 | YPL252C | 3599.1 | 0.1422997 | 0.5085601 | 3× = 0.426899 | 0.5085601 |
|  | Yfh1 | YDL120W | 3365.6 | 0.1330676 | 0.4755658 | 3× = 0.399203 | 0.4755658 |
|  | n = 12 |  |  | [ISA]_ave_ = 0.279809 |  | [ISU_t_] = 1.1772 |  |
|  |  |  | Forms  (10:45:45) | [aaISU] = 0.027981  [aISU] = 0.125914  [ISU] = 0.125914 |  | [aaISU] = 0.11772  [aISU] = 0.52974  [ISU] = 0.52974 |  |
|  |  |  |  |  |  |  |  |
| **14. LEU (c)** | Glt1 | YDL171C | 68365.2 | 2.70295 | 2.92658 | - | - |
|  | Leu1 | YGL009C | 14157.9 | 0.55976 | 0.606071 | - | - |
|  | Met5 | YJR137C | 2428.6 | 0.09602 | 0.103964 | - | - |
|  | Met8 | YBR213W | 8488.8 | 0.33562 | 0.363387 | - | - |
|  | Bna2 (guest) | YJR078W |  |  |  |  |  |
|  | n = 4 |  |  | [LEU]_ave_ = 0.92359 |  |  |  |
|  |  |  | Forms  (10:90) | [aLEU] = 0.09236  [LEU] = 0.83123 |  |  |  |
|  |  |  |  |  |  |  |  |
| **15. LYS (m)** | Ilv3 | YJR016C | 47063.7 | 1.86075 | 3.08492 | 3× = 5.58225 | 3.08492 |
|  | Lys4 | YDR234W | 10156.0 | 0.401538 | 0.665707 | 3× = 1.20461 | 0.665707 |
|  | Thi4 | YGR144W | 2611.3 | 0.103244 | 0.171167 | 3× = 0.309732 | 0.171167 |
|  | Thi5 | YFL058W | 1193 | 0.047168 | 0.078199 | 3× = 0.141504 | 0.078199 |
|  | Thi13 (guest) | YDL244W | 1344 | 0.053185 |  |  |  |
|  | Fmp12 (guest) | YHL021C | 10773 | 0.425947 |  |  |  |
|  | Thi11 (guest) | YJR156C | 752 | 0.029732 |  |  |  |
|  | Thi12 (guest) | YNL332W | 1262 | 0.049896 |  |  |  |
|  | n = 4 |  |  | [LYS]_ave_ = 0.603175 |  | [LYS_t_] = 1.809525 |  |
|  |  |  | Forms  (10:90) | [aLYS] = 0.060318  [LYS] = 0.54286 |  | [aLYS] = 0.180952  [LYS] = 1.62857 |  |
|  |  |  |  |  |  |  |  |
| **16. MEM (e)** | Cyb5 | YNL111C | 5939.1 | 0.23481 | 0.738726 | - | - |
|  | Cyp51 | YHR007C | 16771.8 | 0.66311 | 2.086183 | - | - |
|  | Dap1 | YPL170W | 5891.9 | 0.1882 | 0.592088 | - | - |
|  | Erg3 | YLR056W | 7626.3 | 0.301521 | 0.948603 | - | - |
|  | Erg5 | YMR015C | 8727.8 | 0.345071 | 1.085614 | - | - |
|  | Erg25 | YGR060W | 24158.1 | 0.955139 | 3.004924 | - | - |
|  | Fre8 | YLR047c | 770 | 0.0304439 | 0.095778 | - | - |
|  | Mpo1 | YGL010W | 2016.8 | 0.0797382 | 0.250861 | - | - |
|  | Ncp1 | YHR042W | 13007.3 | 0.514270 | 1.617924 | - | - |
|  | Ole1 | YGL055W | 9584.4 | 0.378938 | 1.192161 | - | - |
|  | Scs7 | YMR272C | 4169.2 | 0.16484 | 0.518596 | - | - |
|  | Sfh5 | YJL145W | 6822 | 0.2697 | 0.848492 | - | - |
|  | Sur2 | YDR297W | 7011.8 | 0.277225 | 0.872166 | - | - |
|  | Yno1 | YGL160w | 1189 | 0.0470102 | 0.147897 | - | - |
|  | Dit2 (guest) | YDR402C | --- |  |  |  |  |
|  | n = 14 |  |  | [MEM]_ave_ = 0.317858 |  |  |  |
|  |  |  | Forms  (10:90) | [aMEM] = 0.0317858  [MEM] = 0.2860731 |  |  |  |
|  |  |  |  |  |  |  |  |
| **17. MRS (m)** | Mrs3 | YJL133W | 1631.5 | 0.064505 | 1.001879 | 3× = 0.193515 | 1.001879 |
|  | Mrs4 | YKR052C | 1411.2 | 0.055795 | 0.8665976 | 3× = 0.167385 | 0.8665976 |
|  | Rim2 | YBR192W | 1842.6 | 0.072852 | 1.131524 | 3× = 0.218556 | 1.131524 |
|  | n = 3 |  | No forms | [MRS]_ave_ = 0.064384 |  | [MRS]_ave_ = 0.19315 |  |
|  |  |  |  |  |  |  |  |
| **18. NUC (c)** | Bna1 | YJR025C | 7560.7 | 0.298904 | 0.2698758 | - | - |
|  | Rnr2 | YJL026W | 31412.2 | 1.241962 | 1.1213483 | - | - |
|  | Rnr4 | YGR180C | 84772.8 | 3.351710 | 3.0262073 | - | - |
|  | Tyw1 | YPL207W | 4385.2 | 0.1694254 | 0.1529716 | - | - |
|  | n = 5 |  |  | [NUC]_ave_ = 1.107451  Corrected but not used: 1.2655 |  |  |  |
|  |  |  | Forms  (10:90) | [aNUC] = 0.110745  [NUC] = 0.996805  Corrected but not used  [aNUC] = 0.126  [NUC] = 1.139 |  |  |  |
|  |  |  |  |  |  |  |  |
| **19. POL (n)** | Chl1 | YPL008W | 394.9 | 0.015613 | 0.014336 | - | - |
|  | Dna2 | YHR164C | 1836.1 | 0.0725949 | 0.733248 | - | - |
|  | Ntg2 | YOL043C | 89.8 | 0.00355047 | 0.035862 | - | - |
|  | Pol1 | YNL102W | 2551.0 | 0.1008603 | 1.018744 | - | - |
|  | Pol2 | YNL262W | 3194.9 | 0.1263185 | 1.275885 | - | - |
|  | Pol3 | YDL102W | 3200.1 | 0.1265242 | 1.277963 | - | - |
|  | Pri2 | YKL045W | 2413.0 | 0.09540415 | 0.963634 | - | - |
|  | Rad3 | YER171W | 2892.4 | 0.11435846 | 1.155082 | - | - |
|  | Rev3 | YPL167C | 1453.5 | 0.05746785 | 0.580456 | - | - |
|  | Tpa1 | YER049W | 8155.0 | 0.32242887 | 3.256706 | - | - |
|  | Exo5 | YBR163w | 1723 | 0.068123231 | 0.688081 | - | - |
|  | Jhd1 (guest) | YER051W | 471 | 0.0186207 |  |  |  |
|  | Jhd2 (guest) | YJR119C | 1113 | 0.0440222 |  |  |  |
|  | n = 11 |  |  | [POL]_ave_ = 0.0990046 |  |  |  |
|  |  |  | Forms  (10:90) | [aPOL] = 0.0103512  [POL] = 0.0931609 |  |  |  |
|  |  |  |  |  |  |  |  |
| **20. PRO** | P_1_ |  | 2621 | 0.1036279 | 1.00 | - | - |
|  | P_2_ |  | 2621 | 0.1036279 | 1.00 | - | - |
|  | P_3_ … |  | 2621 | 0.1036279 | 1.00 | - | - |
|  | P_5716_ |  | 2621 | 0.1036279 | 1.00 | - | - |
|  | n = 5716 |  | No Forms | [PRO]_ave_ = 0.1036279 |  |  |  |
|  |  |  |  |  |  |  |  |
| **21. RIB (c)** | Cbr1 | YIL043C | 9374.8 | 0.37065679 | 1.468477 | - | - |
|  | Dbr1 | YKL149C | 1545 | 0.0610855 | 0.24201 | - | - |
|  | Dph1 | YIL103W | 2897.9 | 0.11457592 | 0.45393 | - | - |
|  | Dph2 | YKL191W | 3518.5 | 0.13911293 | 0.551141 | - | - |
|  | Dph3 | YBL071W-A | 8829.2 | 0.34908510 | 1.383014 | - | - |
|  | Dph4 | YJR097W | 1975.2 | 0.07809460 | 0.309397 | - | - |
|  | Elp3 | YPL086C | 4680.2 | 0.18504373 | 0.733111 | - | - |
|  | Lto1 | YNL260C | 1001.8 | 0.03960874 | 0.156923 | - | - |
|  | Rli1 | YDR091C | 16172.9 | 0.63943714 | 2.533337 | - | - |
|  | Yae1 | YJR067C | 2781.0 | 0.10995398 | 0.435618 | - | - |
|  | Lia1 | YJR070C | 21185 | 0.83760339 | 3.318437 | - | - |
|  | Ncs6 | YGL211W | 2647 | 0.10465594 | 1.468477 | - | - |
|  | Ate1 (guest) | YGL017W |  |  |  |  |  |
|  | n = 12 |  |  | [RIB]_ave_ = 0.252409 |  |  |  |
|  |  |  | Forms  (10:90) | [aRIB] = 0.025241  [RIB] = 0.2271681 |  |  |  |
|  |  |  |  |  |  |  |  |
| **22. TCA (m)** | Aco1 | YLR304C | 56806.8 | 2.24600275 | 4.0969256 | 9× = 20.214024 | 4.900968 |
|  | Aco2 | YJL200C | 15861.5 | 0.62712514 | 1.1439367 | 3× = 1.8813754 | 0.4561467 |
|  | Bio2 | YGR286C | 4153.8 | 0.16423115 | 0.2995734 | 3× = 0.4926034 | 0.11943358 |
|  | Lip5 | YOR196C | 3869.5 | 0.15299062 | 0.27906964 | 3× = 0.4589719 | 0.11127949 |
|  | Sdh2 | YLL041C | 8534.9 | 0.33744919 | 0.61553986 | 9× = 3.0370427 | 0.73634266 |
|  | Sdh3 | YKL141W | 4718.8 | 0.18656988 | 0.34032145 | 9× = 1.6791289 | 0.40711124 |
|  | Sdh4 | YDR178W | 3114.7 | 0.12314766 | 0.22463321 | 9× = 1.1083289 | 0.26871859 |
|  | Shh3 (guest) | YMR118C | --- |  |  |  |  |
|  | Shh4 (guest) | YLR164W | --- |  |  |  |  |
|  | n = 7 |  |  | [TCA_t_] = 0.5482166 |  | [TCA_t_] = 4.124496 |  |
|  |  |  | Forms  (10:90) | [aTCA] = 0.0548216  [TAC] = 0.4933949 |  | [aTCA] = 0.412449  [TAC] = 3.712047 |  |
|  |  |  |  |  |  |  |  |
| **23. YAP (n)** | Yap5 | YIR018W | 609.2 | 0.02408 | 1.00 | - | - |
|  | Yap7 (guest) | YOL028C | 1591 | 0.0628929 |  |  |  |
|  | n = 1 |  | Forms (50:50) | [YAP] = 0.01204  [aYAP] = 0.01204 |  |  |  |

**Table S5 Iron content of groups based on iron-content of members.** F4, F3, F2, HE, FF, and FO, refer to [Fe_4_S_4_], [Fe_3_S_4_], [Fe_2_S_2_], heme, Fe-O-Fe diiron centers, and mononuclear Fe complexes with O/N ligands. C_i_ refers to the stoichiometric coefficient (number of centers per monomer member protein). Subscript W refers to the effective contribution of the center within the protein group.

| Group | Member | C_i(F4)_ | C_i(F4)w_ | C_i(F3)_ | C_i(F3)w_ | C_i(F2)_ | C_i(F2)w_ | C_i(HE)_ | C_i(HE)w_ | C_i(FF)_ | C_i(FF)w_ | C_i(FO)_ | C_i(FO)w_ |
| --- | --- | --- | --- | --- | --- | --- | --- | --- | --- | --- | --- | --- | --- |
| **1. AFT** | Aft1 | 0 | 0 | 0 | 0 | 0.5 | 0.77433 | 0 | 0 | 0 | 0 | 0 | 0 |
|  | Aft2 | 0 | 0 | 0 | 0 | 0.5 | 0.07901 | 0 | 0 | 0 | 0 | 0 | 0 |
|  | Cth1 | 0 | 0 | 0 | 0 | 0 | 0 | 0 | 0 | 0 | 0 | 0 | 0 |
|  | Cth2 | 0 | 0 | 0 | 0 | 0 | 0 | 0 | 0 | 0 | 0 | 0 | 0 |
| Group Coefficients |  | NA | 0 | NA | 0 | NA | 0.85334 | NA | 0 | NA | 0 | NA | 0 |
|  |  |  |  |  |  |  |  |  |  |  |  |  |  |
| **2. ATM** | Atm1 | 0 | 0 | 0 | 0 | 0 | 0 | 0 | 0 | 0 | 0 | 0 | 0 |
|  | Mmt1 | 0 | 0 | 0 | 0 | 0 | 0 | 0 | 0 | 0 | 0 | 0 | 0 |
|  | Mmt2 | 0 | 0 | 0 | 0 | 0 | 0 | 0 | 0 | 0 | 0 | 0 | 0 |
| Group Coefficients |  | NA | 0 | NA | 0 | NA | 0 | NA | 0 | NA | 0 | NA | 0 |
|  |  |  |  |  |  |  |  |  |  |  |  |  |  |
| **3. CAT** | Aim32 | 0 | 0 | 0 | 0 | 1 | 0.110888 | 0 | 0 | 0 | 0 | 0 | 0 |
|  | Ccp1 | 0 | 0 | 0 | 0 | 0 | 0 | 2 | 1.589728 | 0 | 0 | 0 | 0 |
|  | Cta1 | 0 | 0 | 0 | 0 | 0 | 0 | 1 | 0.116940 | 0 | 0 | 0 | 0 |
|  | Ctt1 | 0 | 0 | 0 | 0 | 0 | 0 | 1 | 0.544604 | 0 | 0 | 0 | 0 |
|  | Grx6 | 0 | 0 | 0 | 0 | 0.5 | 0.123784 | 0 | 0 | 0 | 0 | 0 | 0 |
|  | Yhb1 | 0 | 0 | 0 | 0 | 0 | 0 | 1 | 4.18513 | 0 | 0 | 0 | 0 |
|  |  |  |  |  |  |  |  |  |  |  |  |  |  |
| Group Coefficients |  | NA | 0 | NA | 0 | NA | 0.234672 | NA | 6.436402 | NA | 0 | NA | 0 |
|  |  |  |  |  |  |  |  |  |  |  |  |  |  |
| **4. CCC** | Ccc1 | 0 | 0 | 0 | 0 | 0 | 0 | 0 | 0 | 0 | 0 | 0 | 0 |
| Group Coefficients |  | NA | 0 | NA | 0 | NA | 0 | NA | 0 | NA | 0 | NA | 0 |
|  |  |  |  |  |  |  |  |  |  |  |  |  |  |
| **5. CIA** | Cfd1 | 0.5 | 0.192436 | 0 | 0 | 0 | 0 | 0 | 0 | 0 | 0 | 0 | 0 |
|  | Cia1 | 0 | 0 | 0 | 0 | 0 | 0 | 0 | 0 | 0 | 0 | 0 | 0 |
|  | Cia2 | 0 | 0 | 0 | 0 | 0 | 0 | 0 | 0 | 0 | 0 | 0 | 0 |
|  | Met18 | 0 | 0 | 0 | 0 | 0 | 0 | 0 | 0 | 0 | 0 | 0 | 0 |
|  | Dre2 | 0.5 | 0.613129 | 0 | 0 | 1.5 | 1.839388 | 0 | 0 | 0 | 0 | 0 | 0 |
|  | Mms19 | 0 | 0 | 0 | 0 | 0 | 0 | 0 | 0 | 0 | 0 | 0 | 0 |
|  | Nar1 | 2 | 0.720912 | 0 | 0 | 0 | 0 | 0 | 0 | 0 | 0 | 0 | 0 |
|  | Nbp35 | 1.5 | 3.181365 | 0 | 0 | 0 | 0 | 0 | 0 | 0 | 0 | 0 | 0 |
|  | Tah18 | 0 | 0 | 0 | 0 | 0 | 0 | 0 | 0 | 0 | 0 | 0 | 0 |
| Group Coefficients |  | NA | 4.707842 | NA | 0 | NA | 1.839388 | NA | 0 | NA | 0 | NA | 0 |
|  |  |  |  |  |  |  |  |  |  |  |  |  |  |
| **6. ETC** | Cob1 | 0 | 0 | 0 | 0 | 0 | 0 | 2 | 3.54572 | 0 | 0 | 0 | 0 |
|  | Coq6 | 0 | 0 | 0 | 0 | 0 | 0 | 0 | 0 | 0 | 0 | 0 | 0 |
|  | Coq7 | 0 | 0 | 0 | 0 | 0 | 0 | 0 | 0 | 1 | 0.494720 | 0 | 0 |
|  | Cox1 | 0 | 0 | 0 | 0 | 0 | 0 | 2 | 0.89072 | 0 | 0 | 0 | 0 |
|  | Cox10 | 0 | 0 | 0 | 0 | 0 | 0 | 1 | 0.137233 | 0 | 0 | 0 | 0 |
|  | Cox15 | 0 | 0 | 0 | 0 | 0 | 0 | 2 | 2.195775 | 0 | 0 | 0 | 0 |
|  | Cyb2 | 0 | 0 | 0 | 0 | 0 | 0 | 1 | 1.302808 | 0 | 0 | 0 | 0 |
|  | Cyc1 | 0 | 0 | 0 | 0 | 0 | 0 | 1 | 1.724797 | 0 | 0 | 0 | 0 |
|  | Cyc2 | 0 | 0 | 0 | 0 | 0 | 0 | 0 | 0 | 0 | 0 | 0 | 0 |
|  | Cyc7 | 0 | 0 | 0 | 0 | 0 | 0 | 1 | 0.596808 | 0 | 0 | 0 | 0 |
|  | Cyt1 | 0 | 0 | 0 | 0 | 0 | 0 | 1 | 1.168000 | 0 | 0 | 0 | 0 |
|  | Mss51 | 0 | 0 | 0 | 0 | 0 | 0 | 1 | 1.201469 | 0 | 0 | 0 | 0 |
|  | Rip1 | 0 | 0 | 0 | 0 | 1 | 1.929342 | 0 | 0 | 0 | 0 | 0 | 0 |
| Group Coefficients (respiring) |  | NA | 0 | NA | 0 | NA | 2.534638 | NA | 13.14938 | NA | 0.216644 | 0 | 0 |
|  |  |  |  |  |  |  |  |  |  |  |  |  |  |
| **7. FT3** | Fet3 | 0 | 0 | 0 | 0 | 0 | 0 | 0 | 0 | 0 | 0 | 0 | 0 |
|  | Fet4 | 0 | 0 | 0 | 0 | 0 | 0 | 0 | 0 | 0 | 0 | 0 | 0 |
|  | Fre1 | 0 | 0 | 0 | 0 | 0 | 0 | 2 | 0.183524 | 0 | 0 | 0 | 0 |
|  | Fre2 | 0 | 0 | 0 | 0 | 0 | 0 | 1 | 0.546672 | 0 | 0 | 0 | 0 |
|  | Fre3 | 0 | 0 | 0 | 0 | 0 | 0 | 1 | 0.546672 | 0 | 0 | 0 | 0 |
|  | Fre4 | 0 | 0 | 0 | 0 | 0 | 0 | 1 | 0.488441 | 0 | 0 | 0 | 0 |
|  | Fre5 | 0 | 0 | 0 | 0 | 0 | 0 | 1 | 0.946951 | 0 | 0 | 0 | 0 |
|  | Fre7 | 0 | 0 | 0 | 0 | 0 | 0 | 1 | 0.0515 | 0 | 0 | 0 | 0 |
|  | Ftr1 | 0 | 0 | 0 | 0 | 0 | 0 | 0 | 0 | 0 | 0 | 0 | 0 |
|  | Smf1 | 0 | 0 | 0 | 0 | 0 | 0 | 0 | 0 | 0 | 0 | 0 | 0 |
| Group Coefficients |  | NA | 0 | NA | 0 | NA | 0 | NA | 2.76376 | NA | 0 | NA | 0 |
|  |  |  |  |  |  |  |  |  |  |  |  |  |  |
| **8. FT5** | Fet5 | 0 | 0 | 0 | 0 | 0 | 0 | 0 | 0 | 0 | 0 | 0 | 0 |
|  | Fre6 | 0 | 0 | 0 | 0 | 0 | 0 | 2 | 1.25888 | 0 | 0 | 0 | 0 |
|  | Fth1 | 0 | 0 | 0 | 0 | 0 | 0 | 0 | 0 | 0 | 0 | 0 | 0 |
|  | Smf3 | 0 | 0 | 0 | 0 | 0 | 0 | 0 | 0 | 0 | 0 | 0 | 0 |
| Group Coefficients |  | NA | 0 | NA | 0 | NA | 0 | NA | 1.25888 | NA | 0 | NA | 0 |
|  |  |  |  |  |  |  |  |  |  |  |  |  |  |
| **9. GRX** | Bol2 | 0 | 0 | 0 | 0 | 0.5 | 0.322212 | 0 | 0 | 0 | 0 | 0 | 0 |
|  | Grx3 | 0 | 0 | 0 | 0 | 0.5 | 0.790090 | 0 | 0 | 0 | 0 | 0 | 0 |
|  | Grx4 | 0 | 0 | 0 | 0 | 0.5 | 0.420714 | 0 | 0 | 0 | 0 | 0 | 0 |
|  | Apd1 | 0 | 0 | 0 | 0 | 1.0 | 0.933974 | 0 | 0 | 0 | 0 | 0 | 0 |
| Group Coefficients |  | NA | 0 | NA | 0 | NA | 2.466990 | NA | 0 | NA | 0 | NA | 0 |
|  |  |  |  |  |  |  |  |  |  |  |  |  |  |
| **10. HEM** | Hem15 | 0 | 0 | 0 | 0 | 0 | 0 | 1 | 4.057519 | 0 | 0 | 0 | 0 |
|  | Hap1 | 0 | 0 | 0 | 0 | 0 | 0 | 1 | 0.21929 | 0 | 0 | 0 | 0 |
|  | Hap2 | 0 | 0 | 0 | 0 | 0 | 0 | 0 | 0 | 0 | 0 | 0 | 0 |
|  | Hap3 | 0 | 0 | 0 | 0 | 0 | 0 | 0 | 0 | 0 | 0 | 0 | 0 |
|  | Hap4 | 0 | 0 | 0 | 0 | 0 | 0 | 1 | 0.122627 | 0 | 0 | 0 | 0 |
|  | Hap5 | 0 | 0 | 0 | 0 | 0 | 0 | 0 | 0 | 0 | 0 | 0 | 0 |
|  | Rox1 | 0 | 0 | 0 | 0 | 0 | 0 | 0 | 0 | 0 | 0 | 0 | 0 |
|  | Tdh3 | 0 | 0 | 0 | 0 | 0 | 0 | “1” | 2.654972 | 0 | 0 | 0 | 0 |
| Group Coefficients  respiring |  | NA | 0 | NA | 0 | NA | 0 | NA | 7.054408 | NA | 0 | NA | 0 |
|  |  |  |  |  |  |  |  |  |  |  |  |  |  |
| **11. HMX** | Hmx1 | 0 | 0 | 0 | 0 | 0 | 0 | 1 | 1.00 | 0 | 0 | 0 | 0 |
|  |  | NA | 0 | NA | 0 | NA | 0 | NA | 1.00 | NA | 0 | NA | 0 |
|  |  |  |  |  |  |  |  |  |  |  |  |  |  |
| **12. ISA** | Bol1 | 0 | 0 | 0 | 0 | 0.5 | 0.640215 | 0 | 0 | 0 | 0 | 0 | 0 |
|  | Bol3 | 0 | 0 | 0 | 0 | 0.5 | 0.400344 | 0 | 0 | 0 | 0 | 0 | 0 |
|  | Iba57 | 0 | 0 | 0 | 0 | 0 | 0 | 0 | 0 | 0 | 0 | 0 | 0 |
|  | Isa1 | 0.5 | 0.233065 | 0 | 0 | 0.5 | 0.233065 | 0 | 0 | 0 | 0 | 0 | 0 |
|  | Isa2 | 0.5 | 0.395986 | 0 | 0 | 0.5 | 0.395986 | 0 | 0 | 0 | 0 | 0 | 0 |
|  | Nfu1 | 0.5 | 1.157989 | 0 | 0 | 0 | 0 | 0 | 0 | 0 | 0 | 0 | 0 |
| Group Coefficients  respiring |  | NA | 1.787040 | NA | 0 | NA | 1.669610 | NA | 0 | NA | 0 | NA | 0 |
|  |  |  |  |  |  |  |  |  |  |  |  |  |  |
| **13. ISU** | Acp1 | 0 | 0 | 0 | 0 | 0 | 0 | 0 | 0 | 0 | 0 | 0 | 0 |
|  | Arh1 | 0 | 0 | 0 | 0 | 0 | 0 | 0 | 0 | 0 | 0 | 0 | 0 |
|  | Grx5 | 0.5 | 0.521673 | 0 | 0 | 1.5 | 1.560502 | 0 | 0 | 0 | 0 | 0 | 0 |
|  | Isd11 | 0 | 0 | 0 | 0 | 0 | 0 | 0 | 0 | 0 | 0 | 0 | 0 |
|  | Isu1 | 0 | 0 | 0 | 0 | 1 | 1.088321 | 0 | 0 | 0 | 0 | 0 | 0 |
|  | Isu2 | 0 | 0 | 0 | 0 | 1 | 0.638332 | 0 | 0 | 0 | 0 | 0 | 0 |
|  | Jac1 | 0 | 0 | 0 | 0 | 0 | 0 | 0 | 0 | 0 | 0 | 0 | 0 |
|  | Mge1 | 0 | 0 | 0 | 0 | 0 | 0 | 0 | 0 | 0 | 0 | 0 | 0 |
|  | Nfs1 | 0 | 0 | 0 | 0 | 0 | 0 | 0 | 0 | 0 | 0 | 0 | 0 |
|  | Ssq1 | 0 | 0 | 0 | 0 | 0 | 0 | 0 | 0 | 0 | 0 | 0 | 0 |
|  | Yah1 | 0 | 0 | 0 | 0 | 1 | 0.508560 | 0 | 0 | 0 | 0 | 0 | 0 |
|  | Yfh1 | 0 | 0 | 0 | 0 | 0 | 0 | 0 | 0 | 0 | 0 | 1 | 0.475566 |
| Group Coefficients respiring |  | NA | 0.521673 | NA | 0 | NA | 3.795715 | NA | 0 | NA | 0 | NA | 0.475566 |
|  |  |  |  |  |  |  |  |  |  |  |  |  |  |
| **14. LEU** | Glt1 | 2 | 5.85316 | 1 | 2.92658 | 0 | 0 | 0 | 0 | 0 | 0 | 0 | 0 |
|  | Leu1 | 1 | 0.60607 | 0 | 0 | 0 | 0 | 0 | 0 | 0 | 0 | 0 | 0 |
|  | Met5 | 1 | 0.10396 | 0 | 0 | 0 | 0 | 1 | 0.103964 | 0 | 0 | 0 | 0 |
|  | Met8 | 0 | 0.36339 | 0 | 0 | 0 | 0 | 0 | 0 | 0 | 0 | 0 | 0 |
|  |  | NA | 6.92658 | NA | 2.92658 | NA | 0 | NA | 0.103964 | NA | 0 | NA | 0 |
|  |  |  |  |  |  |  |  |  |  |  |  |  |  |
| **15. LYS** | Ilv3 | 0 | 0 | 0 | 0 | 1 | 3.08492 | 0 | 0 | 0 | 0 | 0 | 0 |
|  | Lys4 | 1 | 0.665707 | 0 | 0 | 0 | 0 | 0 | 0 | 0 | 0 | 0 | 0 |
|  | Thi4 | 0 | 0 | 0 | 0 | 0 | 0 | 0 | 0 | 0 | 0 | 1 | 0.171167 |
|  | Thi5 | 0 | 0 | 0 | 0 | 0 | 0 | 0 | 0 | 0 | 0 | 1 | 0.078199 |
| Group Coefficients respiring |  | NA | 0.665707 | NA | 0 | NA | 3.08492 | NA | 0 | NA | 0 | NA | 0.249366 |
|  |  |  |  |  |  |  |  |  |  |  |  |  |  |
| **16. MEM** | Cyb5 | 0 | 0 | 0 | 0 | 0 | 0 | 1 | 0.738726 | 0 | 0 | 0 | 0 |
| corrected | Cyp51 | 0 | 0 | 0 | 0 | 0 | 0 | 1 | 2.086183 | 0 | 0 | 0 | 0 |
|  | Dap1 | 0 | 0 | 0 | 0 | 0 | 0 | 1 | 0.592088 | 0 | 0 | 0 | 0 |
|  | Erg3 | 0 | 0 | 0 | 0 | 0 | 0 | 0 | 0 | 1 | 0.948603 | 0 | 0 |
|  | Erg5 | 0 | 0 | 0 | 0 | 0 | 0 | 1 | 1.085614 | 0 | 0 | 0 | 0 |
|  | Erg25 | 0 | 0 | 0 | 0 | 0 | 0 | 0 | 0 | 1 | 3.004924 | 0 | 0 |
|  | Fre8 | 0 | 0 | 0 | 0 | 0 | 0 | 1 | 0.095778 | 0 | 0 | 0 | 0 |
|  | Mpo1 | 0 | 0 | 0 | 0 | 0 | 0 | 0 | 0 | 0 | 0 | 1 | 0.250861 |
|  | Ncp1 | 0 | 0 | 0 | 0 | 0 | 0 | 0 | 0 | 0 | 0 | 0 | 0 |
|  | Ole1 | 0 | 0 | 0 | 0 | 0 | 0 | 1 | 1.617924 | 1 | 1.617924 | 0 | 0 |
|  | Scs7 | 0 | 0 | 0 | 0 | 0 | 0 | 1 | 1.192161 | 1 | 1.192161 | 0 | 0 |
|  | Sfh5 | 0 | 0 | 0 | 0 | 0 | 0 | 1 | 0.518596 | 0 | 0 | 0 | 0 |
|  | Sur2 | 0 | 0 | 0 | 0 | 0 | 0 | 0 | 0 | 1 | 0.848492 | 0 | 0 |
|  | Yno1 | 0 | 0 | 0 | 0 | 0 | 0 | 2 | 0.295794 | 0 | 0 | 0 | 0 |
| Group Coefficients |  | NA | 0 | NA | 0 | NA | 0 | NA | 8.222864 | NA | 7.612104 | NA | 0.250861 |
|  |  |  |  |  |  |  |  |  |  |  |  |  |  |
| **17. MRS** | Mrs3 | 0 | 0 | 0 | 0 | 0 | 0 | 0 | 0 | 0 | 0 | 0 | 0 |
|  | Mrs4 | 0 | 0 | 0 | 0 | 0 | 0 | 0 | 0 | 0 | 0 | 0 | 0 |
|  | Rim2 | 0 | 0 | 0 | 0 | 0 | 0 | 0 | 0 | 0 | 0 | 0 | 0 |
| Group Coefficients |  | NA | 0 | NA | 0 | NA | 0 | NA | 0 | NA | 0 | NA | 0 |
|  |  |  |  |  |  |  |  |  |  |  |  |  |  |
| **18. NUC** | Bna1 | 0 | 0 | 0 | 0 | 0 | 0 | 0 | 0 | 0 | 0 | 1 | 0.269876 |
| (removed Ade4) | Rnr2 | 0 | 0 | 0 | 0 | 0 | 0 | 0 | 0 | 1 | 1.121348 | 0 | 0 |
|  | Rnr4 | 0 | 0 | 0 | 0 | 0 | 0 | 0 | 0 | 0 | 0 | 0 | 0 |
|  | Tyw1 | 2 | 0.305943 | 0 | 0 | 0 | 0 | 0 | 0 | 0 | 0 | 0 | 0 |
| Group Coefficients |  | NA | 0.756236  (Should be 0.305943) | NA | 0 | NA | 0 | NA | 0 | NA | 1.121348 | NA | 0.269876 |
|  |  |  |  |  |  |  |  |  |  |  |  |  |  |
| **19. POL** | Chl1 | 1 | 0.014336 | 0 | 0 | 0 | 0 | 0 | 0 | 0 | 0 | 0 | 0 |
|  | Dna2 | 1 | 0.733248 | 0 | 0 | 0 | 0 | 0 | 0 | 0 | 0 | 0 | 0 |
|  | Ntg2 | 1 | 0.035862 | 0 | 0 | 0 | 0 | 0 | 0 | 0 | 0 | 0 | 0 |
|  | Pol1 | 1 | 1.018744 | 0 | 0 | 0 | 0 | 0 | 0 | 0 | 0 | 0 | 0 |
|  | Pol2 | 1 | 1.275885 | 0 | 0 | 0 | 0 | 0 | 0 | 0 | 0 | 0 | 0 |
|  | Pol3 | 1 | 1.277963 | 0 | 0 | 0 | 0 | 0 | 0 | 0 | 0 | 0 | 0 |
|  | Pri2 | 1 | 0.963634 | 0 | 0 | 0 | 0 | 0 | 0 | 0 | 0 | 0 | 0 |
|  | Rad3 | 1 | 1.155082 | 0 | 0 | 0 | 0 | 0 | 0 | 0 | 0 | 0 | 0 |
|  | Rev3 | 1 | 0.580456 | 0 | 0 | 0 | 0 | 0 | 0 | 0 | 0 | 0 | 0 |
|  | Tpa1 | 0 | 0 | 0 | 0 | 0 | 0 | 0 | 0 | 0 | 0 | 1 | 3.256706 |
|  | Exo5 | 1 | 0.688081 | 0 | 0 | 0 | 0 | 0 | 0 | 0 | 0 | 0 | 0 |
| Group Coefficients |  | NA | 7.743291 | NA | 0 | NA | 0 | NA | 0 | NA | 0 | NA | 3.256706 |
|  |  |  |  |  |  |  |  |  |  |  |  |  |  |
| **20. PRO** | P1 | 0 | 0 | 0 | 0 | 0 | 0 | 0 | 0 | 0 | 0 | 0 | 0 |
|  | P2 | 0 | 0 | 0 | 0 | 0 | 0 | 0 | 0 | 0 | 0 | 0 | 0 |
|  | P3 etc | 0 | 0 | 0 | 0 | 0 | 0 | 0 | 0 | 0 | 0 | 0 | 0 |
| Group Coefficients |  | NA | 0 | NA | 0 | NA | 0 | NA | 0 | NA | 0 | NA | 0 |
|  |  |  |  |  |  |  |  |  |  |  |  |  |  |
| **21. RIB** | Cbr1 | 0 | 0 | 0 | 0 | 0 | 0 | 0 | 0 | 0 | 0 | 0 | 0 |
|  | Dbr1 | 0 | 0 | 0 | 0 | 0 | 0 | 0 | 0 | 0 | 0 | 1 | 0.24201 |
|  | Dph1 | 0.5 | 0.226965 | 0 | 0 | 0 | 0 | 0 | 0 | 0 | 0 | 0 | 0 |
|  | Dph2 | 0.5 | 0.275571 | 0 | 0 | 0 | 0 | 0 | 0 | 0 | 0 | 0 | 0 |
|  | Dph3 | 0 | 0 | 0 | 0 | 0 | 0 | 0 | 0 | 0 | 0 | 1 | 1.383014 |
|  | Dph4 | 0 | 0 | 0 | 0 | 0 | 0 | 0 | 0 | 0 | 0 | 1 | 0.309397 |
|  | Elp3 | 1 | 0.733111 | 0 | 0 | 0 | 0 | 0 | 0 | 0 | 0 | 0 | 0 |
|  | Lto1 | 0 | 0 | 0 | 0 | 0 | 0 | 0 | 0 | 0 | 0 | 0 | 0 |
|  | Rli1 | 2 | 5.066674 | 0 | 0 | 0 | 0 | 0 | 0 | 0 | 0 | 0 | 0 |
|  | Yae1 | 0 | 0 | 0 | 0 | 0 | 0 | 0 | 0 | 0 | 0 | 0 | 0 |
|  | Lia1 | 0 | 0 | 0 | 0 | 0 | 0 | 0 | 0 | 0 | 0 | 1 | 03.31844 |
|  | Ncs6 | 0 | 0 | 1 | 1.46848 | 0 | 0 | 0 | 0 | 0 | 0 | 0 | 0 |
| Group Coefficients |  | NA | 6.302321 | NA | 1.46848 | NA | 0 | NA | 0 | NA | 0 | NA | 5.252861 |
|  |  |  |  |  |  |  |  |  |  |  |  |  |  |
| **22. TCA** | Aco1 | 1 | 4.096926 | 0 | 0 | 0 | 0 | 0 | 0 | 0 | 0 | 0 | 0 |
|  | Aco2 | 1 | 1.143937 | 0 | 0 | 0 | 0 | 0 | 0 | 0 | 0 | 0 | 0 |
|  | Bio2 | 1 | 0.299573 | 0 | 0 | 0 | 0 | 0 | 0 | 0 | 0 | 0 | 0 |
|  | Lip5 | 2 | 0.558139 | 0 | 0 | 0 | 0 | 0 | 0 | 0 | 0 | 0 | 0 |
|  | Sdh2 | 1 | 0.615539 | 1 | 0.615539 | 1 | 0.615539 | 0 | 0 | 0 | 0 | 0 | 0 |
|  | Sdh3 | 0 | 0 | 0 | 0 | 0 | 0 | 0.5 | 0.170161 | 0 | 0 | 0 | 0 |
|  | Sdh4 | 0 | 0 | 0 | 0 | 0 | 0 | 0.5 | 0.112317 | 0 | 0 | 0 | 0 |
| Group Coefficients respiring |  | NA | 6.438481 | NA | 0.73634266 | NA | 0.736343 | NA | 0.337915 | NA | 0 | NA | 0 |
|  |  |  |  |  |  |  |  |  |  |  |  |  |  |
| **23. YAP** | Yap5 | 0.5 | 0.5 | 0 | 0 | 1.5 | 1.5 | 0 | 0 | 0 | 0 | 0 | 0 |
| Group Coefficients |  | NA | 0.5 | NA | 0 | NA | 1.5 | NA | 0 | NA | 0 | NA | 0 |

**Table S6. Summary of amino acid residues and iron cofactor coefficients for protein groups and apo forms.** Coefficient Caa refers to the number of amino acids assumed to compose the protein group (holo and apo). Other coefficients indicate the assumed number of each iron group present in each form of each protein group (as obtained in Table S5). Some protein groups have three forms, including full-apo (e.g. aaCIA), partial apo- (aCIA), and holo (CIA). In these cases, some iron centers are considered to be permanent (found in partial apo) and other are considered transient (found only in holo). The concentrations of some member proteins vary depending on whether cells are grown under fermenting or respiring conditions (see Table S3, right side). In cases where there are differences, respiring conditions are indicated by (R).

| Number | Group | Caa | C_F4_ | C_F3_ | C_F2_ | C_HE_ | C_FF_ | C_FO_ |
| --- | --- | --- | --- | --- | --- | --- | --- | --- |
| 1 | AFT | 2197.8 | 0 | 0 | 0.85334 | 0 | 0 | 0 |
|  | aAFT | ″ | 0 | 0 | 0 | 0 | 0 | 0 |
|  |  |  |  |  |  |  |  |  |
| 2 | ATM | 1782.2 | 0 | 0 | 0 | 0 | 0 | 0 |
|  |  |  |  |  |  |  |  |  |
| 3 | CAT | 1669.86 | 0 | 0 | 0.234672 | 6.436402 | 0 | 0 |
|  | aCAT | ″ | 0 | 0 | 0 | 0 | 0 |  |
|  |  |  |  |  |  |  |  |  |
| 4 | CCC | 322 | 0 | 0 | 0 | 0 | 0 | 0 |
|  |  |  |  |  |  |  |  |  |
| 5 | CIA | 4667.9 | 4.707842 | 0 | 1.839388 | 0 | 0 | 0 |
|  | aaCIA | ″ | 0 | 0 | 0 | 0 | 0 | 0 |
|  | aCIA | ″ | 0 | 0 | 1.839388 | 0 | 0 | 0 |
|  |  |  |  |  |  |  |  |  |
| 6 | ETC  (R) | 4470.4  4282.1 | 0  0 | 0  0 | 1.929342  2.534638 | 12.76333  13.14938 | 0.494720  0.216644 | 0  0 |
|  | aETC | ″ | 0 | 0 | 0 | 0 | 0 | 0 |
|  |  |  |  |  |  |  |  |  |
| 7 | FT3 | 6085.8 | 0 | 0 | 0 | 2.76376 | 0 | 0 |
|  | aFT3 | ″ | 0 | 0 | 0 | 0 | 0 | 0 |
|  |  |  |  |  |  |  |  |  |
| 8 | FT5 | 2215.7 | 0 | 0 | 0 | 1.25888 | 0 | 0 |
|  | aFT5 | ″ | 0 | 0 | 0 | 0 | 0 | 0 |
|  |  |  |  |  |  |  |  |  |
| 9 | GRX | 972.8 | 0 | 0 | 2.466990 | 0 | 0 | 0 |
|  | aGRX | ″ | 0 | 0 | 0 | 0 | 0 | 0 |
|  |  |  |  |  |  |  |  |  |
| 10 | HEM (R) | 3107.4 | 0 | 0 | 0 | 7.054408 | 0 | 0 |
|  | aHEM | ″ | 0 | 0 | 0 | 0 | 0 | 0 |
|  |  |  |  |  |  |  |  |  |
| 11 | HMX | 317.0 | 0 | 0 | 0 | 1.00 | 0 | 0 |
|  | aHMX | ″ | 0 | 0 | 0 | 0 | 0 | 0 |
|  |  |  |  |  |  |  |  |  |
| 12 | ISA | 1262.7 | 1.787040 | 0 | 1.669610 | 0 | 0 | 0 |
|  | aaISA | ″ | 0 | 0 | 0 | 0 | 0 | 0 |
|  | aISA | ″ | 0 | 0 | 1.669610 | 0 | 0 | 0 |
|  |  |  |  |  |  |  |  |  |
| 13 | ISU | 2582.7 | 0.521673 | 0 | 3.795715 | 0 | 0 | 0.475566 |
|  | aaISU | ″ | 0 | 0 | 0 | 0 | 0 | 0 |
|  | aISU | ″ | 0.521673 | 0 | 0 | 0 | 0 | 0.475566 |
|  |  |  |  |  |  |  |  |  |
| 14 | LEU | 6999.1 | 6.92658 | 2.92658 | 0 | 0.103964 | 0 | 0 |
|  | aLEU | ″ | 0 | 0 | 0 | 0 | 0 |  |
|  |  |  |  |  |  |  |  |  |
| 15 | LYS | 2348.4 | 0.665707 | 0 | 3.08492 | 0 | 0 | 0.249366 |
|  | aLYS | ″ | 0 | 0 | 0 | 0 | 0 | 0 |
|  |  |  |  |  |  |  |  |  |
| 16 | MEM | 5222.6 | 0 | 0 | 0 | 8.222864 | 7.612104 | 0.250861 |
|  | aMEM | ″ | 0 | 0 | 0 | 0 | 0 | 0 |
|  |  |  |  |  |  |  |  |  |
| 17 | MRS | 1004.6 | 0 | 0 | 0 | 0 | 0 | 0 |
|  |  |  |  |  |  |  |  |  |
| 18 | NUC | 1892.8 | 0.756236 | 0 | 0 | 0 | 1.121348 | 0.269876 |
|  | aNUC | ″ | 0 | 0 | 0 | 0 | 0 | 0 |
|  |  |  |  |  |  |  |  |  |
| 19 | POL | 11654.7 | 7.743291 | 0 | 0 | 0 | 0 | 3.256706 |
|  | aPOL | ″ | 0 | 0 | 0 | 0 | 0 | 0 |
|  |  |  |  |  |  |  |  |  |
| 20 | PRO | 3029480.0 | 0 | 0 | 0 | 0 | 0 | 0 |
|  |  |  |  |  |  |  |  |  |
| 21 | RIB | 4815.7 | 6.302321 | 1.46848 | 0 | 0 | 0 | 5.252861 |
|  | aRIB | ″ | 0 | 0 | 0 | 0 | 0 | 0 |
|  |  |  |  |  |  |  |  |  |
| 22 | TCA  (R) | 4589.6  4588.8 | 6.714114  6.438481 | 0.615539  0.736343 | 0.615539  0.736343 | 0.282478  0.337915 | 0  0 | 0  0 |
|  | aTCA | ″ | 0 | 0 | 0 | 0 | 0 |  |
|  |  |  |  |  |  |  |  |  |
| 23 | YAP | 245.0 | 0.5 | 0 | 1.5 | 0 | 0 | 0 |
|  | aYAP | ″ | 0 | 0 | 0 | 0 | 0 | 0 |

**Table S7: Iron and carbon molar equivalents for model components.** This table defines the numbers of iron and carbon atoms in each component molecule. For example, component AA molecules contains 5 carbon atoms and 0 iron atoms. Some entries include the equation used in calculation. For example, component aCIA contains 3.678776 iron atoms per molecule, obtained from the number of [Fe_2_S_2_] clusters per molecule (1.839388 as given in Table S6) multiplied by 2 because there are two irons per cluster. Each aCIA protein also contains 23339.5 carbon atoms, obtained from the number of amino acids per protein (4667.9 as given in Table S6) multiplied by 5 carbons per amino acid.

| Number | Component | Carbon equivalents | Iron equivalents |
| --- | --- | --- | --- |
| 01 | AA | 5.0 | 0 |
| 02 | aaCIA | 23339.5 | 0 |
| 03 | aAFT | 10989.0 | 0 |
| 04 | aaISA | 6313.5 | 0 |
| 05 | aaISU | 12913.5 | 0 |
| 06 | AAM | 5.0 | 0 |
| 07 | ACA | 5.0 | 0 |
| 08 | ACAC | 5.0 | 0 |
| 09 | aCAT | 8349.3 | 0 |
| 10 | aCIA | 23339.5 | 1.839388(2) = 3.678776 |
| 11 | ADP | 10.0 | 0 |
| 12 | ADPM | 10.0 | 0 |
| 13 | aETC | 21410.5 | 0 |
| 14 | AFT | 10989.0 | 1.70668 |
| 15 | aFT3 | 30429 | 0 |
| 16 | aFT5 | 11078.5 | 0 |
| 17 | aGRX | 4864 | 0 |
| 18 | aHEM | 15537 | 0 |
| 19 | aHMX | 1585 | 0 |
| 20 | aISA | 6313.5 | 3.33922 |
| 21 | aISU | 12913.5 | 2.562258 |
| 22 | aLEU | 34995.5 | 0 |
| 23 | aLYS | 11742 | 0 |
| 24 | aMEM | 26113 | 0 |
| 25 | aNUC | 9464 | 0 |
| 26 | aPOL | 58273.5 | 0 |
| 27 | aRIB | 24078.5 | 0 |
| 28 | aTCA | 22944.0 | 0 |
| 29 | ATM | 8911.0 | 0 |
| 30 | ATP | 10.0 | 0 |
| 31 | ATPM | 10.0 | 0 |
| 32 | aYAP | 1225 | 0 |
| Nutrient | CARBON | 1.0 | 0 |
| 33 | CAT | 8349.3 + 6.436402(35) = 8574.57 | 0.234672(2)+6.436402 = 6.905746 |
| 34 | CCC | 1610.0 | 0 |
| 35 | CIA | 23339.5 | 4.707842(4)+ 1.839388(2) = 22.510144 |
| 36 | CO2 | 1.0 | 0 |
| 37 | CP | 0 | 1 |
| 38 | DNA | 77500.0 | 0 |
| 39 | ETC | 21410.5+13.14938(35) = 21870.7 | 2.534638(2)+13.14938+0.216644(2)=18.652 |
| 40 | F2 | 0 | 1 |
| 41 | F3 | 0 | 1 |
| 42 | FC | 0 | 1 |
| 43 | FT3 | 30429 +2.76376(35) = 32025.7 | 2.76376 |
| 44 | FT5 | 11078.5 + 44.1 = 11122.6 | 1.25888 |
| 45 | FM | 0 | 1 |
| 46 | GRX | 4864 | 4.93398 |
| 47 | HEM | 15537+7.054408(35)=15783.9 | 7.054408 |
| 48 | HEME | 35 | 1 |
| 49 | HMX | 1585+35=1620 | 1.00 |
| Nutrient | IRON | 0 | 1 |
| 50 | ISA | 6313.5 | 1.787040(4)+1.66961(2)= 10.487 |
| 51 | ISU | 12913.5 | 0.521673(4)+3.795715(2)+0.475566 = 10.153688 |
| 52 | LEU | 34995.5+0.103964(35)=34999.1387 | 6.92658(4)+2.92658(3)+0.103964 = 36.58996 |
| 53 | LYS | 11742 | 0.665707(4)+3.08492(2)+0.249366 = 9.082034 |
| 54 | MEM | 26113+8.222864(35)= 26400.80 | 8.222864+7.612104(2)+0.250861= 23.69787 |
| 55 | MP | 0 | 1 |
| 56 | MRS | 5023.0 | 0 |
| 57 | NAD | 10.0 | 0 |
| 58 | NADM | 10.0 | 0 |
| 59 | NADV | 10.0 | 0 |
| 60 | NAH | 10.0 | 0 |
| 61 | NAHM | 10.0 | 0 |
| 62 | NAHV | 10.0 | 0 |
| 63 | NUC | 9464 | 0.756236(4)+1.121348(2)+0.269876 = 5.537516 |
| 64 | NUCM | 10.0 | 0 |
| 65 | NUCMN | 10.0 | 0 |
| 66 | O2 | 0 | 0 |
| 67 | O2M | 0 | 0 |
| 68 | O2V | 0 | 0 |
| Nutrient | OXYGEN | 0 | 0 |
| 69 | PL | 50.0 | 0 |
| 70 | POL | 58273.5 | 7.743291(4)+3.256706= 34.22987 |
| 71 | PRO | 15147400 | 0 |
| 72 | RIB | 24078.5 | 6.302321(4)+1.46848(3)+5.252861= 34.8667 |
| 73 | ROS | 0 | 0 |
| 74 | ROSE | 0 | 0 |
| 75 | ROSM | 0 | 0 |
| 76 | ROSN | 0 | 0 |
| 77 | TCA | 22944+0.337915(35)= 22955.827 | 6.438481(4)+0.736343(3)+0.736343(2)+0.337915= 29.773 |
| 78 | TCAM | 10.0 | 0 |
| 79 | TCAMC | 10.0 | 0 |
| 80 | YAP | 1225 | 0.5(4)+1.5(2) = 5.00 |

**Table S8 Derivation of amino acid content of protein groups:** The stoichiometric coefficients for the 3 groups that are most involved in respiration (ETC, HEM and TCA) change when respiration is assumed, as indicated.

| Group | Members | C_aa,i_ | [P]i/[P]g ferm | C_aa,g_ (fer) | [P]i/[P]g res | C_aa,g_ (res) |
| --- | --- | --- | --- | --- | --- | --- |
|  |  |  |  |  |  |  |
| **1. AFT** | Aft1 | 690 | 1.54866 | 1223.35 |  |  |
|  | Aft2 | 416 | 0.15802 | 75.25 |  |  |
|  | Cth1 | 325 | 1.04301 | 388.05 |  |  |
|  | Cth2 | 285 | 1.25046 | 407.95 |  |  |
| Group coefficient |  |  |  | 2197.75 |  |  |
|  |  |  |  |  |  |  |
| **2. ATM** | Atm1 | 690 | 1.4782 | 1020.0 |  |  |
|  | Mmt1 | 510 | 0.99176 | 505.8 |  |  |
|  | Mmt2 | 484 | 0.52985 | 256.4 |  |  |
| Group coefficient |  |  |  | 1782.2 |  |  |
|  |  |  |  |  |  |  |
| **3. CAT** | Aim32 | 311 | 0.110888 | 34.48 |  |  |
|  | Ccp1 | 361 | 0.794864 | 286.9 |  |  |
|  | Cta1 | 515 | 0.11694 | 60.2 |  |  |
|  | Ctt1 | 562 | 0.544604 | 306.0 |  |  |
|  | Grx6 | 231 | 0.247567 | 57.18 |  |  |
|  | Yhb1 | 399 | 4.18513 | 1669.86 |  |  |
|  |  |  |  |  |  |  |
| Group coefficient |  |  |  | 2414.77 |  |  |
|  |  |  |  |  |  |  |
| **4. CCC** | Ccc1 | 322 | 1.00 | 322 |  |  |
| Group coefficient |  |  |  | 322 |  |  |
|  |  |  |  |  |  |  |
| **5. CIA** | Cfd1 | 293 | 0.384872 | 112.7 |  |  |
|  | Cia1 | 330 | 0.73839 | 243.6 |  |  |
|  | Cia2 | 231 | 0.77058 | 178.0 |  |  |
|  | Met18 | 1032 | 0.821815 | 848.1 |  |  |
|  | Dre2 | 348 | 1.226259 | 426.7 |  |  |
|  | Mms19  Discovered to be the same as Met18 post-analysis | 1032 | 0.821982 | 848.3 |  |  |
|  | Nar1 | 491 | 0.360456 | 176.9 |  |  |
|  | Nbp35 | 349 | 2.12091 | 740.2 |  |  |
|  | Tah18 | 623 | 1.754735 | 1093.2 |  |  |
| Group coefficient |  |  |  | 4667.9 |  |  |
|  |  |  |  |  |  |  |
| **6. ETC** | Cob1 | 385 | 1.77286 | 682.5 | 2.329058 | 896.7 |
|  | Coq6 | 479 | 0.882123 | 422.5 | 0.3862885 | 185.0 |
|  | Coq7 | 233 | 0.494720 | 115.3 | 0.2166435 | 50.5 |
|  | Cox1 | 534 | 0.445360 | 237.8 | 0.585087 | 312.4 |
|  | Cox10 | 462 | 0.1372332 | 63.4 | 0.060096 | 27.8 |
|  | Cox15 | 486 | 1.0978874 | 533.6 | 0.480777 | 233.6 |
|  | Cyb2 | 591 | 1.3028076 | 769.9 | 1.711539 | 1011.5 |
|  | Cyc1 | 109 | 1.724797 | 188.0 | 2.265988 | 246.9 |
|  | Cyc2 | 366 | 0.246613 | 90.3 | 0.1079945 | 39.5 |
|  | Cyc7 | 113 | 0.596808 | 67.4 | 0.261347 | 29.5 |
|  | Cyt1 | 309 | 1.168000 | 360.9 | 1.534434 | 474.1 |
|  | Mss51 | 436 | 1.201469 | 523.8 | 0.526136 | 229.4 |
|  | Rip1 | 215 | 1.9293419 | 414.8 | 2.534638 | 544.9 |
| Group coefficient |  |  |  | 4470.4 |  | 4282.1 |
|  |  |  |  |  |  |  |
| **7. FT3** | Fet3 | 636 | 3.44994 | 2194.2 |  |  |
|  | Fet4 | 552 | 0.345979 | 191.0 |  |  |
|  | Fre1 | 686 | 0.091762 | 62.9 |  |  |
|  | Fre2 | 711 | 0.546672 | 388.7 |  |  |
|  | Fre3 | 711 | 0.546672 | 388.7 |  |  |
|  | Fre4 | 719 | 0.488441 | 351.2 |  |  |
|  | Fre5 | 694 | 0.946951 | 657.2 |  |  |
|  | Fre7 | 620 | 0.051500 | 31.9 |  |  |
|  | Ftr1 | 686 | 2.576933 | 1767.8 |  |  |
|  | Smf1 | 404 | 0.129283 | 52.2 |  |  |
| Group coefficient |  |  |  | 6085.8 |  |  |
|  |  |  |  |  |  |  |
| **8. FT5** | Fet5 | 622 | 1.2311 | 765.7 |  |  |
|  | Fre6 | 712 | 0.62944 | 448.2 |  |  |
|  | Fth1 | 465 | 1.26879 | 589.9 |  |  |
|  | Smf3 | 473 | 0.87071 | 411.8 |  |  |
| Group coefficient |  |  |  | 2215.7 |  |  |
|  |  |  |  |  |  |  |
| **9. GRX** | Bol2 | 120 | 0.644423 | 77.3 |  |  |
|  | Grx3 | 250 | 1.580181 | 395.0 |  |  |
|  | Grx4 | 244 | 0.841427 | 205.3 |  |  |
|  | Apd1 | 316 | 0.933974 | 295.1 |  |  |
| Group coefficient |  |  |  | 972.8 |  |  |
|  |  |  |  |  |  |  |
| **10. HEM** | Hem15 | 393 | 2.043452 | 803.0766 | 4.057519 | 1594.605 |
|  | Hap1 | 1502 | 0.331318 | 497.6396 | 0.21929 | 329.3736 |
|  | Hap2 | 265 | 0.388763 | 103.0222 | 0.257312 | 68.18768 |
|  | Hap3 | 144 | 0.400335 | 57.64824 | 0.264971 | 38.15582 |
|  | Hap4 | 554 | 0.185272 | 102.6407 | 0.122627 | 67.93536 |
|  | Hap5 | 242 | 0.336777 | 81.50003 | 0.222904 | 53.94277 |
|  | Rox1 | 368 | 0.302773 | 111.4205 | 0.200397 | 73.7461 |
|  | Tdh3 | 332 | 4.011299 | 1331.751 | 2.654972 | 881.4507 |
| Group coefficient |  |  |  | 3088.699 |  | 3107.4 |
|  |  |  |  |  |  |  |
| **11. HMX** | Hmx1 | 317 | 1.00 | 317 |  |  |
| Group coefficient |  |  |  | 317 |  |  |
|  |  |  |  |  |  |  |
| **12. ISA** | Bol1 | 110 | 1.28043 | 140.8 |  |  |
|  | Bol3 | 118 | 0.800688 | 94.5 |  |  |
|  | Iba57 | 497 | 0.344862 | 171.4 |  |  |
|  | Isa1 | 250 | 0.466131 | 116.5 |  |  |
|  | Isa2 | 185 | 0.791972 | 146.5 |  |  |
|  | Nfu1 | 256 | 2.315978 | 592.9 |  |  |
| Group coefficient |  |  |  | 1262.7 |  |  |
|  |  |  |  |  |  |  |
| **13. ISU** | Acp1 | 125 | 3.08857 | 386.1 |  |  |
|  | Arh1 | 493 | 0.314353 | 155.0 |  |  |
|  | Grx5 | 150 | 1.0403346 | 156.1 |  |  |
|  | Isd11 | 94 | 1.0265038 | 96.5 |  |  |
|  | Isu1 | 165 | 1.0883209 | 179.6 |  |  |
|  | Isu2 | 156 | 0.6383319 | 99.6 |  |  |
|  | Jac1 | 184 | 0.3851734 | 70.9 |  |  |
|  | Mge1 | 228 | 1.9189090 | 437.5 |  |  |
|  | Nfs1 | 497 | 1.0265038 | 510.2 |  |  |
|  | Ssq1 | 657 | 0.4888763 | 321.2 |  |  |
|  | Yah1 | 172 | 0.5085601 | 87.5 |  |  |
|  | Yfh1 | 174 | 0.4755658 | 82.7 |  |  |
| Group coefficient |  |  |  | 2582.7 |  |  |
|  |  |  |  |  |  |  |
| **14. LEU** | Glt1 | 2145 | 2.92658 | 6277.5 |  |  |
|  | Leu1 | 779 | 0.606071 | 472.1 |  |  |
|  | Met5 | 1442 | 0.103964 | 149.9 |  |  |
|  | Met8 | 274 | 0.363387 | 99.6 |  |  |
| Group coefficient |  |  |  | 6999.1 |  |  |
|  |  |  |  |  |  |  |
| **15. LYS** | Ilv3 | 585 | 3.08492 | 1804.7 |  |  |
|  | Lys4 | 693 | 0.665707 | 461.3 |  |  |
|  | Thi4 | 326 | 0.171167 | 55.8 |  |  |
|  | Thi5 | 340 | 0.078199 | 26.6 |  |  |
| Group coefficient |  |  |  | 2348.4 |  |  |
|  |  |  |  |  |  |  |
| **16. MEM** | Cyb5 | 120 | 0.738726 | 88.6 |  |  |
|  | Cyp51 | 530 | 2.086183 | 1105.7 |  |  |
|  | Dap1 | 152 | 0.592088 | 90.0 |  |  |
|  | Erg3 | 365 | 0.948603 | 346.2 |  |  |
|  | Erg5 | 538 | 1.085614 | 584.1 |  |  |
|  | Erg25 | 309 | 3.004924 | 928.5 |  |  |
|  | Fre8 | 770 | 0.095778 | 73.7 |  |  |
|  | Mpo1 | 174 | 0.095778 | 16.7 |  |  |
|  | Ncp1 | 691 | 0.250861 | 173.3 |  |  |
|  | Ole1 | 510 | 1.617924 | 825.1 |  |  |
|  | Scs7 | 384 | 1.192161 | 457.8 |  |  |
|  | Sfh5 | 294 | 0.518596 | 152.5 |  |  |
|  | Sur2 | 349 | 0.848492 | 296.1 |  |  |
|  | Yno1 | 570 | 0.147897 | 84.3 |  |  |
| Group coefficient |  |  |  | 5222.6 |  |  |
|  |  |  |  |  |  |  |
| **17. MRS** | Mrs3 | 314 | 1.001879 | 314.6 |  |  |
|  | Mrs4 | 304 | 0.8665976 | 263.4 |  |  |
|  | Rim2 | 377 | 1.131524 | 426.6 |  |  |
| Group coefficient |  |  |  | 1004.6 |  |  |
|  |  |  |  |  |  |  |
| **18. NUC** | Bna1 | 177 | 0.2698758 | 47.8 |  |  |
| (removed Ade4) | Rnr2 | 399 | 1.1213483 | 447.4 |  |  |
|  | Rnr4 | 345 | 3.0262073 | 1044.0 |  |  |
|  | Tyw1 | 810 | 0.1529716 | 123.9 |  |  |
| Group coefficient used |  |  |  | 1892.8 |  |  |
|  |  |  |  |  |  |  |
| **19. POL** | Chl1 | 861 | 0.014336 | 12.3 |  |  |
|  | Dna2 | 1522 | 0.733248 | 1116.0 |  |  |
|  | Ntg2 | 380 | 0.035862 | 13.6 |  |  |
|  | Pol1 | 1468 | 1.018744 | 1495.5 |  |  |
|  | Pol2 | 2222 | 1.275885 | 2835.0 |  |  |
|  | Pol3 | 1097 | 1.277963 | 1401.9 |  |  |
|  | Pri2 | 528 | 0.963634 | 508.8 |  |  |
|  | Rad3 | 778 | 1.155082 | 898.7 |  |  |
|  | Rev3 | 1504 | 0.580456 | 873.0 |  |  |
|  | Tpa1 | 644 | 3.256706 | 2097.3 |  |  |
|  | Exo5 | 585 | 0.688081 | 402.5 |  |  |
| Group coefficient |  |  |  | 11654.7 |  |  |
|  |  |  |  |  |  |  |
| **20. PRO** | P_1_ | 530 | 1.00 | 530 |  |  |
|  | P_2_ etc | 530 | 1.00 | 530 |  |  |
|  | P_5716_ | 530 | 1.00 | 530 etc |  |  |
| Group coefficient |  |  |  | 3029480 |  |  |
|  |  |  |  |  |  |  |
| **21. RIB** | Cbr1 | 284 | 1.468477 | 417.0 |  |  |
|  | Dbr1 | 405 | 0.24201 | 98.0 |  |  |
|  | Dph1 | 425 | 0.45393 | 192.9 |  |  |
|  | Dph2 | 534 | 0.551141 | 294.3 |  |  |
|  | Dph3 | 82 | 1.383014 | 113.4 |  |  |
|  | Dph4 | 172 | 0.309397 | 53.2 |  |  |
|  | Elp3 | 557 | 0.733111 | 408.3 |  |  |
|  | Lto1 | 198 | 0.156923 | 31.1 |  |  |
|  | Rli1 | 608 | 2.533337 | 1540.3 |  |  |
|  | Yae1 | 141 | 0.435618 | 61.4 |  |  |
|  | Lia1 | 325 | 3.318437 | 1078.5 |  |  |
|  | Ncs6 | 359 | 1.468477 | 527.2 |  |  |
| Group coefficient |  |  |  | 4815.7 |  |  |
|  |  |  |  |  |  |  |
| **22. TCA** | Aco1 | 778 | 4.0969256 | 3187.4 | 4.900968 | 3813.0 |
|  | Aco2 | 789 | 1.1439367 | 902.6 | 0.4561467 | 359.9 |
|  | Bio2 | 375 | 0.2995734 | 112.3 | 0.11943358 | 44.8 |
|  | Lip5 | 414 | 0.27906964 | 115.5 | 0.11127949 | 46.1 |
|  | Sdh2 | 266 | 0.61553986 | 163.7 | 0.73634266 | 195.9 |
|  | Sdh3 | 198 | 0.34032145 | 67.4 | 0.40711124 | 80.6 |
|  | Sdh4 | 181 | 0.22463321 | 40.7 | 0.26871859 | 48.6 |
| Group coefficient |  |  |  | 4589.6 |  | 4588.8 |
|  |  |  |  |  |  |  |
| **23. YAP** | Yap5 | 245 | 1.00 | 245 |  |  |
| Group coefficient |  |  |  | 245 |  |  |

**Table S9: Model Reactions.** The reaction network included the 169 reactions listed below. Calculations explaining stoichiometric coefficients are included in non-obvious cases. These values were then included in the ***S_cell_*** matrix. Both carbon and iron are conserved in each reaction, assuming the contents indicated in Tables S6 and S7; no other elements are conserved.

| Number | Name and Description | Reaction and Stoichiometry Calculation | Catalyst(s) | Compartment(s) | S Matrix Entries |
| --- | --- | --- | --- | --- | --- |
| 001 | AATM  Transfers [Fe_2_S_2_] clusters from ISU to aGRX upon exiting from mitochondria via ATM, in an ATP-dependent process. | ISU + 1.538601⋅aGRX + 1.538601⋅ATPM→ aISU + 1.538601⋅GRX + 1.538601⋅ADPM    Carbon:  12913.5 + 1.538601⋅(4864) + 1.538601(10) → 12913.5 + 1.538601⋅(4864) + 1.538601(10)  Iron:  10.153688 + 1.538601⋅(0) + 1.538601⋅(0) → 2.562258 + 1.538601⋅4.93398 + 1.538601⋅(0) | ATM, PL | M:  ISU  ATPM  aISU  ADPM  C:  aGRX  GRX | ISU = -1⋅6.43 = -6.43  aGRX = -1.538601  ATPM = -1.538601⋅6.43 = -9.89320443  aISU = +1⋅6.43 = +6.43  GRX = +1.538601  ADPM =1.538601⋅6.43 = +9.89320443 |
| 002 | ACAT  Degradation of reactive oxygen species in the cytosol. | ROS →  Carbon: 0 → 0  Iron: 0 → 0 | CAT | C:  ROS | ROS = -1 |
| 003 | ACCC  Import of cytosolic Fe^II^ into vacuoles, forming Fe^II^ in the organelle. | FC → F2  Carbon: 0 → 0  Iron: 1 → 1 | CCC, PL | V:  F2  C:  FC | FC = -1  F2 = +1⋅7.84146 = +7.84146 |
| 004 | ACETC  Activity of the electron transport chain in mitochondria, generating ATP and producing ROS as a side-product | 2⋅NAHM + O2M + 4⋅ADPM → 2⋅NADM + 4⋅ATPM + 0.05⋅ROSM  Carbon: 2(10) + 0 + 4(10) → 2(10)+4(10)+ 0.05(0)  Iron: 0 → 0 | ETC, PL | M:  NAHM  O2M  ADPM  NADM  ATPM  ROSM | NAHM = -2⋅ 6.43 = -12.86  O2M = -1⋅6.43 = -6.43  ADPM = -4⋅6.43 = -25.72  NADM = +2⋅6.43 = +12.86  ATPM = 4⋅6.43 = +25.72  ROSM = 0.05⋅6.43 = 0.3215 |
| 005 | ACFT3  Import of nutrient iron into the labile Fe^II^ pool in the cytosol. | IRON → FC  Carbon: 0 → 0  Iron: 1 → 1 | FT3, PL | Env:  IRON  C:  FC | FC = 1 |
| 006 | ACFT5  Export of Fe^II^ from vacuoles to cytosol, replenishing the labile Fe^II^ pool. | F2 → FC  Carbon: 0 → 0  Iron: 1 → 1 | FT5, PL | V:  F2  C:  FC | F2 = -1⋅ 7.84146 = -7.84146  FC = +1 |
| 007 | ACLEU  Synthesis of the amino acid AA in the cytosol. | TCAMC + ATP + NAH → 2AA + ADP + NAD  Carbon: 10 + 10 + 10 → 2(5)+10+10  Iron: 0 → 0 | LEU | C:  TCAMC  ATP  NAH  AA  ADP  NAD | TCAMC = -1  ATP = -1  NAH = -1  AA = 2  ADP = 1  NAD = 1 |
| 008 | ACLYS  Synthesis of the amino acid AA in the mitochondria. | TCAM + ATPM + NAHM → 2AAM + ADPM + NADM  Carbon: 10 + 10 + 10 → 2(5)+10+10  Iron: 0 → 0 | LYS | M:  TCAM  ATPM  NAHM  AAM  ADPM  NADM | TCAM = -1⋅ 6.43 = -6.43  ATPM = -1⋅ 6.43 = -6.43  NAHM = -1⋅ 6.43 = -6.43  2AAM = 2⋅ 6.43 = +12.86  ADPM = 1⋅ 6.43 = +6.43  NADM = 1⋅6.43 = +6.43 |
| 009 | ACMEM  Synthesis of phophoplipid PL, the sole component of membranes. | 10ACAC + 28ATP + 36NAH + 4O2 → PL + 28ADP + 36NAD  Carbon: 10(5) + 28(10) + 36(10) +4(0) → 50 +28(10)+36(10)  Iron: 0 → 0 | MEM | E:  PL  C:  ACAC  ATP  NAH  O2  ADP  NAD | ACAC = -10  ATP = -28  NAH = -36  O2 = -4  PL =1⋅26.7916 = 26.7916  ADP = +28  NAD = +36 |
| 010 | ACMRS  Import of cytosolic Fe^II^ into mitochondria, forming the labile Fe^II^ pool in the organelle. | FC → FM  Carbon: 0 → 0  Iron: 1 → 1 | MRS, PL, ETC | M:  FM  C:  FC | FC = -1  FM = 1⋅6.43 = 6.43 |
| 011 | ACNUC  Synthesis of the sole nucleotide NUC. | 2AA + 5ATP → NUCM + 5ADP  Carbon: 2(5)+5(10) → 10 +5(10)  Iron: 0 → 0 | NUC | C:  AA  ATP  NUCM  ADP | AA = -2  ATP = -5  NUCM = 1  ADP = 5 |
| 012 | ACPOL  Synthesis of DNA from nucleotide NUC. | 7750⋅NUCMN → DNA  Carbon: 7750(10) → 77500  Iron: 0 → 0 | DNA, POL | N:  NUCMN  DNA | NUCMN = -7750⋅4.2583 = -33,001.825  DNA = +1⋅4.2583 |
| 013 | ACTCA  Activity of the TCA cycle; regenerates NAH (i.e. NADH) from NAD. | ACA + 3⋅NADM + ADPM → 3⋅NAHM + ATPM + 5⋅CO2  Carbon: 5 +3(10) +10 → 3(10)+10+5(1)  Iron: 0 → 0 | TCA, TCAM | M:  ACA  NADM  ADPM  NAHM  ATPM  C:  CO2 | ACA = -1⋅6.43 = -6.43  NADM = -3⋅6.43 = -19.29  ADPM = -1⋅6.43 = -6.43  NAHM = 3⋅6.43 = 19.29  ATPM = 1⋅6.43 = 6.43  CO2 = 5⋅1 = 5 |
| 014 | AF2  Oxidation of vacuolar Fe^II^ to vacuolar Fe^III^ using O2 in the vacuole. | 4F2 + O2V → 4F3  Carbon: 4(0)+ 0 → 4(0)  Iron: 4(1)+0 → 4(1) | None | V:  F2  O2V  F3 | F2 = -4⋅7.84146 = -31.36584  O2V = -1⋅7.84146 = -7.84146  F3 = 4⋅7.84146 = +31.36584 |
| 015 | AF3  Reduction of vacuolar Fe^III^ to Fe^II^ using NADH in the vacuole. | 2F3 + NAHV → 2F2 + NADV  Carbon: 2(0)+10 → 2(0)+10  Iron: 2(1)+0 → 2(1)+0 | None | V:  F3  NAHV  F2  NADV | F3 = -2⋅7.84146 = -15.68292  NAHV = -1⋅7.84146 = -7.84146  F2 = 2⋅7.84146 = 15.68292  NADV = 1⋅7.84146 = 7.84146 |
| 016 | AFC  Oxidation of the cytosolic labile Fe^II^ pool to cytosolic Fe^III^ nanoparticles and ROS (the occurrence of this reaction is not established) | 3FC + O2 → 3CP + ROS  Carbon: 2(0)+10 → 2(0)+10  Iron: 2(1)+0 → 2(1)+0 | None | C:  FC  O2  CP  ROS | FC = -3  O2 = -1  CP = 3  ROS = 1 |
| 017 | AFM  Oxidation of the mitochondrial Fe^II^ pool forming Fe^III^ nanoparticles and ROS in the organelle. | 3FM + O2M → 3MP + ROSM  Carbon: 3(0)+0 → 3(0)+0  Iron: 3(1)+0 → 3(1)+0 | None | M:  FM  O2M  MP  ROSM | FM = -3⋅6.43 = -19.29  O2M = -1⋅6.43 = -6.43  MP = 3⋅6.43= +19.29  ROSM = +6.43 |
| 018 | AROSE  Degradation of phospholipid (membranes) by ROS. | PL + ROSE → 10ACAC  Carbon: 50+0 → 10(5)  Iron: 0+0 → 0 | None | E:  PL  ROSE  C:  ACAC | PL = -1⋅26.7916 = -26.7916  ROSE = -1⋅26.7916 = -26.7916  ACAC = 10 |
| 019 | AROSN  Degradation of DNA by ROS in the nucleus. | DNA + ROSN → 15500AA  Carbon: 77500+0 → 15500(5)  Iron: 0+0 → 15500(0) | none | N:  DNA  ROSN  C:  AA | DNA = -1⋅4.2583 = -4.2583  ROSN = -1⋅4.2583 = -4.2583  AA = 15500 |
| 020 | ATCAA  Synthesis of the TCA cycle metabolite TCAM. | 2ACA → TCAM  Carbon: 2(5) → 10  Iron: 2(0) → 0 | None | M:  ACA  TCAM | ACA = -2⋅6.43 = -12.86  TCAM = 1⋅6.43 = 6.43 |
| 021 | BAACIA  Biosynthesis of aaCIA. | 4667.9⋅AA + 14003.7⋅ATP → aaCIA + 14003.7⋅ADP  Carbon: 4667.9(5)+14003.7(10) → 23339.5+ 14003.7(10)  Iron: 4667.9(0) + 14003.7(0) → 0 + 14003.7(0) | DNA, RIB | C:  AA  ATP  aaCIA  ADP | AA = -4667.9  ATP = -14003.7  aaCIA = 1  ADP = 14003.7 |
| 022 | BAAFT  Biosynthesis of aAFT. | 2197.8⋅AA + 6593.4⋅ATP → aAFT+ 6593.4⋅ADP  Carbon: 2197.8(5)+6593.4(10) → 10989.0+ 6593.4(10)  Iron: 2197.8(0) + 6593.4(0) → 0 + 6593.4(0) | DNA, RIB | C:  AA  ATP  aAFT  ADP | AA = -2197.8  ATP = -6593.4  aAFT = 1  ADP = 6593.4 |
| 023 | BAAISA  Biosynthesis of aaISA. | 1262.7⋅AA + 3787.8⋅ATP→ aaISA+ 3787.8⋅ADP  Carbon: 1262.7(5)+3787.8(10) → 6313.5+ 3787.8(10)  Iron: 1262.7(0) +3787.8(0) → 0 + 3787.8(0) | DNA, RIB | C:  AA  ATP  aaISA  ADP | AA = -1262.7  ATP = -3787.8  aaISA = 1  ADP = 3787.8 |
| 024 | BAAISU  Biosynthesis of aaISU. | 2582.7⋅AA + 7748.1⋅ATP → aaISU + 7748.1⋅ADP  Carbon: 2582.7(5)+7781.1(10) → 12913.5+ 7748.1(10)  Iron: 2582.7(0) +7748.1(0) → 0 + 7748.1(0) | DNA, RIB | C:  AA  ATP  aaISU  ADP | AA = -2582.7  ATP = -7748.1  aaISU = 1  ADP = 7748.1 |
| 025 | BACAC  Import of nutrient carbon into the cytosol and conversion into acetyl-CoA. | CARBON → 0.2⋅ACAC  Carbon: 1 → 0.2(5)  Iron: 0 → 0.2(0) | None | Env:  CARBON  C:  ACAC | ACAC = 0.2 |
| 026 | BACAT  Biosynthesis of aCAT. | 1669.86⋅AA + 5009.7⋅ATP → aCAT + 5009.7⋅ADP  Carbon: 1669.86(5)+5009.7(10) → 8349.3+ 5009.7(10)  Iron: 1669.86(0) +5009.7(0) → 0 + 5009.7(0) | DNA, RIB  ROS | C:  AA  ATP  aCAT  ADP | AA = -1669.86  ATP = -5009.7  aCAT = 1  ADP = 5009.7 |
| 027 | BADP  Biosynthesis of ADP from acetyl-CoA in a process that requires ATP. | 2ACAC + ATP → 2ADP  Carbon: 2(5)+10 → 2(10)  Iron: 2(0) +0 → 2(0) | None | C:  ACAC  ATP  ADP | ACAC = -2  ATP = -1  ADP = 2 |
| 028 | BAETC  Biosynthesis of aETC. | 4282.1⋅AA + 128463⋅ATP → aETC + 128463⋅ADP  Carbon: 4282.1(5)+128463(10) → 21410.5+ 128463(10)  Iron: 4282.1(0) +128463(0) → 0 + 128463(0) | DNA, RIB, HEM | C:  AA  ATP  aETC  ADP | AA = -4282.1  ATP = -128463  aETC = 1  ADP = 128463 |
| 029 | BAFT3  Biosynthesis of aFT3. | 6085.8⋅AA + 18257.4⋅ATP → aFT3 + 18257.4⋅ADP  Carbon: 6085.8(5)+18257.4(10) → 30429 +18257.4(10)  Iron: 6085.8(0) +18257.4(10) → 0 + 18257.4(0) | DNA, RIB, PL, aAFT | C:  AA  ATP  aFT3  ADP | AA = -6085.8  ATP = -18257.4  aFT3 = 1  ADP = 18257.4 |
| 030 | BAFT5  Biosynthesis of aFT5. | 2215.7⋅AA + 6647.1⋅ATP → aFT5 + 6647.1⋅ADP  Carbon: 2215.7(5)+6647.1(10) → 11078.5 +6647.1(10)  Iron: 2215.7(0) +6647.1(0) → 0 + 6647.1(0) | DNA, RIB, PL, aAFT | C:  AA  ATP  aFT5  ADP | AA = -2215.7  ATP = -6647.1  aFT5 = 1  ADP = 6647.1 |
| 031 | BAGRX  Biosynthesis of aGRX. | 972.8⋅AA + 2918.4⋅ATP → aGRX + 2918.4⋅ADP  Carbon: 972.8(5)+2918.4(10) → 4864 +2918.4(10)  Iron: 972.8(0) +2918.4(0) → 0 +2918.4(0) | DNA, RIB, YAP | C:  AA  ATP  aGRX  ADP | AA = -972.8  ATP = -2918.4  aGRX = 1  ADP = 2918.4 |
| 032 | BAHEM  Biosynthesis of aHEM. | 3107.4⋅AA + 9322.2⋅ATP → aHEM + 9322.2⋅ADP  Carbon: 3107.4(5)+9322.2(10) → 15537 +9322.2(10)  Iron: 3107.4(0) +9322.2(0) → 0 +9322.2(0) | DNA, RIB | C:  AA  ATP  aHEM  ADP | AA = -3107.4  ATP = -9322.2  aHEM = 1  ADP = 9322.2 |
| 033 | BAHMX  Biosynthesis of aHMX, which is located in the ER rather than in the cytosol. | 317⋅AA + 951⋅ATP → aHMX + 951⋅ADP  Carbon: 317(5)+951(10) → 1585 +951(10)  Iron: 317(0) +951(0) → 0 +951(0) | DNA, RIB, aAFT | E:  aHMX  C:  AA  ATP  ADP | AA = -317  ATP = -951  aHMX = 1⋅26.7916 = 26.7916  ADP = 951 |
| 034 | BALEU  Biosynthesis of aLEU | 6999.1⋅AA + 20997.3⋅ATP → aLEU + 20997.3⋅ADP  Carbon: 6999.1(5)+20997.3(10) → 34995.5 +20997.3(10)  Iron: 6999.1(0) +20997.3(0) → 0 +20997.3(0) | DNA, RIB | C:  AA  ATP  aLEU  ATP | AA = -6999.1  ATP = -20997.3  aLEU = 1  ADP = 20997.3 |
| 035 | BALYS  Biosynthesis of aLYS. | 2348.4⋅AA +7045.2⋅ATP → aLYS + 7045.2⋅ADP  Carbon: 2348.4(5)+7045.2(10) → 11742 +7045.2(10)  Iron: 2348.4(0) +7045.2(0) → 0 +7045.2(0) | DNA, RIB | C:  AA  ATP  aLYS  ADP | AA = -2348.4  ATP = -7045.2  aLYS = 1  ADP = 7045.2 |
| 036 | BAMEM  Biosynthesis of aMEM. | 5222.6⋅AA + 15667.8⋅ATP → aMEM + 15667.8⋅ADP  Carbon: 5222.6(5)+15667.8(10) → 26113 +15667.8(10)  Iron: 5222.6(0) +15667.8(0) → 0 +15667.8(0) | DNA, RIB, PL | C:  AA  ATP  aMEM  ADP | AA = -5222.6  ATP = -15667.8  aMEM = 1  ADP = 15667.8 |
| 037 | BANUC  Biosynthesis of aNUC. | 1892.8⋅AA + 5678.4⋅ATP → aNUC + 5678.4⋅ADP  Carbon: 1892.8(5)+5678.4(10) → 26113 +5678.4(10)  Iron: 1892.8(0) +5678.4(0) → 0 +5678.4(0) | DNA, RIB | C:  AA  ATP  aNUC  ADP | AA = -1892.8  ATP = -5678.4  aNUC = 1  ADP = 5678.4 |
| 038 | BAPOL  Biosynthesis of aPOL. | 11654.7⋅AA + 34964.1⋅ATP → aPOL + 34964.1⋅ADP  Carbon: 11654.7(5)+34964.1(10) → 58273.5 +34964.1(10)  Iron: 11654.1(0) +34964.1(0) → 0 +34964.1(0) | DNA, RIB | C:  AA  ATP  aPOL  ADP | AA = -11654.7  ATP = -34964.1  aPOL = 1  ADP = 34964.1 |
| 039 | BARIB  Biosynthesis of aRIB. | 4815.7⋅AA + 14447.1⋅ATP → aRIB + 14447.1⋅ADP  Carbon: 4815.7(5)+14447.1(10) → 24078.5+14447.1(10)  Iron: 4815.7(0) +14447.1(0) → 0 +14447.1(0) | DNA, RIB | C:  AA  ATP  aRIB  ADP | AA = -4815.7  ATP = -14447.1  aRIB = 1  ADP = 14447.1 |
| 040 | BATCA  Biosynthesis of aTCA. | 4588.8⋅AA + 13766.4⋅ATP → aTCA + 13766.4⋅ADP  Carbon: 4588.8(5)+13766.4(10) → 22944.0 +13766.4(10)  Iron: 4588.8(0) +13766.4(0) → 0 +13766.4(0) | DNA, RIB | C:  AA  ATP  aTCA  ADP | AA = -4588.8  ATP = -13766.4  aTCA = 1  ADP = 13766.4 |
| 041 | BATM  Biosynthesis of ATM and installation into mitochondria | 1782.2⋅AA + 5346.6⋅ATP →ATM + 5346.6⋅ADP  Carbon: 1782.2(5)+5346.6(10) → 8911+5346.6(10)  Iron: 1782.2(0) +5346.6(0) → 0 +5346.6(0) | DNA, RIB, PL | M:  ATM  C:  AA  ATP  ADP | AA = -1782.2  ATP = -5346.6  ATM = 1⋅6.43 = 6.43  ADP = 5346.6 |
| 042 | BAYAP  Biosynthesis of aYAP. | 245⋅AA + 735⋅ATP → aYAP + 735⋅ADP  Carbon: 245(5)+735(10) → 1225+735(10)  Iron: 245(0) +735(0) → 0 +735(0) | DNA, RIB | C:  AA  ATP  aYAP  ADP | AA = -245  ATP = -735  aYAP = 1  ADP = 735 |
| 043 | BCCC  Biosynthesis of CCC and installation into vacuoles | 322⋅AA + 966⋅ATP → CCC + 966⋅ADP  Carbon: 322(5)+966(10) → 1610+966(10)  Iron: 322(0) +966(0) → 0 +966(0) | DNA, RIB, YAP, PL | V:  CCC  C:  AA  ATP  ADP | AA = -322  ATP = -966  CCC = 1⋅7.84146 = 7.84146  ADP = 966 |
| 044 | BMRS  Biosynthesis of MRS and installation into mitochondria. | 1004.6⋅AA + 3013.8⋅ATP → MRS + 3013.8⋅ADP  Carbon: 1004.6(5)+3013.8(10) → 5023+3013.8(10)  Iron: 1004.6(0) +3013.8(0) → 0 +3013.8(0) | DNA, RIB, PL | M:  MRS  C:  AA  ATP  ADP | AA = -1004.6  ATP = -3013.8  MRS = 1⋅6.43 = 6.43  ADP = 3013.8 |
| 045 | BNAD  Biosynthesis of NAD from acetyl-CoA, in an ATP-dependent process. | 2ACAC + ATP → ADP + NAD  Carbon: 2(5)+10 → 10 + 10  Iron: 2(0) +0 → 0 +0 | None | C:  ACAC  ATP  ADP  NAD | ACAC = -2  ATP = -1  NAD= 1  ADP = 1 |
| 046 | BPRO  Biosynthesis of PRO. | 3029480⋅AA + 9088440⋅ATP → PRO + 9088440⋅ADP  Carbon: 3029480(5)+9088440(10) → 15147400 +9088440(10)  Iron: 3029480(0)+9088440(0) → 0 +9088440(0) | DNA, RIB | C:  AA  ATP  PRO  ADP | AA = -3029480  ATP = -9088440  PRO = 1  ADP = 9088440 |
| 047 | CATD  Degradation of CAT under Fe-deficient conditions, releasing iron into the cytosolic Fe^II^ pool. | CAT + 6.436402⋅O2 + 6.436402⋅NAH → 6.905746⋅FC + 6.436402⋅NAD + 1669.9⋅AA + 225.27⋅CO2 $\begin{matrix} 0.234672\times2=0.469344 \\ +6.436402=6.905746 \end{matrix}$   Carbon: 8574.57 + 6.436402⋅(0) + 6.436402⋅(10) → 6.905746⋅(0) + 6.436402⋅(10) + 1669.86⋅(5) + 225.27⋅(1)  Iron: 6.905746+6.436402⋅(0) + 6.436402⋅(0) → 6.905746⋅(1) + 6.436402⋅(0) + 1669.86⋅(0) + 225.27⋅(0) | HMX, aAFT | C:  CAT  O2  NAH  FC  NAD  AA  CO2 | CAT = -1  O2 = -6.4364  NAH = -6.4364  FC = 6.90575  NAD = 6.4364  AA = 1669.9  CO2 = 225.27 |
| 048 | ETCD  Degradation of ETC under Fe-deficient conditions, releasing iron into the cytosolic Fe^II^ pool. | ETC + 13.14938⋅O2 + 13.14938⋅NAH → 18.652⋅FC + 13.14938⋅NAD + 4282.1⋅AA + 460.23⋅CO2 $\begin{matrix} 2.534638\times2 \\ +13.14938 \\ +0.216644\times2=18.651944 \end{matrix}$   Carbon: 21870.7 + 13.14938⋅(0) + 13.14938⋅(10) → 18.652⋅(0) + 13.14938⋅(10) + 4282.1⋅(5) + 460.23⋅(1)  Iron: 18.652 + 13.14938⋅(0) + 13.14938⋅(0) → 18.652⋅(1) + 13.14938⋅(0) + 4282.1⋅(0)+ 460.23⋅(0) | HMX, aAFT | M:  ETC  C:  O2  NAH  FC  NAD  AA  CO2 | ETC = -1⋅6.43 = -6.43  O2 = -13.14938  NAH = -13.14938  FC = 18.652  NAD = 13.14938  AA = 4282.1  CO2 = 460.23 |
| 049 | MAACIA  Metallation of aaCIA by GRX to load [Fe_2_S_2_] clusters into aaCIA. | aaCIA + 0.7456001⋅GRX → aCIA + 0.7456001⋅aGRX    Carbon: 23339.5 + 0.7456001⋅(4864) → 23339.5+ 0.7456001⋅(4864)  Iron: 0 + 0.7456001⋅(4.93398) → 3.678776 + 0.7456001⋅(0) | None | C:  aaCIA  GRX  aCIA  aGRX | aaCIA = -1  GRX = -0.7456  aCIA = 1  aGRX = 0.7456 |
| 050 | MAAFT  Metallation of aAFT by GRX, to load [Fe_2_S_2_] clusters into aAFT. | aAFT + 0.345903⋅GRX → AFT + 0.345903⋅aGRX    Carbon: 10989.0 + 0.345903⋅(4864) → 10989.0 + 0.345903⋅(4864)  Iron: 0 + 0.345903⋅(4.93398) → 1.70668 + 0.345903⋅(0) | None | C:  aAFT  GRX  AFT  aGRX | aAFT = -1  GRX = -0.3459  AFT = 1  aGRX = 0.3459 |
| 051 | MAAISA  Metallation of aaISA by ISU. Installs the permanent [Fe_2_S_2_] cluster only. | aaISA + 0.439867⋅ISU → aISA + 0.439867⋅aISU    Carbon: 6313.5+ 0.439867⋅(12913.5) → 6313.5 + 0.439867⋅(12913.5)  Iron: 0 + 0.439867⋅(10.153688) → 3.33922 + 0.439867⋅(2.562258) | None | M:  ISU  aISA  aISU  C:  aaISA | aaISA = -1 ISU = 0.439867⋅6.43 = -2.82834  aISA =1⋅ 6.43 = 6.43  aISU = 0.439867⋅6.43 = 2.828345 |
| 052 | MAAISU  Metallation of aaISU by ISA and FM. This installs the [Fe_4_S_4_] and mono-iron sites. | aaISU + 0.291920⋅ISA +0.475566⋅FM→ aISU + 0.291920⋅aISA    Carbon: 12913.5+ 0.291920⋅(6313.5) 0.475566(0) → 12913.5+ 0.291920⋅(6313.5)  Iron: 0 + 0.291920⋅(10.487) + 0.475566(1) →2.562258 + 0.291920⋅(3.33922) | None | M:  ISA  FM  aISU  aISA  C:  aaISU | aaISU = -1 ISA = 0.291920⋅6.43 = -1.8770456  aISA = 0.291920⋅6.43 = 1.8770456  aISU = 1⋅6.43 = 6.43 |
| 053 | MACAT  Metallation of aCAT by HEME and GRX. CAT contains heme and [Fe_2_S_2_] clusters. | aCAT + 6.436402⋅HEME + 0.0951248⋅GRX→ CAT + 0.0951248⋅aGRX    Carbon: 8349.3 + 6.436402⋅(35) + 0.0951248⋅(4864)→ 8574.57 + 0.0951248⋅(4864)  Iron: 0 + 6.436402⋅(1) + 0.0951248⋅(4.93398)→ 6.905746 + 0.0951248⋅(0) | None | C:  aCAT  HEME  GRX  CAT  aGRX | aCAT = -1  HEME = -6.436402  GRX= -0.095128  CAT = 1  aGRX = 0.095128 |
| 054 | MACIA  Metallation of aCIA by GRX to build the [Fe_4_S_4_] cluster by reductive coupling of two [Fe_2_S_2_] clusters donated by GRX. | aCIA + 3.816669⋅GRX + 4.7078421⋅NAH → CIA + 3.816669⋅aGRX + 4.7078421⋅NAD    Carbon: 23339.5 + 3.816669⋅(4864) + 6⋅(10) → 23339.5 + 3.816669⋅(4864) + 6⋅(10)  Iron: 3.678776 + 3.816669⋅(4.93398) + 6⋅(0) → 22.5101 + 3.818669⋅(0) + 6⋅(10) | None | C:  aCIA  GRX  NAH  CIA  aGRX  NAD | aCIA = -1  GRX = -3.81669  NAH = -4.7078421  CIA = 1  aGRX = 3.81667  NAD = 4.7078421 |
| 055 | MAETC  Metallation of aETC by HEM, ISU, and FM. ETC contains heme, [Fe_2_S_2_] clusters, and mono-iron centers. | aETC + 1.863995⋅HEM + 0.667763⋅ISU +0.433288⋅FM → ETC +1.863995⋅aHEM+ 0.667763⋅aISU    Carbon: 21410.5 + 1.863995⋅(15783.9) + 0.667763⋅(12913.5) +0.433288⋅(0) → 21870.7 +1.863995⋅(15537)+ 0.667763⋅(12913.5)  Iron: 0 + 1.863995⋅(7.054408) + 0.667763⋅(10.153688) +0.433288⋅(1) → 18.652 +1.863995⋅(0)+ 0.667763⋅(2.562258) | PL | M:  ETC  HEM  ISU  FM  aISU  C:  aETC  aHEM | aETC = -1  HEM = -1.863005⋅6.43 = -11.98549  ISU = -0.667763⋅6.43 = -4.293716  FM = -0.433288⋅6.43 = -2.786042  ETC = 1⋅6.43 = 6.43  aHEM = 1.863995  aISU = 0.667763⋅6.43 = 4.2937161 |
| 056 | MAFT3  Metallation of aFT3 by HEME. | aFT3 + 2.76376⋅HEME → FT3    Carbon: 30429 + 2.76376⋅(35) → 30525.7  Iron: 0 + 2.76376(1) → 2.76376 | PL | C:  aFT3  HEME  FT3 | aFT3 = -1  HEME = -2.76376  FT3 = 1 |
| 057 | MAFT5  Metallation of aFT5 by HEME. | aFT5 + 1.25888⋅HEME → FT5    Carbon: 11078.5 + 1.25888⋅(35) → 11122.6  Iron: 0 + 1.25888(1) → 1.25888 | PL | V:  FT5  C:  aFT5  HEME | aFT5 = -1  HEME = -1.25888  FT5 = 1⋅7.84146 = 7.84146 |
| 058 | MAHEM  Metallation of aHEM (and heme synthesis) using iron from the mitochondrial Fe^II^ pool. | aHEM +7.054408⋅FM +16.46029⋅TCAM+16.46029⋅AAM+7.054408⋅NAHM→HEM +7.054408⋅NADM $\begin{matrix} \frac{1 mol aHEM}{1}\left(\frac{7.054408 mol HE}{1 mol aHEM}\right)=\frac{b mol FM}{1}\left(\frac{1 mol HE}{1 mol FM}\right) \\ b=7.054408 \\ \frac{1 mol aHEM}{1}\left(\frac{7.054408 mol HE}{1 mol aHEM}\right)\left(\frac{35molC}{1molHE}\right)=\frac{c mol TCAM}{1}\left(\frac{10 mol C}{1 mol TCAM}\right)+\frac{c mol AAM}{1}\left(\frac{5 mol C}{1 mol AAM}\right) \\ c=16.46029 \end{matrix}$     Carbon: 15537 +7.054408⋅(0) +16.46029⋅(10) + 16.46029⋅(5) + 7.054408⋅(10) → 15783.9 +7.054408⋅(10)  Iron: 0 +7.054408⋅(1) + 16.46029⋅(0) + 16.46029⋅(0) + 7.054408⋅(0) →  7.054408 +7.054408⋅(0) | None | M:  FM  TCAM  AAM  NAHM  HEM  NADM  C:  aHEM | aHEM = -1  FM = -7.054408⋅6.43 = -45.35984  TCAM = -16.46029⋅6.43 = -105.8397  AAM = -16.46029⋅6.43 = -105.8387  NAHM = -7.054408⋅6.43 = -45.35984  HEM = 1⋅6.43 = 6.43  NADM = 7.054408⋅6.43 = 45.35943 |
| 059 | MAHMX  Metallation of aHMX using HEME from the cytosol. The heme originates from the cytosol, while both aHMX and HMX are located in the ER. | aHMX + HEME → HMX  Carbon: 1585 +35 → 1620  Iron: 0 +1 → 1 | None | ER:  aHMX  HMX  C:  HEME | aHMX = -1⋅26.7926 = -26.7916  HEME = -1  HMX = 1⋅26.7916 = 26.7916 |
| 060 | MAISA  Metallation of aISA by ISU. This is the reductive coupling of two [Fe_2_S_2_] clusters, donated by ISU, and installed as an [Fe_4_S_4_] cluster in ISA. | aISA + 0.9416092⋅ISU + 1.787040⋅NAHM → ISA + 0.9416091⋅aISU + 1.787040⋅NADM    Carbon: 6313.5 + 0.9416092⋅(12913.5) + 1.787040⋅(10) → 6313.5 + 0.9416091⋅(12913.5)+ 1.787040⋅(10)  Iron: 3.33922 + 0.9416092⋅(10.153688) + 1.787040⋅(0) → 10.487 + 0.9416091⋅(2.562258) + 1.787040⋅(0) | None | M:  aISA  ISU  NAHM  ISA  aISU  NADM | aISA = -1⋅6.43 = -6.43  ISU = -0.9416092⋅6.43 = -6.054547156  NAHM = -1.787040⋅6.43 = -11.4906672  ISA = +1⋅6.43 = +6.43  aISU = +0.9416091⋅6.43 = +6.054547156  NADM = +1.787040⋅6.43 = +11.4906672 |
| 061 | MAISU  Metallation of aISU by the mitochondrial Fe^II^ pool, forming a [Fe_2_S_2_] cluster. | aISU + 7.59143⋅FM → ISU    Carbon: 12913.5 + 7.59143⋅(0) → 12913.5  Iron: 2.562258+ 7.59143⋅(1) → 10.153688 | None | M:  aISU  FM  ISU | aISU = -1⋅6.43 = 6.43  FM = -7.59143⋅6.43 = -48.8128949  ISU = 1⋅6.43 = 6.43 |
| 062 | MALEU  Metallation of aLEU by the CIA and HEME. This installs an [Fe_4_S_4_] cluster in LEU as well as a small amount of heme. | aLEU + 2.092925⋅CIA +0.103964⋅HEME → LEU + 2.092925⋅aCIA + 2.92658⋅FC    Carbon: 34995.5 + 2.092925⋅(23339.5) +0.103964⋅(35) → 34999.1387+ 2.092925⋅(23339.5) + 2.92658⋅(0)  Iron: 0 + 2.092925⋅(22.5101) +0.103964⋅(1) → 36.58996 + 2.092925⋅(3.678776) + 2.92658⋅(1) | None | C:  aLEU  CIA  HEME  LEU  aCIA  FC | aLEU = -1  CIA = -2.0929  HEME = -0.10396  LEU = 1  aCIA = 2.09293  FC = 2.92658 |
| 063 | MALYS  Metallation of aLYS by ISA, ISU, and FM. This installs an [Fe_4_S_4_] cluster, an [Fe_2_S_2_] cluster, and a mono-iron center into aLYS. | aLYS + 0.3725193⋅ISA + 0.8127375⋅ISU + 0.249366⋅FM → LYS + 0.3725193⋅aISA + 0.8127375⋅aISU    Carbon: 11742 + 0.3725193⋅(6313.5) + 0.8127375⋅(12913.5) + 0.249366⋅(0) → 11742 + 0.3725193⋅(6313.5) + 0.8127375⋅(12913.5)  Iron: 0 + 0.3725193⋅(10.487) + 0.8127375⋅(10.153688) + 0.249366⋅(1) → 9.082034 + 0.3725193⋅(3.33922) + 0.8127375⋅(2.562258) | None | M:  LYS  ISA  ISU  FM  aISA  aISU  C:  aLYS | aLYS = -1  ISA = -0.3725193⋅6.43 = -2.395299  ISU = -0.8127375⋅6.43 = -5.225902  FM = -0.249366⋅6.43 = -1.603423  LYS = 1⋅6.43 = 6.43  aISA = 0.3725193⋅6.43 = 2.3952991  aISU = 0.8127375⋅6.43 = 5.2259021 |
| 064 | MAMEM  Metallation of aMEM by HEME and the cytosolic labile Fe^II^ pool. This installs heme and mono-iron centers into aMEM. | aMEM + 8.222864⋅HEME + 15.475069⋅FC → MEM    Carbon: 26113 + 8.222864⋅(35) + 15.475069⋅(0) → 26400.80  Iron: 0 + 8.222864⋅(1) + 15.475069⋅(1) → 23.69787 | None | E:  MEM  C:  aMEM  HEME  FC | aMEM = -1  HEME = -8.222864  FC = -15.47507  MEM = 26.7916 |
| 065 | MANUC  Metallation of aNUC by the CIA and the cytosolic labile Fe^II^ pool. | aNUC + 0.16063326⋅CIA + 2.512572⋅FC → NUC + 0.16063326⋅aCIA    Carbon: 9464 + 0.16063326⋅(23339.5) + 2.512572⋅(0) → 9464 + 0.16063326⋅(23339.5)  Iron: 0 + 0.16063326⋅(22.510144) + 2.512572⋅(1) → 5.537516 + 0.16063326⋅(3.678776) | None | C:  aNUC  CIA  FC  NUC  aCIA | aNUC = -1  CIA = -0.1606333  FC = -2.512572  NUC = 1  aCIA = 0.1606333 |
| 066 | MAPOL  Metallation of aPOL by the CIA. | aPOL + 1.6447644⋅CIA + 3.256706⋅FC → POL + 1.6447644⋅aCIA    Carbon: 58273.5 + 1.6447644⋅(23339.5) + 3.256706⋅(0) → 58273.5 + 1.6447644⋅(23339.5)  Iron: 0 + 1.6447644⋅(22.510144) + 3.256706⋅(1) → 34.22987 + 1.6447644⋅(3.678776) | None | N:  POL  C:  aPOL  CIA  FC  aCIA | aPOL = -1  CIA = -1.644764  FC = -3.256706  POL = 1⋅4.2583 = 4.2583  aCIA = 1.6447644 |
| 067 | MARIB  Metallation of aRIB by the CIA. | aRIB + 1.650608⋅CIA + 3.784381⋅FC → RIB + 1.650608⋅aCIA    Carbon: 24078.5+ 1.650608⋅( 23339.5) + 3.784381⋅(0) → 24078.5 + 1.650608⋅(23339.5)  Iron: 0 + 1.650608⋅(22.510144) + 3.784381⋅(1) → 34.8667 + 1.650608⋅(3.678776) | None | C:  aRIB  CIA  FC  RIB  aCIA | aRIB = -1  CIA = -1.6506  FC = -3.7844  RIB = 1  aCIA = 1.65061 |
| 068 | MATCA  Metallation of aTCA by ISA, ISU, and HEM. | aTCA + 4.0149197⋅ISA + 0.1939932⋅ISU + 0.047901255⋅HEM → TCA + 4.0149197⋅aISA + 0.1939932⋅aISU+0.736343⋅FM + 0.047901255⋅aHEM    Carbon: 22944.0 + 4.0149197⋅(6313.5) + 0.1939932⋅(12913.5) + 0.047901255⋅(15783.9) → 22955.827 + 4.0149197⋅(6313.5) + 0.1939932⋅(12913.5)+0.736343⋅(0) + 0.047901255⋅(15537)  Iron: 0 + 4.0149197⋅(10.487) + 0.1939932⋅(10.153688) + 0.047901255⋅(7.054408) → 29.773 + 4.0149197⋅(3.33922) + 0.1939932⋅(2.562258)+0.736343⋅(1) + 0.047901255⋅(0) | None | M:  TCA  ISA  ISU  HEM  aISA  aISU  FM  C:  aTCA  aHEM | aTCA = -1  ISA = -4.0149197⋅6.43 = -25.815934  ISU = -0.1939932⋅6.43 = -1.2473763  HEM = -0.047901255⋅6.43 = -0.3080051  TCA = 1⋅6.43 = 6.43  aISA = 4.0149197⋅6.43 = 25.815934  aISU = 0.1939932⋅6.43 = 1.2473763  FM = 0.736343⋅6.43 = 4.7346855  aHEM = 0.0479013 |
| 069 | MAYAP  Metallation of aYAP | aYAP + 0.1062058⋅CIA + 0.6080284⋅GRX → YAP + 0.1062058⋅aCIA + 0.6080284⋅aGRX    Carbon: 1225 + 0.1062058⋅(23339.5) + 0.6080284⋅(4864) → 1225 + 0.1062058⋅(23339.5) + 0.6080284⋅(4864)  Iron: 0 + 0.1062058⋅(22.510144) + 0.6080284⋅(4.93398) → 5.00 + 0.1062058⋅(3.678776) + 0.6080284⋅(0) |  | N:  YAP  C:  aYAP  CIA  GRX  aCIA  aGRX | aYAP -1  CIA = -0.1062058  GRX = -0.6080284  YAP = 1⋅4.1258 = 4.2583  aCIA = 0.1062058  aGRX = 0.6080284 |
| 070 | TAAM  Transfer of the amino acid AA from mitochondria to cytosol. | AAM → AA  Carbon: 5 → 5  Iron: 0 → 0 | None | M:  AAM  C:  AA | AAM = -6.43  AA = 1 |
| 071 | TACA  Transfer of acetyl-CoA from cytosol to mitochondria. | ACAC→ACA  Carbon: 5 → 5  Iron: 0 → 0 | None | M:  ACA  C:  ACAC | ACAC = -1  ACA = 6.43 |
| 072 | TADP  Transfer of ADP from cytosol into mitochondria. | ADP → ADPM  Carbon: 10 → 10  Iron: 0 → 0 | None | M:  ADPM  C:  ADP | ADP = -1  ADPM = 6.43 |
| 073 | TATPM  Transfer of ATP from mitochondria to cytosol. | ATPM → ATP  Carbon: 10 → 10  Iron: 0 → 0 | None | M:  ATPM  C:  ATP | ATPM = -6.43  ATP = 1 |
| 074 | TNADM  Transfer of NAD from cytosol to mitochondria. | NAD → NADM  Carbon: 10 → 10  Iron: 0 → 0 | None | M:  NADM  C:  NAD | NAD = -1  NADM = 6.43 |
| 075 | TNADV  Transfer of NAD from vacuole to cytosol. | NADV → NAD  Carbon: 10 → 10  Iron: 0 → 0 | None | V:  NADV  C:  NAD | NADV = -7.84146  NAD = 1 |
| 076 | TNAH  Transfer of NAH (NADH/NADPH) from cytosol to vacuole. | NAH → NAHV  Carbon: 10 → 10  Iron: 0 → 0 | None | V:  NADV  C:  NAH | NAH = -1  NAHV = 7.84146 |
| 077 | TNAHM  Transfer of NAH from mitochondria to cytosol. | NAHM → NAH  Carbon: 10 → 10  Iron: 0 → 0 | None | M:  NAHM  C:  NAH | NAHM = -6.43  NAH = 1 |
| 078 | TNUCM  Transfer of the nucleotide NUC from cytosol to nucleus. | NUCM → NUCMN  Carbon: 10 → 10  Iron: 0 → 0 | none; | N:  NUCMN  C:  NUCM | NUCM = -1  NUCMN = 4.2583 |
| 079 | TO2  Import of environmental O_2_ into the cytosol. | OXYGEN → O2  Carbon: 0 → 0  Iron: 0 → 0 | None | Env:  OXYGEN  C:  O2 | O2 = 1 |
| 080 | TO2M  Transfer of O_2_ from cytosol to mitochondria. | O2 → O2M  Carbon: 0 → 0  Iron: 0 → 0 | None | M:  O2M  C:  O2 | O2 = -1  O2M = 6.43 |
| 081 | TO2V  Transfer of O_2_ from cytosol to vacuole. | O2 → O2V  Carbon: 0 → 0  Iron: 0 → 0 | None | V:  O2V  C:  O2 | O2 = -1  O2V = 7.84146 |
| 082 | TROSE  Transfer of ROS from cytosol to ER | ROS → ROSE  Carbon: 0 → 0  Iron: 0 → 0 | None | E:  ROSE  C:  ROS | ROS = -1  ROSE = 26.7916 |
| 083 | TROSM  Transfer of ROS from mitochondria to cytosol. | ROSM → ROS  Carbon: 0 → 0  Iron: 0 → 0 | None | M:  ROSM  C:  ROX | ROSM = -6.43  ROS = 1 |
| 084 | TROSN  Transfer of ROS from cytosol to nucleus. | ROS → ROSN  Carbon: 0 → 0  Iron: 0 → 0 | None | N:  ROSN  C:  ROS | ROS = -1  ROSN = 4.2583 |
| 085 | TTCAM  Transfer of the TCA metabolite from mitochondria to cytosol. | TCAM → TCAMC  Carbon: 10 → 10  Iron: 0 → 0 | None | M:  TCAM  C:  TCAMC | TCAM = -6.43  TCAMC = 1 |
| 086 | UAFT  Demetallation of AFT by aGRX. | AFT + 0.3459033⋅aGRX → aAFT + 0.3459033⋅GRX    Carbon: 10989.0 + 0.3459033⋅(4864) → 10989.0+ 0.3459033⋅(4864)  Iron: 1.70668 + 0.3459033⋅(0) → 0 + 0.3459033⋅(4.93398) | None | C:  AFT  aGRX  aAFT  GRX | AFT = -1  aGRX = -0.3459  aAFT = 1  GRX = 0.3459 |
| 087 | UHEM  Release of free heme into cytosol as aHEM moves into the cytosol where it was biosynthesized. | HEM → aHEM + 7.054408⋅HEME  Carbon: 15783.9 → 15537 + 7.054408⋅(35)  Iron: 7.054408 → 0 + 7.054408⋅(1) | none | M:  HEM  C:  aHEM  HEME | HEM = -6.43  aHEM = 1  HEME = 7.054408 |
| 088 | UHMX  Demetallation of HMX | HMX + 3⋅O2 + 3⋅NAH → aHMX + FC + +3⋅NAD + 35⋅CO2  Carbon: 1620 + 3(0) + 3(10) → 1585 + 0 + +3(10) + 35⋅(1)  Iron: 1 + 3(0) + 3(0) → 0 + 1 + +3(0) + 35⋅(0) | aAFT | E:  HMX  aHMX  C:  O2  NAH  FC  NAD  CO2 | HMX = -1⋅26.792 = -26.792  O2 = -3  NAH = -3  aHMX = 1⋅26.7916 = 26.7916  FC = 1  NAD = 3  CO2 = 35 |
| 089 | UMEM  Demetallation of MEM under Fe-deficient conditions, generating the cytosolic Fe^II^ pool. | MEM + 8.222864⋅aHMX → aMEM + 8.222864⋅HMX +15.475069⋅FC    Carbon: 26400.80 + 8.222864⋅(1585) → 26113 + 8.222864⋅(1620) +15.475069⋅(0)  Iron: 23.69787 + 8.222864⋅(0) → 0 + 8.222864⋅(1) +15.475069⋅(1) | aAFT | E:  MEM  HMX  aHMX  C:  aMEM  FC | MEM = -1⋅26.792 = -26.792  aHMX = -8.222864⋅26.792 = -220.3  aMEM = 1  HMX = 8.222864⋅26.792 = 220.304  FC = 15.4751 |
| 090 | DAA  Dilution of AA. | AA → |  | C | AA = -1 |
| 091 | DAACIA  Dilution of aaCIA. | aaCIA → |  | C | aaCIA = -1 |
| 092 | DAAFT  Dilution of aAFT. | aAFT → |  | C | aAFT = -1 |
| 093 | DAAISA  Dilution of aaISA. | aaISA → |  | C | aaISA = -1 |
| 094 | DAAISU  Dilution of aaISU. | aaISU → |  | C | aaISU = -1 |
| 095 | DAAM  Dilution of AAM. | AAM → |  | M | AAM = -0.155521 |
| 096 | DACA  Dilution of ACA. | ACA → |  | M | ACA = -0.1555 |
| 097 | DACAC  Dilution of ACAC. | ACAC → |  | C | ACAC = -1 |
| 098 | DACAT  Dilution of aCAT. | aCAT → |  | C | aCAT = -1 |
| 099 | DACIA  Dilution of aCIA. | aCIA → |  | C | aCIA = -1 |
| 100 | DADP  Dilution of ADP. | ADP → |  | C | ADP = -1 |
| 101 | DADPM  Dilution of ADPM. | ADPM → |  | M | ADPM = -0.1555 |
| 102 | DAETC  Dilution of aETC. | aETC → |  | C | aETC = -1 |
| 103 | DAFT  Dilution of AFT. | AFT → |  | C | AFT = -1 |
| 104 | DAFT3  Dilution of aFT3. | aFT3 → |  | C | aFT3 = -1 |
| 105 | DAFT5  Dilution of aFT5. | aFT5 → |  | C | aFT5 = -1 |
| 106 | DAGRX  Dilution of aGRX. | aGRX → |  | C | aGRX = -1 |
| 107 | DAHEM  Dilution of aHEM. | aHEM → |  | C | aHEM = -1 |
| 108 | DAHMX  Dilution of aHMX. | aHMX → |  | E | aHMX = -0.0373 |
| 109 | DAISA  Dilution of aISA. | aISA → |  | M | aISA = -0.156 |
| 110 | DAISU  Dilution of aISU. | aISU → |  | M | aISU = -0.15552 |
| 111 | DALEU  Dilution of aLEU. | aLEU → |  | C | aLEU = -1 |
| 112 | DALYS  Dilution of aLYS. | aLYS → |  | C | aLYS = -1 |
| 113 | DAMEM  Dilution of aMEM. | aMEM → |  | C | aMEM = -1 |
| 114 | DANUC  Dilution of aNUC. | aNUC → |  | C | aNUC = -1 |
| 115 | DAPOL  Dilution of aPOL. | aPOL → |  | C | aPOL = -1 |
| 116 | DARIB  Dilution of aRIB. | aRIB → |  | C | aRIB = -1 |
| 117 | DATCA  Dilution of aTCA. | aTCA → |  | C | aTCA = -1 |
| 118 | DATM  Dilution of ATM. | ATM → |  | M | ATM = -0.1555 |
| 119 | DATP  Dilution of ATP. | ATP → |  | C | ATP = -1 |
| 120 | DATPM  Dilution of ATPM. | ATPM → |  | M | ATPM = -0.1555 |
| 121 | DAYAP  Dilution of aYAP. | aYAP → |  | C | aYAP = -1 |
| 122 | DCAT  Dilution of CAT. | CAT → |  | C | CAT = -1 |
| 123 | DCCC  Dilution of CCC | CCC → |  | V | CCC = -0.1275 |
| 124 | DCIA  Dilution of CIA. | CIA → |  | C | CIA = -1 |
| 125 | DCO2  Dilution of CO2. | CO2 → |  | C | CO2 = -1 |
| 126 | DCP  Dilution of CP. | CP → |  | C | CP = -1 |
| 127 | DDNA  Dilution of DNA. | DNA → |  | N | DNA = -0.2348 |
| 128 | DETC  Dilution of ETC. | ETC → |  | M | ETC = -0.1555 |
| 129 | DF2  Dilution of F2. | F2 → |  | V | F2 = -0.1275 |
| 130 | DF3  Dilution of F3. | F3 → |  | V | F3 = -0.1275 |
| 131 | DFC  Dilution of FC. | FC → |  | C | FC = -1 |
| 132 | DFT3  Dilution of FT3. | FT3 → |  | C | FT3 = -1 |
| 133 | DFT5  Dilution of FT5. | FT5 → |  | V | FT5 = -0.1275 |
| 134 | DFM  Dilution of FM. | FM → |  | M | FM = -0.1555 |
| 135 | DGRX  Dilution of GRX. | GRX → |  | C | GRX = -1 |
| 136 | DHEM  Dilution of HEM. | HEM → |  | M | HEM = -0.1555 |
| 137 | DHEME  Dilution of HEME. | HEME → |  | C | HEME = -1 |
| 138 | DHMX  Dilution of HMX. | HMX → |  | E | HMX = -0.0373 |
| 139 | DISA  Dilution of ISA. | ISA → |  | M | ISA = -0.1555 |
| 140 | DISU  Dilution of ISU. | ISU → |  | M | ISU = -0.1555 |
| 141 | DLEU  Dilution of LEU. | LEU → |  | C | LEU = -1 |
| 142 | DLYS  Dilution of LYS. | LYS → |  | M | LYS = -0.1555 |
| 143 | DMEM  Dilution of MEM. | MEM → |  | E | MEM = -0.0373 |
| 144 | DMP  Dilution of MP. | MP → |  | M | MP = -0.1555 |
| 145 | DMRS  Dilution of MRS. | MRS → |  | M | MRS = -0.1555 |
| 146 | DNAD  Dilution of NAD. | NAD → |  | C | NAD = -1 |
| 147 | DNADM  Dilution of NADM. | NADM → |  | M | NADM = -0.1555 |
| 148 | DNADV  Dilution of NADV. | NADV → |  | V | NADV = -0.1275 |
| 149 | DNAH  Dilution of NAH. | NAH → |  | C | NAH = -1 |
| 150 | DNAHM  Dilution of NAHM. | NAHM → |  | M | NAHM = -0.1555 |
| 151 | DNAHV  Dilution of NAHV. | NAHV → |  | V | NAHV = -0.1275 |
| 152 | DNUC  Dilution of NUC. | NUC → |  | C | NUC = -1 |
| 153 | DNUCM  Dilution of NUCM. | NUCM → |  | C | NUCM = -1 |
| 154 | DNUCMN  Dilution of NUCMN. | NUCMN → |  | N | NUCMN = -0.234837 |
| 155 | DO2  Dilution of O2. | O2 → |  | C | O2 = -1 |
| 156 | DO2M  Dilution of O2M | O2M → |  | M | O2M = -0.155521 |
| 157 | DO2V  Dilution of O2V. | O2V → |  | V | O2V = -0.1275273 |
| 158 | DPL  Dilution of PL. | PL → |  | E | PL = -0.037325 |
| 159 | DPOL  Dilution of POL. | POL → |  | N | POL = -0.2348 |
| 160 | DPRO  Dilution of PRO. | PRO → |  | C | PRO = -1 |
| 161 | DRIB  Dilution of RIB. | RIB → |  | C | RIB = -1 |
| 162 | DROS  Dilution of ROS. | ROS → |  | C | ROS = -1 |
| 163 | DROSE  Dilution of ROSE. | ROSE → |  | E | ROSE = -0.373 |
| 164 | DROSM  Dilution of ROSM. | ROSM → |  | M | ROSM = -0.1555 |
| 165 | DROSN  Dilution of ROSN. | ROSN → |  | N | ROSN = -0.2348 |
| 166 | DTCA  Dilution of TCA. | TCA → |  | M | TCA = -0.1555 |
| 167 | DTCAM  Dilution of TCAM. | TCAM → |  | M | TCAM = -0.1555 |
| 168 | DTCAMC  Dilution of TCAMC. | TCAMC → |  | C | TCAMC = -1 |
| 169 | DYAP  Dilution of YAP. | YAP → |  | N | YAP = -0.2348 |

**Table S10: Steady-State Reaction Rates used to generate the *U_ss(wt)_* state.** Reaction rates in µM/min in a yeast cell growing exponentially with α_cell_ = 0.003333 min^-1^. Whether rates are independent (I) or dependent (D) is indicated; 89 rates are I and 80 are D. Rate constants were obtained by equating each steady-state rate to the corresponding rate-law expression (Table S11) using *wt* conditions. For interregional reactions, the rates refer to those in the cytosol compartment. To obtain the rate in the non-cytosol compartment, multiply the listed rate by the volume ratio *V_cyt_*/*V_non-cyt_*.

| Number | Reaction Name | Rate (µM/min) | Dependent or Independent | Rate-constant Name | Rate-Multiplication factor | Rate-constant (µM/min) |
| --- | --- | --- | --- | --- | --- | --- |
| 001 | AATM | 6.597234 | D | kAATM | 8 | 52.77787 |
| 002 | ACAT | 500 | I | kACAT | 2 | 1000 |
| 003 | ACCC | 11.17875 | D | kACCC | 2 | 22.3575 |
| 004 | ACETC | 20770.9 | D | kACETC | 8 | 166167.2 |
| 005 | ACFT3 | 68.12587 | D | kACFT3 | 2 | 136.2517 |
| 006 | ACFT5 | 0 | I | kACFT5 | 2 | 0 |
| 007 | ACLEU | 5936.825 | D | kACLEU | 8 | 47494.6 |
| 008 | ACLYS | 523.4832 | D | kACLYS | 8 | 4187.865 |
| 009 | ACMEM | 2874.972 | D | kACMEM | 16 | 45999.54 |
| 010 | ACMRS | 56.0408 | D | kACMRS | 2 | 112.0816 |
| 011 | ACNUC | 620.303 | D | kACNUC | 4 | 2481.212 |
| 012 | ACPOL | 0.08 | D | kACPOL | 2 | 0.16 |
| 013 | ACTCA | 50506.97 | D | kACTCA | 8 | 404055.8 |
| 014 | AF2 | 7.154402 | D | kAF2 | 4 | 28.61761 |
| 015 | AF3 | 9.227554 | D | kAF3 | 4 | 36.91022 |
| 016 | AFC | 0 | D | kAFC | 4 | 0 |
| 017 | AFM | 0 | D | kAFM | 4 | 0 |
| 018 | AROSE | 25 | I | kROSE | 4 | 100 |
| 019 | AROSN | 0.074525 | I | kROSN | 4 | 0.298102 |
| 020 | ATCAA | 6484.759 | D | kATCAA | 2 | 12969.52 |
| 021 | BAACIA | 0.000807 | D | kBAACIA | 4 | 0.003227 |
| 022 | BAAFT | 0.00034 | D | kBAAFT | 4 | 0.001359 |
| 023 | BAAISA | 0.011975 | D | kBAAISA | 4 | 0.047901 |
| 024 | BAAISU | 0.035926 | D | kBAAISU | 4 | 0.143704 |
| 025 | BACAC | 460397.9 | D | kBACAC | 1 | 460397.9 |
| 026 | BACAT | 0.002366 | D | kBACAT | 4 | 0.009464 |
| 027 | BADP | 25.62115 | D | kBADP | 4 | 102.4846 |
| 028 | BAETC | 0.044211 | D | kBAETC | 4 | 0.176844 |
| 029 | BAFT3 | 0.000479 | D | kBAFT3 | 4 | 0.001915 |
| 030 | BAFT5 | 0.002981 | D | kBAFT5 | 4 | 0.011926 |
| 031 | BAGRX | 0.001002 | D | kBAGRX | 4 | 0.004008 |
| 032 | BAHEM | 0.00132 | D | kAHEM | 4 | 0.005279 |
| 033 | BAHMX | 0.016842 | D | kBAHMX | 4 | 0.067369 |
| 034 | BALEU | 0.004788 | D | kBALEU | 4 | 0.019151 |
| 035 | BALYS | 0.055223 | D | kBALYS | 4 | 0.220893 |
| 036 | BAMEM | 0.039897 | D | kBAMEM | 4 | 0.159588 |
| 037 | BANUC | 0.005742 | D | kBANUC | 4 | 0.022966 |
| 038 | BAPOL | 0.00211 | D | kBAPOL | 4 | 0.008441 |
| 039 | BARIB | 0.001308 | D | kBARIB | 4 | 0.005234 |
| 040 | BATCA | 0.125872 | D | kBATCA | 4 | 0.503487 |
| 041 | BATM | 0.005947 | D | kBATM | 4 | 0.023788 |
| 042 | BAYAP | 0.00028 | D | kBAYAP | 4 | 0.001122 |
| 043 | BCCC | 0.005824 | D | kBCCC | 4 | 0.023297 |
| 044 | BMRS | 0.00645 | D | kBMRS | 4 | 0.025802 |
| 045 | BNAD | 25.69173 | I | kBNAD | 4 | 102.7669 |
| 046 | BPRO | 0.000537 | D | kBPRO | 4 | 0.002149 |
| 047 | CATD | 0 | I | kCATD | 8 | 0 |
| 048 | ETCD | 0 | I | kETCD | 8 | 0 |
| 049 | MAACIA | 0.000726 | D | kMAACIA | 4 | 0.002905 |
| 050 | MAAFT | 0.00017 | D | kMAAFT | 4 | 0.00068 |
| 051 | MAAISA | 0.011772 | D | kMAAISA | 4 | 0.047088 |
| 052 | MAAISU | 0.035316 | D | kMAAISU | 8 | 0.282525 |
| 053 | MACAT | 0.00213 | D | kMACAT | 8 | 0.017036 |
| 054 | MACIA | 0.01554 | D | kMACIA | 8 | 0.124317 |
| 055 | MAETC | 0.04346 | D | kMAETC | 8 | 0.34768 |
| 056 | MAFT3 | 0.000239 | D | kMAFT3 | 4 | 0.000958 |
| 057 | MAFT5 | 0.002644 | D | kMAFT5 | 4 | 0.010577 |
| 058 | MAHEM | 0.138652 | D | kMAHEM | 32 | 4.436866 |
| 059 | MAHMX | 0.015158 | D | kMAHMX | 4 | 0.060631 |
| 060 | MAISA | 0.533198 | D | kMAISA | 8 | 4.265586 |
| 061 | MAISU | 7.219278 | D | kMAISU | 4 | 28.87711 |
| 062 | MALEU | 0.004309 | D | kMALEU | 8 | 0.034473 |
| 063 | MALYS | 0.054285 | D | kMALYS | 16 | 0.868562 |
| 064 | MAMEM | 0.039732 | D | kMAMEM | 8 | 0.317857 |
| 065 | MANUC | 0.005167 | D | kMANUC | 8 | 0.041339 |
| 066 | MAPOL | 0.002057 | D | kMAPOL | 8 | 0.016452 |
| 067 | MARIB | 0.001178 | D | kMARIB | 8 | 0.009421 |
| 068 | MATCA | 0.123734 | D | kMATCA | 16 | 1.979739 |
| 069 | MAYAP | 1.47E-05 | D | kMAYAP | 8 | 0.000117 |
| 070 | TAAM | 1012.051 | I | kTAAM | 2 | 2024.102 |
| 071 | TACA | 63476.76 | D | kTACA | 2 | 126953.5 |
| 072 | TADP | 133058.6 | D | kTADP | 2 | 266117.2 |
| 073 | TATPM | 133046.3 | D | kTATPM | 2 | 266092.6 |
| 074 | TNADM | 109456.4 | D | kTNADM | 2 | 218912.7 |
| 075 | TNADV | 0 | I | kTNADV | 2 | 0 |
| 076 | TNAH | 11.6442 | D | kTNAH | 2 | 23.28839 |
| 077 | TNAHM | 109450 | D | kTHAHM | 2 | 218900 |
| 078 | TNUCM | 620.1475 | D | kTNUCM | 2 | 1240.295 |
| 079 | TO2 | 32278.38 | D | kTO2 | 2 | 64556.76 |
| 080 | TO2M | 20770.91 | D | kTO2M | 2 | 41541.81 |
| 081 | TO2V | 7.327734 | D | kTO2V | 2 | 14.65547 |
| 082 | TROSE | 25.01667 | D | kTROSE | 2 | 50.03333 |
| 083 | TROSM | 600 | I | kTROSM | 2 | 1200 |
| 084 | TROSN | 0.091302 | D | kTROSN | 2 | 0.182605 |
| 085 | TTCAM | 5945.694 | D | kTTCAM | 2 | 11891.39 |
| 086 | UAFT | 0 | I | kUAFT | 4 | 0 |
| 087 | UHEM | 0.051056 | D | kUHEM | 2 | 0.102112 |
| 088 | UHMX | 0 | I | kUHMX | 8 | 0 |
| 089 | UMEM | 0 | I | kUMEM | 4 | 0 |
| 090 | DAA | 21.75645 | I | α_cell_ [AA] | N/A | α_cell_ = 0.003333 min^-1^ |
| 091 | DAACIA | 8.07E-05 | I | α_cell_ [AACIA] | N/A | ″ |
| 092 | DAAFT | 0.00017 | I | α_cell_ [AAFT] | N/A | ″ |
| 093 | DAAISA | 0.000203 | I | α_cell_ [AAISA] | N/A | ″ |
| 094 | DAAISU | 0.00061 | I | α_cell_ [AAISU] | N/A | ″ |
| 095 | DAAM | 32.63301 | I | α_cell_ [AAM] | N/A | ″ |
| 096 | DACA | 0.273331 | I | α_cell_ [ACA] | N/A | ″ |
| 097 | DACAC | 0.47589 | I | α_cell_ [ACAC] | N/A | ″ |
| 098 | DACAT | 0.000237 | I | α_cell_ [ACAT] | N/A | ″ |
| 099 | DACIA | 0.000363 | I | α_cell_ [ACIA] | N/A | ″ |
| 100 | DADP | 2.856374 | I | α_cell_ [ADP] | N/A | ″ |
| 101 | DADPM | 1.633317 | I | α_cell_ [ADPM] | N/A | ″ |
| 102 | DAETC | 0.000751 | I | α_cell_ [AETC] | N/A | ″ |
| 103 | DAFT | 0.00017 | I | α_cell_ [AFT] | N/A | ″ |
| 104 | DAFT3 | 0.000239 | I | α_cell_ [AFT3] | N/A | ″ |
| 105 | DAFT5 | 0.000337 | I | α_cell_ [AFT5] | N/A | ″ |
| 106 | DAGRX | 0.000519 | I | α_cell_ [AGRX] | N/A | ″ |
| 107 | DAHEM | 0.00066 | I | α_cell_ [AHEM] | N/A | ″ |
| 108 | DAHMX | 0.001685 | I | α_cell_ [AHMX] | N/A | ″ |
| 109 | DAISA | 0.005886 | I | α_cell_ [AISA] | N/A | ″ |
| 110 | DAISU | 0.017658 | I | α_cell_ [AISU] | N/A | ″ |
| 111 | DALEU | 0.000479 | I | α_cell_ [ALEU] | N/A | ″ |
| 112 | DALYS | 0.000938 | I | α_cell_ [ALYS] | N/A | ″ |
| 113 | DAMEM | 0.000165 | I | α_cell_ [AMEM] | N/A | ″ |
| 114 | DANUC | 0.000574 | I | α_cell_ [ANUC] | N/A | ″ |
| 115 | DAPOL | 5.37E-05 | I | α_cell_ [APOL] | N/A | ″ |
| 116 | DARIB | 0.000131 | I | α_cell_ [ARIB] | N/A | ″ |
| 117 | DATCA | 0.002138 | I | α_cell_ [ATCA] | N/A | ″ |
| 118 | DATM | 0.005947 | I | α_cell_ [ATM] | N/A | ″ |
| 119 | DATP | 10.4649 | I | α_cell_ [ATP] | N/A | ″ |
| 120 | DATPM | 10.66656 | D | α_cell_ [ATM] | N/A | ″ |
| 121 | DAYAP | 0.000266 | I | α_cell_ [AYAP] | N/A | ″ |
| 122 | DCAT | 0.00213 | I | α_cell_ [CAT] | N/A | ″ |
| 123 | DCCC | 0.005824 | I | α_cell_ [CCC] | N/A | ″ |
| 124 | DCIA | 0.000363 | I | α_cell_ [CIA] | N/A | ″ |
| 125 | DCO2 | 252534.9 | I | α_cell_ [CO2] | N/A | ″ |
| 126 | DCP | 0 | D | α_cell_ [CP] | N/A | ″ |
| 127 | DDNA | 0.005475 | I | α_cell_ [DNA] | N/A | ″ |
| 128 | DETC | 0.04346 | I | α_cell_ [ETC] | N/A | ″ |
| 129 | DF2 | 1.01625 | I | α_cell_ [F2] | N/A | ″ |
| 130 | DF3 | 10.1625 | I | α_cell_ [F3] | N/A | ″ |
| 131 | DFC | 0.279935 | I | α_cell_ [FC] | N/A | ″ |
| 132 | DFT3 | 0.000239 | I | α_cell_ [FT3] | N/A | ″ |
| 133 | DFT5 | 0.002644 | I | α_cell_ [FT5] | N/A | ″ |
| 134 | DFM | 0.299997 | I | α_cell_ [FM] | N/A | ″ |
| 135 | DGRX | 10.09039 | I | α_cell_ [GRX] | N/A | ″ |
| 136 | DHEM | 0.00066 | I | α_cell_ [HEM] | N/A | ″ |
| 137 | DHEME | 0.000156 | I | α_cell_ [HEME] | N/A | ″ |
| 138 | DHMX | 0.015158 | I | α_cell_ [HMX] | N/A | ″ |
| 139 | DISA | 0.005886 | I | α_cell_ [ISA] | N/A | ″ |
| 140 | DISU | 0.017658 | I | α_cell_ [ISU] | N/A | ″ |
| 141 | DLEU | 0.004309 | I | α_cell_ [LEU] | N/A | ″ |
| 142 | DLYS | 0.054285 | I | α_cell_ [LYS] | N/A | ″ |
| 143 | DMEM | 0.039732 | I | α_cell_ [MEM] | N/A | ″ |
| 144 | DMP | 0 | I | α_cell_ [MP] | N/A | ″ |
| 145 | DMRS | 0.00645 | I | α_cell_ [MRS] | N/A | ″ |
| 146 | DNAD | 5.202646 | D | α_cell_ [NAD] | N/A | ″ |
| 147 | DNADM | 2.66662 | I | α_cell_ [NADM] | N/A | ″ |
| 148 | DNADV | 9.227554 | I | α_cell_ [NADV] | N/A | ″ |
| 149 | DNAH | 2.471642 | I | α_cell_ [NAH] | N/A | ″ |
| 150 | DNAHM | 3.70663 | I | α_cell_ [NAHM] | N/A | ″ |
| 151 | DNAHV | 2.416643 | I | α_cell_ [NAHV] | N/A | ″ |
| 152 | DNUC | 0.005167 | I | α_cell_ [NUC] | N/A | ″ |
| 153 | DNUCM | 0.155519 | I | α_cell_ [NUCM] | N/A | ″ |
| 154 | DNUCMN | 0.147902 | I | α_cell_ [NUCMN] | N/A | ″ |
| 155 | DO2 | 0.259331 | I | α_cell_ [O2] | N/A | ″ |
| 156 | DO2M | 0.002 | I | α_cell_ [O2M] | N/A | ″ |
| 157 | DO2V | 0.173332 | I | α_cell_ [O2V] | N/A | ″ |
| 158 | DPL | 2849.965 | I | α_cell_ [PL] | N/A | ″ |
| 159 | DPOL | 0.002057 | I | α_cell_ [POL] | N/A | ″ |
| 160 | DPRO | 0.000537 | I | α_cell_ [PRO] | N/A | ″ |
| 161 | DRIB | 0.001178 | I | α_cell_ [RIB] | N/A | ″ |
| 162 | DROS | 74.89203 | I | α_cell_ [ROS] | N/A | ″ |
| 163 | DROSE | 0.016667 | I | α_cell_ [ROSE] | N/A | ″ |
| 164 | DROSM | 438.5452 | D | α_cell_ [ROSM] | N/A | ″ |
| 165 | DROSN | 0.016777 | I | α_cell_ [ROSN] | N/A | ″ |
| 166 | DTCA | 0.123734 | I | α_cell_ [TCA] | N/A | ″ |
| 167 | DTCAM | 13.29987 | I | α_cell_ [TCAM] | N/A | ″ |
| 168 | DTCAMC | 8.869245 | I | α_cell_ [TCAMC] | N/A | ″ |
| 169 | DYAP | 0.000266 | I | α_cell_ [YAP] | N/A | ″ |

**Table S11. Rate law expressions:**

| Reaction Number | Rate Law |
| --- | --- |
| 001 | $AATM=k_{AATM}\left(\frac{[ISU]}{[ISU]_{w}+[ISU]}\right)\left(\frac{[aGRX]}{[aGRX]_{w}+[aGRX]}\right)\left(\frac{[ATPM]}{[ATPM]_{w}+[ATPM]}\right)\left(\frac{[ATM]}{[ATM]_{W}}\right)\left(\frac{[PL]}{[PL]_{W}}\right)$ |
| 002 | $ACAT=k_{ACAT}\left(\frac{[ROS]}{[ROS]_{w}+[ROS]}\right)\left(\frac{[CAT]}{[CAT]_{W}}\right)$ |
| 003 | $ACCC=k_{ACCC}\left(\frac{[FC]}{[FC]_{w}+[FC]}\right)\left(\frac{[CCC]}{[CCC]_{W}}\right)\left(\frac{[PL]}{[PL]_{W}}\right)$ |
| 004 | $ACETC=k_{ACETC}\left(\frac{[NAHM]}{[NAHM]_{w}+[NAHM]}\right)\left(\frac{[O2M]}{[O2M]_{w}+[O2M]}\right)\left(\frac{[ADPM]}{[ADPM]_{w}+[ADPM]}\right)\left(\frac{[ETC]}{[ETC]_{W}}\right)\left(\frac{[PL]}{[PL]_{W}}\right)$ |
| 005 | $ACFT3=k_{ACFT3}\left(\frac{[IRON]}{[IRON]_{w}+[IRON]}\right)\left(\frac{[FT3]}{[FT3]_{W}}\right)\left(\frac{[PL]}{[PL]_{W}}\right)$ |
| 006 | $ACFT5=k_{ACFT5}\left(\frac{[F2]}{[F2]_{w}+[F2]}\right)\left(\frac{[FT5]}{[FT5]_{W}}\right)\left(\frac{[PL]}{[PL]_{W}}\right)$ |
| 007 | $ACLEU=k_{ACLEU}\left(\frac{[TCAMC]}{[TCAMC]_{w}+[TCAMC]}\right)\left(\frac{[ATP]}{[ATP]_{w}+[ATP]}\right)\left(\frac{[NAH]}{[NAH]_{w}+[NAH]}\right)\left(\frac{[LEU]}{[LEU]_{W}}\right)$ |
| 008 | $ACLYS=k_{ACLYS}\left(\frac{[TCAM]}{[TCAM]_{w}+[TCAM]}\right)\left(\frac{[ATPM]}{[ATPM]_{w}+[ATPM]}\right)\left(\frac{[NAHM]}{[NAHM]_{w}+[NAHM]}\right)\left(\frac{[LYS]}{[LYS]_{W}}\right)$ |
| 009 | $ACMEM=k_{ACMEM}\left(\frac{[ACAC]}{[ACAC]_{w}+[ACAC]}\right)\left(\frac{[ATP]}{[ATP]_{w}+[ATP]}\right)\left(\frac{[NAH]}{[NAH]_{w}+[NAH]}\right)\left(\frac{[O2]}{[O2]_{w}+[O2]}\right)\left(\frac{[MEM]}{[MEM]_{W}}\right)$ |
| 010 | $ACMRS=k_{ACMRS}\left(\frac{[FC]}{[FC]_{w}+[FC]}\right)\left(\frac{[MRS]}{[MRS]_{W}}\right)\left(\frac{[PL]}{[PL]_{W}}\right)\left(\frac{[ETC]}{[ETC]_{W}}\right)$ |
| 011 | $ACNUC=k_{ACNUC}\left(\frac{[AA]}{[AA]_{w}+[AA]}\right)\left(\frac{[ATP]}{[ATP]_{w}+[ATP]}\right)\left(\frac{[NUC]}{[NUC]_{W}}\right)$ |
| 012 | $ACPOL=k_{ACPOL}\left(\frac{[NUCMN]}{[NUCMN]_{w}+[NUCMN]}\right)\left(\frac{[POL]}{[POL]_{W}}\right)\left(\frac{[DNA]}{[DNA]_{W}}\right)$ |
| 013 | $ACTCA=k_{ACTCA}\left(\frac{[ACA]}{[ACA]_{w}+[ACA]}\right)\left(\frac{[NADM]}{[NADM]_{w}+[NADM]}\right)\left(\frac{[ADPM]}{[ADPM]_{w}+[ADPM]}\right)\left(\frac{[TCA]}{[TCA]_{W}}\right)\left(\frac{[TCAM]}{[TCAM]_{W}}\right)$ |
| 014 | $AF2=k_{AF2}\left(\frac{[F2]}{[F2]_{w}+[F2]}\right)\left(\frac{[O2V]}{[O2V]_{w}+[O2V]}\right)$ |
| 015 | $AF3=k_{AF3}\left(\frac{[F3]}{[F3]_{w}+[F3]}\right)\left(\frac{[NAHV]}{[NAHV]_{w}+[NAHV]}\right)$ |
| 016 | $AFC=k_{AFC}\left(\frac{[FC]}{[FC]_{w}+[FC]}\right)\left(\frac{[O2]}{[O2]_{w}+[O2]}\right)$ |
| 017 | $AFM=k_{AFM}\left(\frac{[FM]}{[FM]_{w}+[FM]}\right)\left(\frac{[O2M]}{[O2M]_{w}+[O2M]}\right)$ |
| 018 | $AROSE=k_{AROSE}\left(\frac{[PL]}{[PL_{w}+[PL]}\right)\left(\frac{[ROSE]}{[ROSE]_{w}+[ROSE]}\right)$ |
| 019 | $AROSN=k_{AROSN}\left(\frac{[DNA]}{[DNA]_{w}+[DNA]}\right)\left(\frac{[ROSN]}{[ROSN]_{w}+[ROSN]}\right)$ |
| 020 | $ATCAA=k_{ATCAA}\left(\frac{[ACA]}{[ACA]_{w}+[ACA]}\right)$ |
| 021 | $BAACIA=k_{BAACIA}\left(\frac{[AA]}{[AA]_{w}+[AA]}\right)\left(\frac{[ATP]}{[ATP]_{w}+[ATP]}\right)\left(\frac{[DNA]}{[DNA]_{W}}\right)\left(\frac{[RIB]}{[RIB]_{W}}\right)$ |
| 022 | $BAAFT=k_{BAAFT}\left(\frac{[AA]}{[AA]_{w}+[AA]}\right)\left(\frac{[ATP]}{[ATP]_{w}+[ATP]}\right)\left(\frac{[DNA]}{[DNA]_{W}}\right)\left(\frac{[RIB]}{[RIB]_{W}}\right)$  Modified to   |
| 023 | $BAAISA=k_{BAAISA}\left(\frac{[AA]}{[AA]_{w}+[AA]}\right)\left(\frac{[ATP]}{[ATP]_{w}+[ATP]}\right)\left(\frac{[DNA]}{[DNA]_{W}}\right)\left(\frac{[RIB]}{[RIB]_{W}}\right)$ |
| 024 | $BAAISU=k_{BAAISU}\left(\frac{[AA]}{[AA]_{w}+[AA]}\right)\left(\frac{[ATP]}{[ATP]_{w}+[ATP]}\right)\left(\frac{[DNA]}{[DNA]_{W}}\right)\left(\frac{[RIB]}{[RIB]_{W}}\right)$ |
| 025 | $BACAC=k_{BACAC}$ |
| 026 | $BACAT=k_{BACAT}\left(\frac{[AA]}{[AA]_{w}+[AA]}\right)\left(\frac{[ATP]}{[ATP]_{w}+[ATP]}\right)\left(\frac{[DNA]}{[DNA]_{W}}\right)\left(\frac{[RIB]}{[RIB]_{W}}\right)\left(\frac{[ROS]}{[ROS]_{W}}\right)$ |
| 027 | $BADP=k_{BADP}\left(\frac{[ACAC]}{[ACAC]_{w}+[ACAC]}\right)\left(\frac{[ATP]}{[ATP]_{w}+[ATP]}\right)$ |
| 028 | $BAETC=k_{BAETC}\left(\frac{[AA]}{[AA]_{w}+[AA]}\right)\left(\frac{[ATP]}{[ATP]_{w}+[ATP]}\right)\left(\frac{[DNA]}{[DNA]_{W}}\right)\left(\frac{[RIB]}{[RIB]_{W}}\right)\left(\frac{[HEM]}{[HEM]_{W}}\right)$  Modified to   |
| 029 | $BAFT3=k_{BAFT3}\left(\frac{[AA]}{[AA]_{w}+[AA]}\right)\left(\frac{[ATP]}{[ATP]_{w}+[ATP]}\right)\left(\frac{[DNA]}{[DNA]_{W}}\right)\left(\frac{[RIB]}{[RIB]_{W}}\right)\left(\frac{[PL]}{[PL]_{W}}\right)\left(\frac{[aAFT]}{[aAFT]_{W}}\right)$ |
| 030 | $BAFT5=k_{BAFT5}\left(\frac{[AA]}{[AA]_{w}+[AA]}\right)\left(\frac{[ATP]}{[ATP]_{w}+[ATP]}\right)\left(\frac{[DNA]}{[DNA]_{W}}\right)\left(\frac{[RIB]}{[RIB]_{W}}\right)\left(\frac{[PL]}{[PL]_{W}}\right)\left(\frac{[aAFT]}{[aAFT]_{W}}\right)$  Modified to   |
| 031 | $BAGRX=k_{BAGRX}\left(\frac{[AA]}{[AA]_{w}+[AA]}\right)\left(\frac{[ATP]}{[ATP]_{w}+[ATP]}\right)\left(\frac{[DNA]}{[DNA]_{W}}\right)\left(\frac{[RIB]}{[RIB]_{W}}\right)\left(\frac{[YAP]}{[YAP]_{W}}\right)$  Modified to   |
| 032 | $BAHEM=k_{BAHEM}\left(\frac{[AA]}{[AA]_{w}+[AA]}\right)\left(\frac{[ATP]}{[ATP]_{w}+[ATP]}\right)\left(\frac{[DNA]}{[DNA]_{W}}\right)\left(\frac{[RIB]}{[RIB]_{W}}\right)$ |
| 033 | $BAHMX=k_{BAHMX}\left(\frac{[AA]}{[AA]_{w}+[AA]}\right)\left(\frac{[ATP]}{[ATP]_{w}+[ATP]}\right)\left(\frac{[DNA]}{[DNA]_{W}}\right)\left(\frac{[RIB]}{[RIB]_{W}}\right)\left(\frac{[aAFT]}{[aAFT]_{W}}\right)$  Modified to   |
| 034 | $BALEU=k_{BALEU}\left(\frac{[AA]}{[AA]_{w}+[AA]}\right)\left(\frac{[ATP]}{[ATP]_{w}+[ATP]}\right)\left(\frac{[DNA]}{[DNA]_{W}}\right)\left(\frac{[RIB]}{[RIB]_{W}}\right)$ |
| 035 | $BALYS=k_{BALYS}\left(\frac{[AA]}{[AA]_{w}+[AA]}\right)\left(\frac{[ATP]}{[ATP]_{w}+[ATP]}\right)\left(\frac{[DNA]}{[DNA]_{W}}\right)\left(\frac{[RIB]}{[RIB]_{W}}\right)$ |
| 036 | $BAMEM=k_{BAMEM}\left(\frac{[AA]}{[AA]_{w}+[AA]}\right)\left(\frac{[ATP]}{[ATP]_{w}+[ATP]}\right)\left(\frac{[DNA]}{[DNA]_{W}}\right)\left(\frac{[PL]}{[PL]_{W}}\right)$ |
| 037 | $BANUC=k_{BANUC}\left(\frac{[AA]}{[AA]_{w}+[AA]}\right)\left(\frac{[ATP]}{[ATP]_{w}+[ATP]}\right)\left(\frac{[DNA]}{[DNA]_{W}}\right)\left(\frac{[RIB]}{[RIB]_{W}}\right)$ |
| 038 | $BAPOL=k_{BAPOL}\left(\frac{[AA]}{[AA]_{w}+[AA]}\right)\left(\frac{[ATP]}{[ATP]_{w}+[ATP]}\right)\left(\frac{[DNA]}{[DNA]_{W}}\right)\left(\frac{[RIB]}{[RIB]_{W}}\right)$ |
| 039 | $BARIB=k_{BARIB}\left(\frac{[AA]}{[AA]_{w}+[AA]}\right)\left(\frac{[ATP]}{[ATP]_{w}+[ATP]}\right)\left(\frac{[DNA]}{[DNA]_{W}}\right)\left(\frac{[RIB]}{[RIB]_{W}}\right)$ |
| 040 | $BATCA=k_{BATCA}\left(\frac{[AA]}{[AA]_{w}+[AA]}\right)\left(\frac{[ATP]}{[ATP]_{w}+[ATP]}\right)\left(\frac{[DNA]}{[DNA]_{W}}\right)\left(\frac{[RIB]}{[RIB]_{W}}\right)$ |
| 041 | $BATM=k_{BATM}\left(\frac{[AA]}{[AA]_{w}+[AA]}\right)\left(\frac{[ATP]}{[ATP]_{w}+[ATP]}\right)\left(\frac{[DNA]}{[DNA]_{W}}\right)\left(\frac{[RIB]}{[RIB]_{W}}\right)\left(\frac{[PL]}{[PL]_{W}}\right)$ |
| 042 | $BAYAP=k_{BAYAP}\left(\frac{[AA]}{[AA]_{w}+[AA]}\right)\left(\frac{[ATP]}{[ATP]_{w}+[ATP]}\right)\left(\frac{[DNA]}{[DNA]_{W}}\right)\left(\frac{[RIB]}{[RIB]_{W}}\right)$ |
| 043 | $BCCC=k_{BCCC}\left(\frac{[AA]}{[AA]_{w}+[AA]}\right)\left(\frac{[ATP]}{[ATP]_{w}+[ATP]}\right)\left(\frac{[DNA]}{[DNA]_{W}}\right)\left(\frac{[RIB]}{[RIB]_{W}}\right)\left(\frac{[PL]}{[PL]_{W}}\right)\left(\frac{[YAP]}{[YAP]_{W}}\right)$ |
| 044 | $BMRS=k_{BMRS}\left(\frac{[AA]}{[AA]_{w}+[AA]}\right)\left(\frac{[ATP]}{[ATP]_{w}+[ATP]}\right)\left(\frac{[DNA]}{[DNA]_{W}}\right)\left(\frac{[RIB]}{[RIB]_{W}}\right)\left(\frac{[PL]}{[PL]_{W}}\right)$  Modified to   |
| 045 | $BNAD=k_{BNAD}\left(\frac{[ACAC]}{[ACAC]_{w}+[ACAC]}\right)\left(\frac{[ATP]}{[ATP]_{w}+[ATP]}\right)$ |
| 046 | $BPRO=k_{BPRO}\left(\frac{[AA]}{[AA]_{w}+[AA]}\right)\left(\frac{[ATP]}{[ATP]_{w}+[ATP]}\right)\left(\frac{[DNA]}{[DNA]_{W}}\right)\left(\frac{[RIB]}{[RIB]_{W}}\right)$ |
| 047 | $CATD=k_{CATD}\left(\frac{[CAT]}{K_{CAT}+[CAT]}\right)\left(\frac{[O2]}{K_{O2}+[O2]}\right)\left(\frac{[NAH]}{K_{NAH}+[NAH]}\right)\left(\frac{[HMX]}{[HMX]_{W}}\right)\left(\frac{[aAFT]}{[aAFT]_{W}}\right)$ |
| 048 | $ETCD=k_{ETCS}\left(\frac{[ETC]}{[ETC]_{w}+[ETC]}\right)\left(\frac{[O2]}{[O2]_{w}+[O2]}\right)\left(\frac{[NAH]}{[NAH]_{w}+[NAH]}\right)\left(\frac{[HMX]}{[HMX]_{W}}\right)\left(\frac{[aAFT]}{[aAFT]_{W}}\right)$ |
| 049 | $MAACIA=k_{MAACIA}\left(\frac{[aaCIA]}{[aaCIA]_{w}+[aaCIA]}\right)\left(\frac{[GRX]}{[GRX]_{w}+[GRX]}\right)$ |
| 050 | $MAAFT=k_{MAAFT}\left(\frac{[aAFT]}{[aAFT]_{w}+[aAFT]}\right)\left(\frac{[GRX]}{[GRX]_{w}+[GRX]}\right)$ |
| 051 | $MAAISA=k_{MAAISA}\left(\frac{[aaISA]}{[aaISA]_{w}+[aaISA]}\right)\left(\frac{[ISU]}{[ISU]_{w}+[ISU]}\right)$ |
| 052 | $MAAISU=k_{MAAISU}\left(\frac{[aaISU]}{[aaISU]_{w}+[aaISU]}\right)\left(\frac{[ISA]}{[ISA]_{w}+[ISA]}\right)\left(\frac{[FM]}{[FM]_{w}+[FM]}\right)$ |
| 053 | $MACAT=k_{MACAT}\left(\frac{[aCAT]}{[aCAT]_{w}+[aCAT]}\right)\left(\frac{[HEME]}{[HEME]_{w}+[HEME]}\right)\left(\frac{[GRX]}{[GRX]_{w}+[GRX]}\right)$ |
| 054 | $MACIA=k_{MACIA}\left(\frac{[aCIA]}{[aCIA]_{w}+[aCIA]}\right)\left(\frac{[GRX]}{[GRX]_{w}+[GRX]}\right)\left(\frac{[NAH]}{[NAH]_{w}+[NAH]}\right)$ |
| 055 | $MAETC=k_{MAETC}\left(\frac{[aETC]}{[aETC]_{w}+[aETC]}\right)\left(\frac{[HEM]}{[HEM]_{w}+[HEM]}\right)\left(\frac{[ISU]}{[ISU]_{w}+[ISU]}\right)\left(\frac{[PL]}{[PL]_{w}}\right)$ |
| 056 | $MAFT3=k_{MAFT3}\left(\frac{[aFT3]}{[aFT3]_{w}+[aFT3]}\right)\left(\frac{[HEME]}{[HEME]_{w}+[HEME]}\right)\left(\frac{[PL]}{[PL]_{w}}\right)$ |
| 057 | $MAFT5=k_{MAFT5}\left(\frac{[aFT5]}{[aFT5]_{w}+[aFT5]}\right)\left(\frac{[HEME]}{[HEME]_{w}+[HEME]}\right)\left(\frac{[PL]}{[PL]_{w}}\right)$ |
| 058 | $MAHEM=k_{MAHEM}\left(\frac{[aHEM]}{[aHEM]_{w}+[aHEM]}\right)\left(\frac{[FM]}{[FM]_{w}+[FM]}\right)\left(\frac{[TCAM]}{[TCAM]_{w}+[TCAM]}\right)\left(\frac{[AAM]}{[AAM]_{w}+[AAM]}\right)\left(\frac{[NAHM]}{[NAHM]_{w}+[NAHM]}\right)$ |
| 059 | $MAHMX=k_{MAHMX}\left(\frac{[aHMX]}{[aHMX]_{w}+[aHMX]}\right)\left(\frac{[HEME]}{[HEME]_{w}+[HEME]}\right)$ |
| 060 | $MAISA=k_{MAISA}\left(\frac{[aISA]}{[aISA]_{w}+[aISA]}\right)\left(\frac{[ISU]}{[ISU]_{w}+[ISU]}\right)\left(\frac{[NAHM]}{[NAHM]_{w}+[NAHM]}\right)$ |
| 061 | $MAISU=k_{MAISU}\left(\frac{[aISU]}{[aISU]_{w}+[aISU]}\right)\left(\frac{[FM]}{[FM]_{w}+[FM]}\right)$ |
| 062 | $MALEU=k_{MALEU}\left(\frac{[aLEU]}{[aLEU]_{w}+[aLEU]}\right)\left(\frac{[CIA]}{[CIA]_{w}+[CIA]}\right)\left(\frac{[HEME]}{[HEME]_{w}+[HEME]}\right)$ |
| 063 | $MALYS=k_{MALYS}\left(\frac{[aLYS]}{[aLYS]_{w}+[aLYS]}\right)\left(\frac{[ISA]}{[ISA]_{w}+[ISA]}\right)\left(\frac{[ISU]}{[ISU]_{w}+[ISU]}\right)\left(\frac{[FM]}{[FM]_{w}+[FM]}\right)$ |
| 064 | $MAMEM=k_{MAMEM}\left(\frac{[aMEM]}{[aMEM]_{w}+[aMEM]}\right)\left(\frac{[HEME]}{[HEME]_{w}+[HEME]}\right)\left(\frac{[FC]}{[FC]_{w}+[FC]}\right)$ |
| 065 | $MANUC=k_{MANUC}\left(\frac{[aNUC]}{[aNUC]_{w}+[aNUC]}\right)\left(\frac{[CIA]}{[CIA]_{w}+[CIA]}\right)\left(\frac{[FC]}{[FC]_{w}+[FC]}\right)$ |
| 066 | $MAPOL=k_{MAPOL}\left(\frac{[aPOL]}{[aPOL]_{w}+[aPOL]}\right)\left(\frac{[CIA]}{[CIA]_{w}+[CIA]}\right)\left(\frac{[FC]}{[FC]_{w}+[FC]}\right)$ |
| 067 | $MARIB=k_{MARIB}\left(\frac{[aRIB]}{[aRIB]_{w}+[aRIB]}\right)\left(\frac{[CIA]}{[CIA]_{w}+[CIA]}\right)\left(\frac{[FC]}{[FC]_{w}+[FC]}\right)$ |
| 068 | $MATCA=k_{MATCA}\left(\frac{[aTCA]}{[aTCA]_{w}+[aTCA]}\right)\left(\frac{[ISA]}{[ISA]_{w}+[ISA]}\right)\left(\frac{[ISU]}{[ISU]_{w}+[ISU]}\right)\left(\frac{[HEM]}{[HEM]_{w}+[HEM]}\right)$ |
| 069 | $MAYAP=k_{MAYAP}\left(\frac{[aYAP]}{[aYAP]_{w}+[aYAP]}\right)\left(\frac{[CIA]}{[CIA]_{w}+[CIA]}\right)\left(\frac{[GRX]}{[GRX]_{w}+[GRX]}\right)$ |
| 070 | $TAAM=k_{TAAM}\left(\frac{[AAM]}{[AA]}-1\right)$ |
| 071 | $TACA=k_{TACA}\left(\frac{[ACAC]}{[ACA]}-1\right)$ |
| 072 | $TADP=k_{TADP}\left(\frac{[ADP]}{[ADPM]}-1\right)$ |
| 073 | $TATPM=k_{TATPM}\left(\frac{[ATPM]}{[ATP]}-1\right)$ |
| 074 | $TNADM=k_{TNADM}\left(\frac{[NAD]}{[NADM]}-1\right)$ |
| 075 | $TNADV=k_{TNADV}\left(\frac{[NADV]}{[NAD]}-1\right)$ |
| 076 | $TNAH=k_{TNAH}\left(\frac{[NAH]}{[NAHV]}-1\right)$ |
| 077 | $TNAHM=k_{TNAHM}\left(\frac{[NAHM]}{[NAH]}-1\right)$ |
| 078 | $TNUCM=k_{TNUCM}\left(\frac{[NUCM]}{[NUCMN]}-1\right)$ |
| 079 | $TO2=k_{TO2}\left(\frac{[OXYGEN]}{[O2]}-1\right)$ |
| 080 | $TO2M=k_{TO2M}\left(\frac{[O2]}{[O2M]}-1\right)$ |
| 081 | $TO2V=k_{TO2V}\left(\frac{[O2]}{[O2V]}-1\right)$ |
| 082 | $TROSE=k_{TROSE}\left(\frac{[ROS]}{[ROSE]}-1\right)$ |
| 083 | $TROSM=k_{TROSM}\left(\frac{[ROSM]}{[ROS]}-1\right)$ |
| 084 | $TROSN=k_{TROSN}\left(\frac{[ROS]}{[ROSN]}-1\right)$ |
| 085 | $TTCAM=k_{TTCAM}\left(\frac{[TCAM]}{[TCAMC]}-1\right)$ |
| 086 | $UAFT=k_{UAFT}\left(\frac{[AFT]}{[AFT]_{w}+[AFT]}\right)\left(\frac{[aGRX]}{[aGRX]_{w}+[aGRX]}\right)$ |
| 087 | $UHEM=k_{UHEM}\left(\frac{[HEM]}{[HEM]_{w}+[HEM]}\right)$ |
| 088 | $UHMX=k_{UHMX}\left(\frac{[HMX]}{[HMX]_{w}+[HMX]}\right)\left(\frac{[O2]}{[O2]_{w}+[O2]}\right)\left(\frac{[NAH]}{[NAH]_{w}+[NAH]}\right)\left(\frac{[aAFT]}{[aAFT]_{w}}\right)$ |
| 089 | $UMEM=k_{UMEM}\left(\frac{[MEM]}{[MEM]_{w}+[MEM]}\right)\left(\frac{[aHMX]}{[aHMX]_{w}+[aHMX]}\right)\left(\frac{[aAFT]}{[aAFT]_{w}}\right)$ |
| 090 | $DAA=\alpha_{cell}\cdot[AA]$ |
| 091 | $DAACIA=\alpha_{cell}\cdot[aaCIA]$ |
| 092 | $DAAFT=\alpha_{cell}\cdot[aAFT]$ |
| 093 | $DAAISA=\alpha_{cell}\cdot[aaISA]$ |
| 094 | $DAAISU=\alpha_{cell}\cdot[aaISU]$ |
| 095 | $DAAM=\alpha_{cell}\cdot[AAM]$ |
| 096 | $DACA=\alpha_{cell}\cdot[ACA]$ |
| 097 | $DACAC=\alpha_{cell}\cdot[ACAC]$ |
| 098 | $DACAT=\alpha_{cell}\cdot[aCAT]$ |
| 099 | $DACIA=\alpha_{cell}\cdot[aCIA]$ |
| 100 | $DADP=\alpha_{cell}\cdot[ADP]$ |
| 101 | $DADPM=\alpha_{cell}\cdot[ADPM]$ |
| 102 | $DAETC=\alpha_{cell}\cdot[aETC]$ |
| 103 | $DAFT=\alpha_{cell}\cdot[AFT]$ |
| 104 | $DAF3=\alpha_{cell}\cdot[aFT3]$ |
| 105 | $DAF5=\alpha_{cell}\cdot[aFT5]$ |
| 106 | $DAGRX=\alpha_{cell}\cdot[aGRX]$ |
| 107 | $DAHEM=\alpha_{cell}\cdot[aHEM]$ |
| 108 | $DAHMX=\alpha_{cell}\cdot[aHMX]$ |
| 109 | $DAISA=\alpha_{cell}\cdot[aISA]$ |
| 110 | $DAISU=\alpha_{cell}\cdot[aISU]$ |
| 111 | $DALEU=\alpha_{cell}\cdot[aLEU]$ |
| 112 | $DALYS=\alpha_{cell}\cdot[aLYS]$ |
| 113 | $DAMEM=\alpha_{cell}\cdot[aMEM]$ |
| 114 | $DANUC=\alpha_{cell}\cdot[aNUC]$ |
| 115 | $DAPOL=\alpha_{cell}\cdot[aPOL]$ |
| 116 | $DARIB=\alpha_{cell}\cdot[aRIB]$ |
| 117 | $DATCA=\alpha_{cell}\cdot[aTCA]$ |
| 118 | $DATM=\alpha_{cell}\cdot[ATM]$ |
| 119 | $DATP=\alpha_{cell}\cdot[ATP]$ |
| 120 | $DATPM=\alpha_{cell}\cdot[ATPM]$ |
| 121 | $DAYAP=\alpha_{cell}\cdot[aYAP]$ |
| 122 | $DCAT=\alpha_{cell}\cdot[CAT]$ |
| 123 | $DCCC=\alpha_{cell}\cdot[CCC]$ |
| 124 | $DCIA=\alpha_{cell}\cdot[CIA]$ |
| 125 | $DCO2=\alpha_{cell}\cdot[CO2]$ |
| 126 | $DCP=\alpha_{cell}\cdot[CP]$ |
| 127 | $DDNA=\alpha_{cell}\cdot[DNA]$ |
| 128 | $DETC=\alpha_{cell}\cdot[ETC]$ |
| 129 | $DF2=\alpha_{cell}\cdot[F2]$ |
| 130 | $DF3=\alpha_{cell}\cdot[F3]$ |
| 131 | $DFC=\alpha_{cell}\cdot[FC]$ |
| 132 | $DFT3=\alpha_{cell}\cdot[FT3]$ |
| 133 | $DFT5=\alpha_{cell}\cdot[FT5]$ |
| 134 | $DFM=\alpha_{cell}\cdot[FM]$ |
| 135 | $DGRX=\alpha_{cell}\cdot[GRX]$ |
| 136 | $DHEM=\alpha_{cell}\cdot[HEM]$ |
| 137 | $DHEME=\alpha_{cell}\cdot[HEME]$ |
| 138 | $DHMX=\alpha_{cell}\cdot[HMX]$ |
| 139 | $DISA=\alpha_{cell}\cdot[ISA]$ |
| 140 | $DISU=\alpha_{cell}\cdot[ISU]$ |
| 141 | $DLEU=\alpha_{cell}\cdot[LEU]$ |
| 142 | $DLYS=\alpha_{cell}\cdot[LYS]$ |
| 143 | $DMEM=\alpha_{cell}\cdot[MEM]$ |
| 144 | $DMP=\alpha_{cell}\cdot[MP]$ |
| 145 | $DMRS=\alpha_{cell}\cdot[MRS]$ |
| 146 | $DNAD=\alpha_{cell}\cdot[NAD]$ |
| 147 | $DNADM=\alpha_{cell}\cdot[NADM]$ |
| 148 | $DNADV=\alpha_{cell}\cdot[NADV]$ |
| 149 | $DNAH=\alpha_{cell}\cdot[NAH]$ |
| 150 | $DNAHM=\alpha_{cell}\cdot[NAHM]$ |
| 151 | $DNAHV=\alpha_{cell}\cdot[NAHV]$ |
| 152 | $DNUC=\alpha_{cell}\cdot[NUC]$ |
| 153 | $DNUCM=\alpha_{cell}\cdot[NUCM]$ |
| 154 | $DNUCMN=\alpha_{cell}\cdot[NUCMN]$ |
| 155 | $DO2=\alpha_{cell}\cdot[O2]$ |
| 156 | $DO2M=\alpha_{cell}\cdot[O2M]$ |
| 157 | $DO2V=\alpha_{cell}\cdot[O2V]$ |
| 158 | $DPL=\alpha_{cell}\cdot[PL]$ |
| 159 | $DPOL=\alpha_{cell}\cdot[POL]$ |
| 160 | $DPRO=\alpha_{cell}\cdot[PRO]$ |
| 161 | $DRIB=\alpha_{cell}\cdot[RIB]$ |
| 162 | $DROS=\alpha_{cell}\cdot[ROS]$ |
| 163 | $DROSE=\alpha_{cell}\cdot[ROSE]$ |
| 164 | $DROSM=\alpha_{cell}\cdot[ROSM]$ |
| 165 | $DROSN=\alpha_{cell}\cdot[ROSN]$ |
| 166 | $DTCA=\alpha_{cell}\cdot[TCA]$ |
| 167 | $DTCAM=\alpha_{cell}\cdot[TCAM]$ |
| 168 | $DTCAMC=\alpha_{cell}\cdot[TCAMC]$ |
| 169 | $DYAP=\alpha_{cell}\cdot[YAP]$ |

**Table S12:** Fractional change in the steady-state concentration of each component due to a 50% decrease in kACMRS.

| Component | **U_ss(10% ↓ kACMRS)_** |
| --- | --- |
| AA | 1.028537 |
| aaCIA | 1.006943 |
| aAFT | 1.170715 |
| aaISA | 1.13285 |
| aaISU | 1.279283 |
| AAM | 1.031924 |
| ACA | 0.993376 |
| ACAC | 0.993387 |
| aCAT | 1.403428 |
| aCIA | 0.953515 |
| ADP | 0.974802 |
| ADPM | 0.974974 |
| aETC | 1.162237 |
| AFT | 1.066383 |
| aFT3 | 1.355261 |
| aFT5 | 1.090446 |
| aGRX | 1.116396 |
| aHEM | 1.015945 |
| aHMX | 1.09128 |
| aISA | 1.095854 |
| aISU | 1.10276 |
| aLEU | 1.278559 |
| aLYS | 1.495539 |
| aMEM | 1.518843 |
| aNUC | 1.006797 |
| aPOL | 1.008978 |
| aRIB | 1.006797 |
| aTCA | 1.334056 |
| ATM | 0.994044 |
| ATP | 0.994149 |
| ATPM | 0.993815 |
| aYAP | 0.99318 |
| CAT | 0.974892 |
| CCC | 0.994136 |
| CIA | 1.030592 |
| CO2 | 1.000229 |
| CP | --- |
| DNA | 0.990498 |
| ETC | 0.988838 |
| F2 | 0.967493 |
| F3 | 0.968956 |
| FC | 0.949404 |
| FT3 | 0.972224 |
| FT5 | 0.881348 |
| FM | 0.881676 |
| GRX | 0.97752 |
| HEM | 0.971137 |
| HEME | 0.730657 |
| HMX | 0.881225 |
| ISA | 0.886413 |
| ISU | 0.874447 |
| LEU | 0.961872 |
| LYS | 0.984866 |
| MEM | 0.991867 |
| MP | --- |
| MRS | 2.031284 |
| NAD | 0.961292 |
| NADM | 0.961074 |
| NADV | 0.995006 |
| NAH | 1.022147 |
| NAHM | 1.022306 |
| NAHV | 1.022133 |
| NUC | 0.992068 |
| NUCM | 1.040294 |
| NUCMN | 1.040138 |
| O2 | 1.000045 |
| O2M | 1.000877 |
| O2V | 1.004642 |
| PL | 1.000506 |
| POL | 0.993138 |
| PRO | 0.993541 |
| RIB | 0.992068 |
| ROS | 1.024362 |
| ROSE | 1.016024 |
| ROSM | 1.019296 |
| ROSN | 1.017742 |
| TCA | 0.987657 |
| TCAM | 1.036683 |
| TCAMC | 1.040425 |
| YAP | 1.000092 |

**Table S13:** Fractional changes in the steady-state concentrations of each component due to a 50% decrease in kMATCA.

| Component | **U_ss(10% ↓ kMATCA)_** |
| --- | --- |
| AA | 0.990957 |
| aaCIA | 1.010679 |
| aAFT | 0.916248 |
| aaISA | 0.852649 |
| aaISU | 0.984059 |
| AAM | 1.011533 |
| ACA | 1.011498 |
| ACAC | 1.000746 |
| aCAT | 1.014162 |
| aCIA | 1.035113 |
| ADP | 1.035001 |
| ADPM | 0.904365 |
| aETC | 1.01808 |
| AFT | 1.028486 |
| aFT3 | 1.008092 |
| aFT5 | 0.893826 |
| aGRX | 1.003123 |
| aHEM | 1.00818 |
| aHMX | 0.841675 |
| aISA | 0.903896 |
| aISU | 1.025126 |
| aLEU | 0.776874 |
| aLYS | 1.032885 |
| aMEM | 1.012622 |
| aNUC | 1.013642 |
| aPOL | 1.012622 |
| aRIB | 4.68695 |
| aTCA | 1.005529 |
| ATM | 1.011055 |
| ATP | 1.011172 |
| ATPM | 1.005821 |
| aYAP | 1.003949 |
| CAT | 1.0223 |
| CCC | 1.002044 |
| CIA | 0.999566 |
| CO2 | --- |
| CP | 1.005091 |
| DNA | 1.005032 |
| ETC | 1.010571 |
| F2 | 1.020725 |
| F3 | 0.996823 |
| FC | 1.004045 |
| FT3 | 0.994131 |
| FT5 | 1.030274 |
| FM | 1.025732 |
| GRX | 1.009655 |
| HEM | 0.982135 |
| HEME | 0.995023 |
| HMX | 1.174218 |
| ISA | 1.114195 |
| ISU | 1.004307 |
| LEU | 1.010355 |
| LYS | 1.005416 |
| MEM | --- |
| MP | 0.971061 |
| MRS | 1.073267 |
| NAD | 1.073697 |
| NADM | 0.995594 |
| NADV | 0.971185 |
| NAH | 0.970919 |
| NAHM | 0.971389 |
| NAHV | 1.005696 |
| NUC | 0.980438 |
| NUCM | 0.980376 |
| NUCMN | 0.99998 |
| O2 | 0.999106 |
| O2M | 0.998531 |
| O2V | 0.999146 |
| PL | 1.0062 |
| POL | 1.006389 |
| PRO | 1.005696 |
| RIB | 0.997258 |
| ROS | 0.998456 |
| ROSE | 0.998369 |
| ROSM | 0.996979 |
| ROSN | 0.942788 |
| TCA | 1.018 |
| TCAM | 1.016821 |
| TCAMC | 1.016679 |
| YAP | 0.990957 |

**Table S14: Proportional change in the steady-state concentration of each component due to a 20% decrease in nutrient IRON.** We again analyzed the decreased ratio of normalized concentrations. The ratio for the apo form of the AFT regulator (aAFT) declined more than 10%. This makes sense as this form activates the iron regulon under Fe-deficient conditions. The ratio for FT3 (the importer on the plasma membrane) declined nearly 7%, shutting down under Fe-replete conditions. The ratio for FC (cytosolic labile Fe pool) increased by nearly 4% and F2 and F3 increased 2-3%. Ratios of virtually all Fe-containing proteins (except CIA) were > 1. All of these changes, except the 0.2% decline in the CIA ratio, make intuitive sense.

| Component | **U_ss(20% ↓ IRON)_** |
| --- | --- |
| AA | 1.004522 |
| aaCIA | 0.999857 |
| aAFT | 1.066288 |
| aaISA | 1.002815 |
| aaISU | 1.005304 |
| AAM | 1.00434 |
| ACA | 0.999621 |
| ACAC | 0.999622 |
| aCAT | 1.000249 |
| aCIA | 0.998042 |
| ADP | 0.998544 |
| ADPM | 0.998548 |
| aETC | 1.003354 |
| AFT | 1.031663 |
| aFT3 | 1.089031 |
| aFT5 | 1.020474 |
| aGRX | 1.003549 |
| aHEM | 0.998836 |
| aHMX | 1.020515 |
| aISA | 1.00139 |
| aISU | 1.002713 |
| aLEU | 0.998355 |
| aLYS | 1.009023 |
| aMEM | 1.019351 |
| aNUC | 1.014942 |
| aPOL | 1.017489 |
| aRIB | 1.014942 |
| aTCA | 1.004871 |
| ATM | 0.999585 |
| ATP | 0.999638 |
| ATPM | 0.999633 |
| aYAP | 0.99954 |
| CAT | 0.999731 |
| CCC | 0.999453 |
| CIA | 1.001006 |
| CO2 | 1.000014 |
| CP | --- |
| DNA | 0.999639 |
| ETC | 0.999936 |
| F2 | 0.985241 |
| F3 | 0.989917 |
| FC | 0.980208 |
| FT3 | 1.04266 |
| FT5 | 1.010174 |
| FM | 0.996037 |
| GRX | 0.999191 |
| HEM | 1.000278 |
| HEME | 1.000023 |
| HMX | 1.010165 |
| ISA | 0.997611 |
| ISU | 0.996203 |
| LEU | 0.999691 |
| LYS | 0.999393 |
| MEM | 0.999504 |
| MP | --- |
| MRS | 1.00943 |
| NAD | 1.002502 |
| NADM | 1.002488 |
| NADV | 0.995612 |
| NAH | 1.001295 |
| NAHM | 1.001304 |
| NAHV | 1.001366 |
| NUC | 0.997848 |
| NUCM | 1.002377 |
| NUCMN | 1.002381 |
| O2 | 1.000001 |
| O2M | 1.000032 |
| O2V | 1.002065 |
| PL | 1.000028 |
| POL | 0.999089 |
| PRO | 0.999557 |
| RIB | 0.997848 |
| ROS | 1.000226 |
| ROSE | 1.000141 |
| ROSM | 1.000164 |
| ROSN | 1.000235 |
| TCA | 0.999465 |
| TCAM | 0.999497 |
| TCAMC | 0.999525 |
| YAP | 0.999867 |

**Table S15: Fractional change in the steady-state concentration of each component due to a 20% decrease in OXYGEN.** Under hypoxic conditions, vacuolar iron is known to become reduced to the FeII state (component F2). The model simulates this, with F2 increasing 54%. Oxygen in all compartments is down about 20%. We had expected ATP levels to decline, due to diminished O2M, but they (ATP an ATPM) increased 6%. ROS levels were unchanged. O2 is involved in the degradation of CAT and ETC, generating FC as iron is released. Under hypoxic conditions, these reactions should be attenuated, causing CAT and ETC to increase and FC to decline. These changes were predicted by this simulation.

| Component | **U_ss(20% ↓ OXYGEN)_** |
| --- | --- |
| AA | 0.899311 |
| aaCIA | 1.008171 |
| aAFT | 1.439391 |
| aaISA | 0.962747 |
| aaISU | 0.824369 |
| AAM | 0.918446 |
| ACA | 1.067553 |
| ACAC | 1.06739 |
| aCAT | 1.397737 |
| aCIA | 0.908804 |
| ADP | 1.223992 |
| ADPM | 1.223038 |
| aETC | 0.904829 |
| AFT | 1.224375 |
| aFT3 | 1.894014 |
| aFT5 | 1.161309 |
| aGRX | 0.840707 |
| aHEM | 1.033429 |
| aHMX | 1.162472 |
| aISA | 0.905015 |
| aISU | 0.956949 |
| aLEU | 1.291907 |
| aLYS | 0.745003 |
| aMEM | 1.952523 |
| aNUC | 1.047814 |
| aPOL | 1.049391 |
| aRIB | 1.047814 |
| aTCA | 0.804558 |
| ATM | 1.033171 |
| ATP | 1.062095 |
| ATPM | 1.063202 |
| aYAP | 1.03282 |
| CAT | 1.003309 |
| CCC | 1.17669 |
| CIA | 1.174636 |
| CO2 | 0.997635 |
| CP | --- |
| DNA | 1.02615 |
| ETC | 1.02303 |
| F2 | 1.522023 |
| F3 | 1.056216 |
| FC | 0.883766 |
| FT3 | 1.080261 |
| FT5 | 0.886905 |
| FM | 1.144731 |
| GRX | 1.077917 |
| HEM | 1.043301 |
| HEME | 0.708608 |
| HMX | 0.891775 |
| ISA | 1.174328 |
| ISU | 1.127176 |
| LEU | 1.010193 |
| LYS | 1.043434 |
| MEM | 1.029359 |
| MP | --- |
| MRS | 1.094689 |
| NAD | 1.047039 |
| NADM | 1.049458 |
| NADV | 1.065078 |
| NAH | 1.077883 |
| NAHM | 1.076184 |
| NAHV | 1.076269 |
| NUC | 1.037315 |
| NUCM | 0.90528 |
| NUCMN | 0.904752 |
| O2 | 0.799855 |
| O2M | 0.795393 |
| O2V | 0.785428 |
| PL | 0.994998 |
| POL | 1.038077 |
| PRO | 1.038365 |
| RIB | 1.037315 |
| ROS | 1.004224 |
| ROSE | 1.004493 |
| ROSM | 1.007447 |
| ROSN | 0.996072 |
| TCA | 1.042405 |
| TCAM | 0.904933 |
| TCAMC | 0.898664 |
| YAP | 1.13891 |

Table S16: Steady-State iron concentrations generated by the model in the ***U_ss(wt)_*** state.

| Specific Component | Compartment | Cellular Concentration (μM) | Local Concentration (μM) | Iron content (from Table S7) | Cellular Iron Concentration (µM) | Local Iron Concentration (µM) |
| --- | --- | --- | --- | --- | --- | --- |
| aaISA | C | 0.03924 | 0.061026 | 3.33922 | 0.131031 | 0.203779 |
| aaISU | C | 0.11772 | 0.183079 | 2.562258 | 0.301629 | 0.469096 |
| aCIA | C | 0.0700362 | 0.108921 | 3.678776 | 0.257647 | 0.400696 |
| AFT | C | 0.03278 | 0.05098 | 1.70668 | 0.055945 | 0.087007 |
| CAT | C | 0.410787 | 0.638860 | 6.905746 | 2.836791 | 4.411805 |
| CIA | C | 0.0700362 | 0.108921 | 22.510144 | 1.576525 | 2.451827 |
| CP | C | 0 | 0 | 1 | 0 | 0 |
| ETC | M | 1.303812 | 13.03812 | 18.652 | 24.3187 | 243.187 |
| F2 | V | 25 | 304.8780 | 1 | 25 | 304.878 |
| F3 | V | 250 | 3048.7805 | 1 | 250 | 3048.781 |
| FC | C | 54 | 83.98134 | 1 | 54 | 83.98134 |
| FT3 | C | 0.046177 | 0.071815 | 2.76376 | 0.127622 | 0.198479 |
| FT5 | V | 0.06505 | 0.79329268 | 1.25888 | 0.08189 | 0.99866 |
| FM | M | 9 | 90 | 1 | 9 | 90 |
| GRX | C | 0.10007 | 0.15563 | 4.93398 | 0.493743 | 0.767875 |
| HEM | C | 0.127297 | 0.1979735 | 7.054408 | 0.898005 | 1.396586 |
| HEME | C | 0.03 | 0.0466563 | 1 | 0.03 | 0.046656 |
| HMX | E | 0.109136 | 4.547325 | 1.00 | 0.109136 | 4.547325 |
| ISA | M | 0.17658 | 1.7658 | 10.487 | 1.851794 | 18.51794 |
| ISU | M | 0.52974 | 5.2974 | 10.153688 | 5.378815 | 53.78815 |
| LEU | C | 0.83123 | 1.292737 | 36.58996 | 30.41467 | 47.3012 |
| LYS | M | 1.62857 | 16.2857 | 9.082034 | 14.79073 | 147.9073 |
| MEM | E | 0.2860731 | 11.9197125 | 23.69787 | 6.779323 | 282.4718 |
| MP | M | 0 | 0 | 1 | 0 | 0 |
| NUC | C | 0.996805 | 1.550241 | 5.537516 | 5.519824 | 8.584484 |
| POL | N | 0.0931609 | 0.6169596 | 34.22987 | 3.188885 | 21.11845 |
| RIB | C | 0.2271681 | 0.353294 | 34.8667 | 7.920602 | 12.3182 |
| TCA | M | 3.712047 | 37.12047 | 29.773 | 110.5188 | 1105.188 |
| YAP | N | 0.01204 | 0.0797351 | 5.00 | 0.0602 | 0.398676 |
|  |  |  |  |  |  |  |
| Whole Cell |  |  |  |  | 556 |  |
| Cytosol |  |  |  |  | 105 | 163 |
| Mitochondria |  |  |  |  | 166 | 1660 |
| Vacuoles |  |  |  |  | 275 | 3350 |
| Endoplasmic Reticulum |  |  |  |  | 6.9 | 84 |
| Nucleus |  |  |  |  | 3.2 | 21 |

**Appendix A:** **Justification for grouping proteins:** The complexity involved in developing a model in which each of the 117 Fe-containing proteins were represented was beyond the scope of this study. Thus, we organized such proteins into 23 groups. We grouped proteins with: a) similar functions; b) the same cellular location; and c) similar iron centers. These criteria could only be approximately applied. At some level, each member protein has a unique function, but once grouped, those uniquenesses are replaced by a common function characterizing the group. In rare situations, members might be located in different cell compartments. Members often had different types of iron centers. Groups were allowed to have a mixture of different types of iron centers, in proportions related to the fraction of the group represented by a given member. Group names were generally the best-studied member, or the best-known function associated with the group. “Guest” members were not included in the list of iron-containing proteins when the study commenced, but during the study, evidence emerged that such proteins might be iron-containing and it was then added to the most appropriate group as a guest.

**Group 1. AFT** includes members Aft1, Aft2, Cth1, and Cth2. All four proteins are involved in iron regulation, especially responding to iron deficiency. Aft1/2 are homologs that bind an [Fe_2_S_2_] cluster bridging a homodimer. Cth1/2 are not iron-containing, but they function in iron regulation, especially extreme iron-deficiency in which they stimulate the degradation of iron-rich proteins thereby releasing needed iron.

**Group 2. ATM** is composed of members Atm1, Mmt1, and Mmt2. All three are mitochondrial iron exporters. Atm1 exports an unknown Fe/S species called “X-S”, and ATM does the same in the model, exporting an [Fe_2_S_2_] cluster into the cytosol. Mmt1/2 are less well studied. None has permanent Fe centers.

**Group 3. CAT** is composed of 6 members, including Aim32, Ccp1, Cta1, Ctt1, Grx6, and Yhb1, as well as “guest” Jlp1. All members of the CAT (catalase) group are involved in combating/degrading ROS in the cells, and many members contain heme groups. This group was assigned to the cytosol, but Cta1 is located in peroxisomes, Grx6 is in the Golgi Apparatus, and Aim32 is in mitochondria.

**Group 4. CCC** consisted of a single protein, Ccc1, which is on the vacuolar membrane. CCC transports cytosolic iron to vacuoles.

**Group 5.** **CIA** is composed of 8 proteins, including Cfd1, Cia1, Cia2, Met18, Dre2, Nar1, Nbp35, and Tah18. All collectively serve to receive an [Fe_2_S_2_] cluster from GRX, convert it to an Fe_4_S_4_ cluster, and then install it in various apo client proteins located in cytosol and nuclei. Cfd1 and Nbp35 form a complex that receives the cluster, Cia1, Cia2 and Met18 form the CIA targeting complex, and Dre2/Tah18 donate electrons as needed for these processes. Nar1 passes the cluster from the Cfd1/Nbp35 complex to the targeting complex. Many of these proteins contain Fe/S clusters.

**Group 6. ETC** is composed of 13 proteins including Cob1, Coq6, Coq7, Cox1, Cox10, Cox15, Cyb2, Cyc1, Cyc2, Cyc7, Cyt1, Mss51, Rip1, and Cir2 (guest). These proteins are all involved in the mitochondrial Electron Transport Chain which transfers electrons from NADH to O2 (indirectly) while pumping protons across the mitochondrial inner membrane. All are located in mitochondria and most contain heme centers.

**Group 7. FT3** consists of 10 proteins, including Fet3, Fet4, Fre1, Fre2, Fre3, Fre4, Fre5, Fre7, Ftr1, and Smf1. Ftr1, Fet4, and Smf1 are iron importers on the plasma membrane. The Fre proteins are heme-containing ferric reductases, and Fet3, the best-known member of the group, is a multi-copper oxidase. Expression of this group is regulated by AFT.

**Group 8.** **FT5** includes the 4 proteins Fet5, Fre6, Fth1, and Smf3. Collectively this group transports vacuolar iron into the cytosol. FT5 is analogous to FT3 but is located in vacuoles rather than the plasma (cytosolic) membrane. Fet5 is a multi-copper oxidase, Fre6 is a heme-containing ferric reductase, and Fth1 and Smf3 are the Fe transporters or permeases.

**Group 9. GRX** is composed of members Bol2, Grx3, Grx4, and Adp1. Homologous monothiol glutaredoxins Grx3/4 are cytosolic proteins involved in transferring [Fe_2_S_2_] clusters. Bol2 is an adapter protein that can form a heterodimer with GRX proteins. Adp1 appears to be involved in cytosolic cluster transfer. The GRX group helps transfer [Fe_2_S_2_] clusters in the cytosol.

**Group 10. HEM** is composed of member proteins Hem15, Hap1/2/3/4/5, Rox1, and Tdh3. Hem15 is mitochondrial ferrochelatase, which builds heme centers. The Hap proteins and Rox1 regulate heme biosynthesis. Tdh3 encodes glyceraldehyde-3-phosphate dehydrogenase, a glycolytic enzyme which has been suggested to be involved in heme trafficking as a moonlighting function. The HEM group catalyzed heme formation and regulated heme trafficking and metabolism.

**Group 11.** **HMX** consists of a single protein Hmx1, heme oxygenase located in the ER and involved in heme degradation.

**Group 12.** **ISA** is composed of 6 member proteins, including Bol1, Bol3, Iba57, Isa1, Isa2, Nfu1, and guest RSM22. Isa1, Isa2, and Iba57 form a complex in mitochondria that convert [Fe_2_S_2_] clusters into [Fe_4_S_4_] clusters. The exact functions of Bol1/3 and Nfu1 are uncertain, but they appear to help in [Fe_4_S_4_] cluster transfer to various mitochondrial apo client proteins. Guest RSM22 is a mitochondrial ribosomal protein that is involved in mitochondrial protein biosynthesis (not an excellent fit for this group).

**Group 13. ISU** is composed of 12 proteins, including Acp1, Arh1, Grx5, Isd11, Isu1, Isu2, Jac1, Mge1, Nfs1, Ssq1, Yah1, and Yfh1. Isu1/2 are scaffold proteins upon which [Fe_2_S_2_] clusters in mitochondria are assembled. Nfs1 is cysteine desulfurase which catalyzes the removal of sulfurs from cysteines, which ultimately become the sulfide ions in the clusters. Acp1, Isd11, and Yfh1 are adaptor proteins that bind to the cluster assembly complex. Yah1 and Arh1 provide electrons (from NAD(P)H) required for this process. Once assembled, the cluster is donated to Grx5, and then to various apo client proteins. Heat-shock proteins Jac1 and Ssq1, and Mge1 are needed for cluster transfer including ATP activation. Some of these proteins bind Fe/S clusters.

**Group 14.** **LEU** is composed of Glt1, Leu1, Met5, Met8, and guest Bna2. These cytosolic proteins are involved in ammo acid biosynthesis (Glt1 for glutamate; Leu1 for leucine; Met5/8 for methionine). Guest Bna2 is involved in the synthesis of NAD (again, not an excellent fit). The LEU group catalyzes the synthesis of amino acid AA in the cytosol.

**Group 15. LYS** is composed of Ilv2, Lys4, Thi4, and Thi5, along with guests Thi13, Fmp12, Thi11, and Thi12. These mitochondrial proteins are involved in amino acid or cofactor biosynthesis (Ilv2 for isoleucine and valine; Lys4 for lysine; Thi proteins for thiamine biosynthesis). In the model, LYS catalyzed the synthesis of AA in mitochondria.

**Group 16.** **MEM** is composed of 14 members including Cyb5, Cyp51, Dap1, Erg3, Erg5, Erg25, Fre8, Mpo1, Ncp1, Ole1, Scs7, Sfh5, Sur2, and Yno1, and guest Dit2. Member proteins are located in the endoplasmic reticulum. These proteins generally contain heme centers and/or Fe-O-Fe iron-oxo dimers. Erg3/5/25, Cyp51, and Ncp1 are involved in ergosterol biosynthesis. Cyb5 is involved in sterol/lipid biosynthesis. ER protein Dap1 might be involved in heme trafficking. Fre8 is a ferric reductase in the ER, likely involved in iron transfer into the ER. Yno1 is similar. Mpo1, Ole1, and Scs7 are involved in fatty acid metabolism. Sfh1 is a heme protein involved in phospholipid transfer. Sur2 is involved in sphingolipid biosynthesis. Dit2, an ER-localized cytochrome P450-like enzyme involved in tyrosine metabolism (not an excellent fit). The MEM group catalyzes the synthesis of phospholipid PL in endoplasmic reticula.

**Group 17.** **MRS** is composed of mitochondrial proteins Mrs3, Mrs4, and Rim2. These three proteins are located on the mitochondrial inner membrane and function to catalyze the import of cytosolic iron into mitochondria.

**Group 18.** **NUC** is composed of cytosolic member proteins Bna1, Rnr2, Rnr4, and Tyw1. Bna1 is involved in NAD biosynthesis, Rnr2/4 are subunits of ribonucleotide reductase, and Tyw1 helps modify tRNA. Group NUC catalyzes the synthesis of the only nucleotide in the cell, called NUCM, which is used in the synthesis of DNA.

**Group 19.** **POL** is composed of 11 proteins, including nuclear proteins Chl1, Dna2, Ntg2, Pol1, Pol2, Pol3, Pri2, Rad3, Rev3, Tpa1, and Exo5, and guests Jhd1 and Jhd2. Chl1, Dna2, Ntg2, Pol1/2/3 and Pri2, Rad3, and Rev3 are all used in DNA synthesis and/or repair. They all contain [Fe_4_S_4_] clusters. Group POL catalyzes the synthesis of DNA in the nucleus. Tpa1 is involved in mitochondrial translation, and Exo5 is used in mitochondrial genome maintenance. Guests Jhd1/2 are histone demethylases with uncertain locations (not an excellent fit).

**Group 20. PRO** includes 5716 yeast proteins that are not otherwise involved in the model. Apart from consuming amino acids and ATP, this protein group had no influence on any reaction.

**Group 21.** **RIB** is composed of member proteins Cbr1, Dbr1, Dph1, Dph2, Dph3, Dph4, Elp3, Lto1, Rli1, Yae1, Lia1, and Ncs6, and guest Ate1. Cbr1, Elp3, and Ncs6 are used to modify tRNAs, Dbr1 is used in mRNA splicing, Dph1/2/3/4 modify translation elongation factor 2, Lto1 and Yae1 form a complex that helps recruit apo-Rli1 to the CIA targeting complex and is required for ribosome biosynthesis and translation initiation. Ate1 is involved in translation and tRNA modification. This group, symbolizing ribosome function, along with DNA, catalyzes the synthesis of all protein groups.

**Group 22. TCA** includes proteins Aco1, Aco2, Bio2, Lip5, Sdh2, Sdh3, and Sdh4, and guests Shh3 and Shh4. This group represents the TriCarboxylic Acid cycle in mitochondria, which is involved in respiration. Aco1/2 are two isoforms of aconitase, Sdh2/3/4 and Shh3 and Shh4 are succinate dehydrogenase subunits or derivatives thereof. Lip5 catalyzes the synthesis of lipoic acid which is required by some TCA enzymes. Bio2 catalyzes the synthesis of biotin which is used in an anaplerotic reaction needed for the TCA cycle.

**Group 23.** **YAP** consists of a single member protein, Yap5, and guest Yap7, both located in the nucleus. YAP catalyzes the synthesis of CCC and aGRX under high-iron conditions. Yap7 is a paralog of Yap5 that regulated RNA polymerase.

**Appendix B: Independent reaction-rate selections and compatibility relationships:** The 89 independent rates *R_j_* (called R[j] below where *j* refers to the number for the reactions listed in Table S8) were chosen to be:

R[2], R[6], R[18], R[19], R[45], R[47], R[48], R[70], R[75], R[83], R[86], R[88], R[89], R[90], R[91], R[92], R[93], R[94], R[95], R[96], R[97], R[98], R[99], R[100], R[101], R[102], R[103], R[104], R[105], R[106], R[107], R[108], R[109], R[110], R[111], R[112], R[113], R[114], R[115], R[116], R[117], R[118], R[119], R[121], R[122], R[123], R[124], R[125], R[127], R[128], R[129], R[130], R[131], R[132], R[133], R[134], R[135], R[136], R[137], R[138], R[139], R[140], R[141], R[142], R[143], R[144], R[145], R[147], R[148], R[149], R[150], R[151], R[152], R[153], R[154], R[155], R[156], R[157], R[158], R[159], R[160], R[161], R[162], R[163], R[165], R[166], R[167], R[168], and R[169].

The remaining 80 dependent rates were solved from the following compatibility relationships:

R[1] = 0.0618224 R[47] - 5.93837*10^-9 R[83] - 1.94982*10^-6 R[86] + 0.484596 R[99] + 0.224815 R[103] + 0.0618224 R[122] + 2.96521 R[124] + 0.649942 R[135] + 5.19175 R[141] + 0.398469 R[152] + 4.08 R[159] + 4.09452 R[161] + 0.658634 R[169],

R[3] = R[6] + R[129] + R[130] + 4.44089*10^-16 R[148],

R[4] = - 18. R[18] - 1.81899*10^-12 R[19] - 77.7608 R[47] - 158.889 R[48] + 2.77556*10^-17 R[70] - 0.5 R[75] + 2.22207*10^-6 R[83] + 0.0014592 R[86] - 13.0843 R[88] - 0.00997345 R[8]- 0.25 R[90] - 1166.98 R[91] - 549.449 R[92] - 315.672 R[93] - 645.675 R[94] - 0.25 R[95] - 417.465 R[98] - 1166.97 R[99] + 5.55112*10^-17 R[101] - 1070.52 R[102] - 549.449 R[103] - 1521.45 R[104] - 553.925 R[105] - 243.2 R[106] - 776.85 R[107] - 79.25 R[108] - 315.675 R[109] - 645.936 R[110] - 1749.78 R[111] - 587.1 R[112] - 1305.65 R[113] - 473.2 R[114] - 2913.67 R[115] - 1203.93 R[116] - 1147.2 R[117] - 445.55 R[118] - 61.25 R[121] - 424.437 R[122] - 80.5 R[123] - 1169.33 R[124] + 0.3 R[125] - 3874.99 R[127] - 1084.77 R[128] - 1524.44 R[132] - 555.289 R[133] - 243.199 R[135] - 784.492 R[136] - 1.08333 R[137] - 80.3333 R[138] - 316.569 R[139] - 645.936 R[140] - 1754.82 R[141] - 587.433 R[142] - 1314.58 R[143] - 251.15 R[145] + 5.55112*10^-17 R[147] - 0.5 R[148] - 0.5 R[149] - 0.5 R[150] - 0.5 R[151] - 473.575 R[152] - 0.5 R[153] - 0.499997 R[154] - 18. R[158] - 2917.55 R[159] - 757370. R[160] - 1207.81 R[161] - 1151.15 R[166] - 61.5007 R[169],

R[5] = 3. R[2] + 3. R[18] + 3. R[19] - 0.0000256723 R[47] - 0.0000547616 R[48] - 3. R[83] - 0.000014802 R[86] - 9.70021*10^-9 R[88] + 0.0000290626 R[89] + 3.67878 R[99] + 1.70666 R[103] + 3.33922 R[109] + 2.56226 R[110] + 6.90572 R[122] + 22.5102 R[124] + 18.6519 R[128] + R[129] + R[130] + R[131] + 2.76376 R[132] + 1.25888 R[133] + R[134] + 4.93399 R[135] + 7.05441 R[136] + R[137] + R[138] + 10.4874 R[139] + 10.1537 R[140] + 36.5902 R[141] + 9.08203 R[142] + 23.698 R[143] + R[144] + 5.53752 R[152] + 34.2297 R[159] + 34.8677 R[161] + 3. R[162] + 3.00001 R[163] + 2.99998 R[165] + 29.7735 R[166] + 4.99998 R[169],

R[7] = - 1.81899*10^-12 R[19] - 0.0223291 R[47] + 0.0000120816 R[48] - 0.5 R[70] - 4.44413*10^-6 R[83] - 0.0029184 R[86] + 0.0000611484 R[88] + 0.0198818 R[89] + 0.5 R[90] + 2333.95 R[91] + 1098.9 R[92] + 631.35 R[93] + 1291.35 R[94] + 834.93 R[98] + 2333.95 R[99] + 2141.05 R[102] + 1098.9 R[103] + 3042.9 R[104] + 1107.85 R[105] + 486.4 R[106] + 1553.7 R[107] + 158.5 R[108] + 631.35 R[109] + 1291.35 R[110] + 3499.55 R[111] + 1174.2 R[112] + 2611.3 R[113] + 946.4 R[114] + 5827.35 R[115] + 2407.85 R[116] + 2294.4 R[117] + 891.1 R[118] + 122.5 R[121] + 834.928 R[122] + 161. R[123] + 2333.95 R[124] + 7749.96 R[127] + 2141.05 R[128] + 3042.9 R[132] + 1107.85 R[133] + 486.4 R[135] + 1553.7 R[136] - 9.99632*10^-7 R[137] + 158.5 R[138] + 631.35 R[139] + 1291.35 R[140] + 3499.56 R[141] + 1174.2 R[142] + 2611.3 R[143] + 502.3 R[145] + 946.4 R[152] + R[153] + R[154] + 5827.32 R[159] + 1.51474*10^6 R[160] + 2407.86 R[161] + 2294.4 R[166] + 122.499 R[169],

R[8] = 7.50914 R[47] + 15.3409 R[48] + 0.5 R[70] + 1.16667 R[88] + 0.0000350731 R[89] + 0.5 R[95] + 7.50914 R[122] + 15.3409 R[128] + 3.22439 R[132] + 1.46869 R[133] + 8.23014 R[136] + 1.16667 R[137] + 1.16667 R[138] + 0.121287 R[141] + 9.59337 R[143] + 0.394234 R[166],

R[9] = R[18] + R[158],

R[10] = 6.90572 R[47] + 18.6519 R[48] - 4.50807*10^-8 R[83] - 0.000014802 R[86] + R[88] + 0.0000300626 R[89] + 3.67878 R[99] + 1.70666 R[103] + 3.33922 R[109] + 2.56226 R[110] + 6.90572 R[122] + 22.5102 R[124] + 18.6519 R[128] + 2.76376 R[132] + 1.25888 R[133] + R[134] + 4.93399 R[135] + 7.05441 R[136] + R[137] + R[138] + 10.4874 R[139] + 10.1537 R[140] + 39.5167 R[141] + 9.08203 R[142] + 8.22288 R[143] + R[144] + 3.02495 R[152] + 30.973 R[159] + 31.0833 R[161] + 29.7735 R[166] + 4.99998 R[169],

R[11] = 7750. R[19] + 7749.96 R[127] + R[153] + 0.999995 R[154],

R[12] = R[19] + R[127],

R[13] = - 45.054 R[47] - 92.046 R[48] - 7.00062 R[88] - 0.000995464 R[91] + 0.000491027 R[92] + 0.00182184 R[93] + 0.0000365234 R[98] + 0.00158095 R[99] - 0.00025017 R[107] + 9.53681*10^-6 R[121] + 0.2 R[125] - 0.00582392 R[127] + 0.000986939 R[135] + 4.86046*10^-6 R[137] - 0.0118982 R[143] + 0.00184769 R[152] - 0.0112596 R[159] - 0.000762589 R[169],

R[14] = 0.5 R[75] + 0.25 R[130] + 0.5 R[148],

R[15] = R[75] + R[148],

R[16] = R[2] + R[18] + R[19] - R[83] + R[162] + R[163] + R[165],

R[17] = 0.333333 R[144],

R[20] = 22.5051 R[47] + 46.0229 R[48] - 4.44413*10^-6 R[83] - 0.0029184 R[86] + 3.50006 R[88] + 0.019987 R[89] + 0.5 R[90] + 2333.95 R[91] + 1098.9 R[92] + 631.35 R[93] + 1291.35 R[94] + 0.5 R[95] + 834.93 R[98] + 2333.95 R[99] + 2141.05 R[102] + 1098.9 R[103] + 3042.9 R[104] + 1107.85 R[105] + 486.4 R[106] + 1553.7 R[107] + 158.5 R[108] + 631.35 R[109] + 1291.35 R[110] + 3499.55 R[111] + 1174.2 R[112] + 2611.3 R[113] + 946.4 R[114] + 5827.35 R[115] + 2407.85 R[116] + 2294.4 R[117] + 891.1 R[118] + 122.5 R[121] + 857.455 R[122] + 161. R[123] + 2333.95 R[124] + 7749.96 R[127] + 2187.07 R[128] + 3052.57 R[132] + 1112.26 R[133] + 486.4 R[135] + 1578.39 R[136] + 3.5 R[137] + 162. R[138] + 631.35 R[139] + 1291.35 R[140] + 3499.93 R[141] + 1174.2 R[142] + 2640.08 R[143] + 502.3 R[145] + 946.4 R[152] + R[153] + R[154] + 5827.32 R[159] + 1.51474*10^6 R[160] + 2407.86 R[161] + 2295.58 R[166] + R[167] + R[168] + 122.499 R[169],

R[21] = R[91] + R[99] + R[124] + 5.*10^-6 R[141] - 7.72498*10^-7 R[159] + 2.*10^-6 R[161] - 4.98818*10^-8 R[169],

R[22] = R[92] + R[103],

R[23] = R[93] + R[109] + R[139] + 4.66563*10^-9 R[166],

R[24] = R[94] + R[110] + R[140] + 6.22084*10^-10 R[166],

R[25] = - 500. R[18] - 193750. R[19] + 5. R[45] - 26867. R[47] - 646030. R[48] - 10. R[75] + 0.00015559 R[83] + 0.102159 R[86] - 302.527 R[88] - 0.695637 R[89] - 2.5 R[90] - 81688.3 R[91] - 38461.5 R[92] - 22095.7 R[93] - 45197.3 R[94] - 2.5 R[95] + 5. R[96] + 5. R[97] - 29223.1 R[98] - 81691.9 R[99] + 5. R[100] + 5. R[101] - 653020. R[102] - 38463.2 R[103] - 106502. R[104] - 38774.8 R[105] - 17024. R[106] - 54379.5 R[107] - 5547.5 R[108] - 22095.8 R[109] - 45202.5 R[110] - 122484. R[111] - 41097. R[112] - 91395.5 R[113] - 33124. R[114] - 203957. R[115] - 84274.8 R[116] - 80304. R[117] - 31188.5 R[118] - 4287.5 R[121] - 29175.3 R[122] - 5635. R[123] - 81758.2 R[124] + 8. R[125] - 232499. R[127] - 652922. R[128] - 106481. R[132] - 38765.3 R[133] - 17029. R[135] - 54326.6 R[136] + 7.50023 R[137] - 5540. R[138] - 22113.6 R[139] - 45202.5 R[140] - 122622. R[141] - 41103.7 R[142] - 91334.4 R[143] - 17580.5 R[145] - 10. R[148] - 10. R[149] - 10. R[150] - 10. R[151] - 33134.6 R[152] - 30. R[153] - 29.9998 R[154] - 450.001 R[158] - 204065. R[159] - 5.30159*10^7 R[160] - 84384.1 R[161] - 80373.2 R[166] + 10. R[167] + 10. R[168] - 4297.56 R[169],

R[26] = R[47] + R[98] + R[122],

R[27] = - 50. R[18] - 19375. R[19] - 0.5 R[45] - 2686.68 R[47] - 64603. R[48] - 1. R[75] + 0.0000200032 R[83] + 0.0131343 R[86] - 30.2525 R[88] - 0.0895506 R[89] - 0.75 R[90] - 10502.8 R[91] - 4945.05 R[92] - 2840.92 R[93] - 5811.07 R[94] - 0.75 R[95] - 3757.24 R[98] - 10503.1 R[99] + 0.5 R[100] + 0.5 R[101] - 67443.1 R[102] - 4945.22 R[103] - 13693. R[104] - 4985.32 R[105] - 2188.8 R[106] - 6991.65 R[107] - 713.25 R[108] - 2840.93 R[109] - 5811.6 R[110] - 15748. R[111] - 5283.9 R[112] - 11750.8 R[113] - 4258.8 R[114] - 26223.1 R[115] - 10835.3 R[116] - 10324.8 R[117] - 4009.95 R[118] - 551.25 R[121] - 3774.98 R[122] - 724.5 R[123] - 10509.8 R[124] + 0.7 R[125] - 30999.9 R[127] - 67479.2 R[128] - 13700.7 R[132] - 4988.79 R[133] - 2189.3 R[135] - 7011.05 R[136] - 2.74998 R[137] - 716. R[138] - 2842.71 R[139] - 5811.6 R[140] - 15762.2 R[141] - 5284.57 R[142] - 11773.5 R[143] - 2260.35 R[145] - R[148] - R[149] - R[150] - R[151] - 4259.86 R[152] - 4. R[153] - 3.99998 R[154] - 50.0001 R[158] - 26233.8 R[159] - 6.81633*10^6 R[160] - 10846.3 R[161] - 10332.9 R[166] - 552.255 R[169],

R[28] = R[48] + R[102] + R[128],

R[29] = R[104] + R[132],

R[30] = R[105] + R[133],

R[31] = - 5.*10^-6 R[47] - 9.13678*10^-9 R[83] - 6.*10^-6 R[86] - 3.*10^-6 R[103] + R[106] - 5.*10^-6 R[122] + 10^-6 R[124] + R[135] + 2.09293*10^-6 R[141] + 1.60633*10^-7 R[152] - 1.87959*10^-6 R[159] + 1.65061*10^-6 R[161] - 4.06942*10^-7 R[169],

R[32] = 6.62349*10^-8 R[47] + 7.77605*10^-9 R[48] + 3.93566*10^-8 R[88] + 8.52308*10^-6 R[89] + R[107] + 6.62349*10^-8 R[122] + 7.77605*10^-9 R[128] + 3.67002*10^-8 R[132] + R[136] - 6.43388*10^-10 R[137] - 1.49205*10^-8 R[141] - 1.7403*10^-6 R[143] + 5.44324*10^-11 R[166],

R[33] = 0.0000418894 R[89] + R[108] + R[138],

R[34] = R[111] + R[141],

R[35] = R[112] + R[142],

R[36] = R[113] + R[143],

R[37] = R[114] + R[152],

R[38] = R[115] + R[159],

R[39] = R[116] + R[161],

R[40] = R[117] + R[166],

R[41] = R[118],

R[42] = R[121] + R[169],

R[43] = R[123],

R[44] = R[145],

R[46] = R[160],

R[49] = R[99] + R[124] + 5.*10^-6 R[141] - 7.72498*10^-7 R[159] + 2.*10^-6 R[161] - 4.98818*10^-8 R[169],

R[50] = R[86] + R[103],

R[51] = R[109] + R[139] + 4.66563*10^-9 R[166],

R[52] = R[110] + R[140] + 6.22084*10^-10 R[166],

R[53] = R[47] + R[122],

R[54] = R[124] + 2.09293 R[141] + 0.160633 R[152] + 1.64476 R[159] + 1.65061 R[161] + 0.106205 R[169],

R[55] = R[48] + R[128],

R[56] = R[132],

R[57] = R[133],

R[58] = 0.912394 R[47] + 1.864 R[48] + 0.141755 R[88] + 4.26154*10^-6 R[89] + 0.912394 R[122] + 1.864 R[128] + 0.391778 R[132] + 0.178453 R[133] + 1. R[136] + 0.141755 R[137] + 0.141755 R[138] + 0.0147369 R[141] + 1.16564 R[143] + 0.0479013 R[166],

R[59] = R[88] - 8.22283 R[89] + R[138],

R[60] = 0.29192 R[110] + R[139] + 0.29192 R[140] + 0.372519 R[142] + 4.01492 R[166],

R[61] = 0.0618224 R[47] + 0.667763 R[48] - 5.93837*10^-9 R[83] - 1.94982*10^-6 R[86] + 0.484596 R[99] + 0.224815 R[103] + 0.439867 R[109] + 0.274875 R[110] + 0.0618224 R[122] + 2.96521 R[124] + 0.667763 R[128] + 0.649942 R[135] + 1.38148 R[139] + 1.27487 R[140] + 5.19175 R[141] + 1.16351 R[142] + 0.398469 R[152] + 4.08 R[159] + 4.09452 R[161] + 3.97448 R[166] + 0.658634 R[169],

R[62] = R[141],

R[63] = R[142],

R[64] = R[89] + R[143],

R[65] = R[152],

R[66] = R[159],

R[67] = R[161],

R[68] = R[166],

R[69] = R[169],

R[71] = - 0.0438474 R[47] - 0.000284765 R[48] - 8.88826*10^-6 R[83] - 0.0058368 R[86] - 0.000498044 R[88] + 0.039974 R[89] + R[90] + 4667.9 R[91] + 2197.8 R[92] + 1262.7 R[93] + 2582.7 R[94] + R[95] + R[96] + 1669.86 R[98] + 4667.9 R[99] + 4282.1 R[102] + 2197.8 R[103] + 6085.8 R[104] + 2215.7 R[105] + 972.8 R[106] + 3107.4 R[107] + 317. R[108] + 1262.7 R[109] + 2582.7 R[110] + 6999.1 R[111] + 2348.4 R[112] + 5222.6 R[113] + 1892.8 R[114] + 11654.7 R[115] + 4815.7 R[116] + 4588.8 R[117] + 1782.2 R[118] + 245. R[121] + 1714.91 R[122] + 322. R[123] + 4667.9 R[124] + 0.2 R[125] + 15499.9 R[127] + 4374.15 R[128] + 6105.15 R[132] + 2224.51 R[133] + 972.802 R[135] + 3156.78 R[136] + 7. R[137] + 324. R[138] + 1262.7 R[139] + 2582.7 R[140] + 6999.85 R[141] + 2348.4 R[142] + 5280.15 R[143] + 1004.6 R[145] + 1892.8 R[152] + 2. R[153] + 2 R[154] + 11654.6 R[159] + 3.02948*10^6 R[160] + 4815.71 R[161] + 4591.17 R[166] + 2. R[167] + 2. R[168] + 244.997 R[169],

R[72] =- 72. R[18] - 1.45519*10^-11 R[19] - 363.701 R[47] - 742.942 R[48] - 0.5 R[70] - 2. R[75] + 8.8974*10^-6 R[83] + 0.0058398 R[86] - 60.5045 R[88] - 0.0399289 R[89] - R[90] - 4667.91 R[91] - 2197.8 R[92] - 1262.69 R[93] - 2582.7 R[94] - 1.5 R[95] - 1669.86 R[98] - 4668.63 R[99] + R[101] - 4282.1 R[102] - 2198.14 R[103] - 6085.8 R[104] - 2215.7 R[105] - 972.8 R[106] - 3107.4 R[107] - 317. R[108] - 1262.7 R[109] - 2583.74 R[110] - 6999.1 R[111] - 2348.4 R[112] - 5222.6 R[113] – 1892.8 R[114] – 11654.7 R[115] – 4815.7 R[116] – 4588.8 R[117] - 1782.2 R[118] - 245. R[121] - 1705.35 R[122] - 322. R[123] - 4681.88 R[124] + 1.4 R[125] - 15500. R[127] - 4354.42 R[128] - 6101. R[132] - 2222.62 R[133] - 973.794 R[135] - 3146.2 R[136] - 5.49996 R[137] - 322.5 R[138] - 1266.27 R[139] - 2583.74 R[140] - 7027.39 R[141] - 2349.73 R[142] - 5267.92 R[143] - 1004.6 R[145] - 2. R[148] - 2. R[149] - 2. R[150] - 2. R[151] - 1894.91 R[152] - 2. R[153] - 2 R[154] - 72.0002 R[158] - 11676.5 R[159] - 3.02948*10^6 R[160] - 4837.55 R[161] - 4605.01 R[166] - 247.017 R[169],

R[73] = - 22. R[18] + 19375. R[19] + 0.5 R[45] + 2322.98 R[47] + 63860. R[48] - 0.5 R[70] - R[75] - 0.0000111058 R[83] - 0.0072945 R[86] - 30.252 R[88] + 0.0496218 R[89] - 0.25 R[90] + 5834.87 R[91] + 2747.25 R[92] + 1578.23 R[93] + 3228.38 R[94] - 0.75 R[95] + 2087.39 R[98] + 5834.51 R[99] + 0.5 R[100] + 0.5 R[101] + 63161. R[102] + 2747.07 R[103] + 7607.25 R[104] + 2769.63 R[105] + 1216. R[106] + 3884.25 R[107] + 396.25 R[108] + 1578.23 R[109] + 3227.85 R[110] + 8748.87 R[111] + 2935.5 R[112] + 6528.25 R[113] + 2366. R[114] + 14568.4 R[115] + 6019.63 R[116] + 5736. R[117] + 2227.75 R[118] + R[119] + 306.25 R[121] + 2069.63 R[122] + 402.5 R[123] + 5827.89 R[124] + 0.7 R[125] + 15499.9 R[127] + 63124.8 R[128] + 7599.65 R[132] + 2766.16 R[133] + 1215.5 R[135] + 3864.85 R[136] - 2.74999 R[137] + 393.5 R[138] + 1576.44 R[139] + 3227.85 R[140] + 8734.77 R[141] + 2934.83 R[142] + 6505.6 R[143] + 1255.75 R[145] + 2.22045*10^-16 R[147] - R[148] - R[149] - R[150] - R[151] + 2364.94 R[152] + 2. R[153] + 2 R[154] - 22. R[158] + 14557.4 R[159] + 3.78685*10^6 R[160] + 6008.72 R[161] + 5727.9 R[166] + 305.238 R[169],

R[74] = 36. R[18] - 1.81899*10^-12 R[19] + 6.41407 R[47] + 13.1494 R[48] - 0.5 R[70] + R[75] - 4.44413*10^-6 R[83] - 0.0029184 R[86] + 3.00006 R[88] + 0.0198818 R[89] + 0.5 R[90] + 2333.95 R[91] + 1098.9 R[92] + 631.35 R[93] + 1291.35 R[94] + 834.93 R[98] + 2333.95 R[99] + 2141.05 R[102] + 1098.9 R[103] + 3042.9 R[104] + 1107.85 R[105] + 486.4 R[106] + 1553.7 R[107] +158.5 R[108] + 631.35 R[109] + 1291.35 R[110] + 3499.55 R[111] + 1174.2 R[112] + 2611.3 R[113] + 946.4 R[114] + 5827.35 R[115] + 2407.85 R[116] + 2294.4 R[117] + 891.1 R[118] + 122.5 R[121] + 834.928 R[122] + 161. R[123] + 2338.66 R[124] + 7749.96 R[127] + 2141.05 R[128] + 3042.9 R[132] + 1107.85 R[133] + 486.4 R[135] + 1553.7 R[136] - 1.00845*10^-6 R[137] + 158.5 R[138] + 631.35 R[139] + 1291.35 R[140] + 3509.42 R[141] + 1174.2 R[142] + 2611.3 R[143] + 502.3 R[145] + R[147] + R[148] + R[149] + R[150] + R[151] + 947.156 R[152] + R[153] + R[154] + 36.0001 R[158] + 5835.06 R[159] + 1.51474*10^6 R[160] + 2415.63 R[161] + 2294.4 R[166] + 122.999 R[169],

R[76] = R[75] + R[148] + R[151],

R[77] = 36. R[18] - 1.81899*10^-12 R[19] + 6.41407 R[47] + 13.1494 R[48] - 0.5 R[70] + 1. R[75] - 4.44413*10^-6 R[83] - 0.0029184 R[86] + 3.00006 R[88] + 0.0198818 R[89] + 0.5 R[90] + 2333.95 R[91] + 1098.9 R[92] + 631.35 R[93] + 1291.35 R[94] + 834.93 R[98] + 2333.95 R[99] + 2141.05 R[102] + 1098.9 R[103] + 3042.9 R[104] + 1107.85 R[105] + 486.4 R[106] + 1553.7 R[107] + 158.5 R[108] + 631.35 R[109] + 1291.35 R[110] + 3499.55 R[111] + 1174.2 R[112] + 2611.3 R[113] + 946.4 R[114] + 5827.35 R[115] + 2407.85 R[116] + 2294.4 R[117] + 891.1 R[118] + 122.5 R[121] + 834.928 R[122] + 161. R[123] + 2338.66 R[124] + 7749.96 R[127] + 2141.05 R[128] + 3042.9 R[132] + 1107.85 R[133] + 486.4 R[135] + 1553.7 R[136] - 9.99632*10^-7 R[137] + 158.5 R[138] + 631.35 R[139] + 1291.35 R[140] + 3509.42 R[141] + 1174.2 R[142] + 2611.3 R[143] + 502.3 R[145] + R[148] + R[149] + R[151] + 947.156 R[152] + R[153] + R[154] + 36.0001 R[158] + 5835.06 R[159] + 1.51474*10^6 R[160] + 2415.63 R[161] + 2294.4 R[166] + 122.999 R[169],

R[78] = 7750. R[19] + 7749.96 R[127] + R[154],

R[79] = R[2] - 13. R[18] + R[19] - 71.3244 R[47] - 145.739 R[48] - 0.999998 R[83] + 0.0014592 R[86] - 10.0843 R[88] - 0.00997345 R[89] - 0.25 R[90] - 1166.98 R[91] - 549.449 R[92] - 315.672 R[93] - 645.675 R[94] - 0.25 R[95] - 417.465 R[98] - 1166.97 R[99] - 1070.52 R[102] - 549.449 R[103] - 1521.45 R[104] - 553.925 R[105] - 243.2 R[106] - 776.85 R[107] - 79.25 R[108] - 315.675 R[109] - 645.936 R[110] - 1749.77 R[111] - 587.1 R[112] - 1305.65 R[113] - 473.2 R[114] - 2913.67 R[115] - 1203.93 R[116] - 1147.2 R[117] - 445.55 R[118] - 61.25 R[121] - 424.437 R[122] - 80.5 R[123] - 1169.33 R[124] + 0.3 R[125] - 3874.99 R[127] - 1084.77 R[128] + 0.25 R[130] - 1524.44 R[132] - 555.289 R[133] - 243.199 R[135] - 784.492 R[136] - 1.08333 R[137] - 80.3333 R[138] - 316.569 R[139] - 645.936 R[140] - 1754.82 R[141] - 587.433 R[142] - 1314.58 R[143] + 0.333333 R[144] - 251.15 R[145] - 0.5 R[149] - 0.5 R[150] - 0.5 R[151] - 473.575 R[152] - 0.5 R[157] - 14. R[158] - 2917.55 R[159] - 757370. R[160] - 1207.81 R[161] + 1. R[162] + R[163] + R[165] - 1151.15 R[166] - 61.5007 R[169],

R[80] = - 18. R[18] - 77.7608 R[47] - 158.889 R[48] - 0.5 R[75] + 2.22207*10^-6 R[83] + 0.0014592 R[86] - 13.0843 R[88] - 0.00997345 R[89] - 0.25 R[90] - 1166.98 R[91] - 549.449 R[92] - 315.672 R[93] - 645.675 R[94] - 0.25 R[95] - 417.465 R[98] - 1166.97 R[99] + 5.55112*10^-17 R[101] - 1070.52 R[102] - 549.449 R[103] - 1521.45 R[104] - 553.925 R[105] - 243.2 R[106] - 776.85 R[107] - 79.25 R[108] - 315.675 R[109] - 645.936 R[110] - 1749.77 R[111] - 587.1 R[112] - 1305.65 R[113] - 473.2 R[114] - 2913.67 R[115] - 1203.93 R[116] - 1147.2 R[117] - 445.55 R[118] - 61.25 R[121] - 424.437 R[122] - 80.5 R[123] - 1169.33 R[124] + 0.3 R[125] - 3874.9243.199 R[135] - 784.492 R[136] - 1.08333 R[137] - 80.3333 R[138] - 316.569 R[139] - 645.936 R[140] - 1754.82 R[141] - 587.433 R[142] - 1314.58 R[143] + 0.333333 R[144] - 251.15 R[145] - 0.5 R[148] - 0.5 R[149] - 0.5 R[150] - 0.5 R[151] - 473.575 R[152] - 0.5 R[153] - 0.499997 R[154] + R[156] - 18. R[158] - 2917.55 R[159] - 757370. R[160] - 1207.81 R[161] - 1151.15 R[166] - 61.5007 R[169],

R[81] = 0.5 R[75] + 0.25 R[130] + 0.5 R[148] + R[157],

R[82] = R[18] + R[163],

R[84] = R[19] + R[165],

R[85] = - 1.81899*10^-12 R[19] - 0.0223291 R[47] + 0.0000120816 R[48] - 0.5 R[70] - 4.44413*10^-6 R[83] - 0.0029184 R[86] + 0.0000611484 R[88] + 0.0198818 R[89] + 0.5 R[90] + 2333.95 R[91] + 1098.9 R[92] + 631.35 R[93] + 1291.35 R[94] + 834.93 R[98] + 2333.95 R[99] + 2141.05 R[102] + 1098.9 R[103] + 3042.9 R[104] + 1107.85 R[105] + 486.4 R[106] + 1553.7 R[107] + 158.5 R[108] + 631.35 R[109] + 1291.35 R[110] + 3499.55 R[111] + 1174.2 R[112] + 2611.3 R[113] + 946.4 R[114] + 5827.35 R[115] + 2407.85 R[116] + 2294.4 R[117] + 891.1 R[118] + 122.5 R[121] + 834.928 R[122] + 161. R[123] + 2333.95 R[124] + 7749.96 R[127] + 2141.05 R[128] + 3042.9 R[132] + 1107.85 R[133] + 486.4 R[135] + 1553.7 R[136] - 9.99632*10^-7 R[137] + 158.5 R[138] + 631.35 R[139] + 1291.35 R[140] + 3499.56 R[141] + 1174.2 R[142] + 2611.3 R[143] + 502.3 R[145] + 946.4 R[152] + R[153] + 0.999995 R[154] + 5827.32 R[159] + 1.51474*10^6 R[160] + 2407.86 R[161] + 2294.4 R[166] + R[168] + 122.499 R[169],

R[87] = 0.912394 R[47] + 0.141755 R[88] + 4.26154*10^-6 R[89] + 0.912394 R[122] + 0.391778 R[132] + 0.178453 R[133] + 0.141755 R[137] + 0.141755 R[138] + 0.0147369 R[141] + 1.16564 R[143],

R[120] = - 50. R[18] - 19375. R[19] - 0.5 R[45] - 2686.68 R[47] - 64603. R[48] - R[75] + 0.0000200032 R[83] + 0.0131343 R[86] - 30.2525 R[88] - 0.0895506 R[89] - 0.75 R[90] - 10502.8 R[91] - 4945.05 R[92] - 2840.92 R[93] - 5811.08 R[94] - 0.75 R[95] - 3757.24 R[98] - 10503.1 R[99] - 0.5 R[100] - 0.5 R[101] - 67443.1 R[102] - 4945.22 R[103] - 13693.1 R[104] - 4985.33 R[105] - 2188.8 R[106] - 6991.65 R[107] - 713.25 R[108] - 2840.93 R[109] - 5811.6 R[110] - 15748. R[111] - 5283.9 R[112] - 11750.8 R[113] - 4258.8 R[114] - 26223.1 R[115] - 10835.3 R[116] - 10324.8 R[117] - 4009.95 R[118] - 1. R[119] - 551.25 R[121] - 3774.98 R[122] - 724.5 R[123] - 10509.8 R[124] + 0.7 R[125] - 30999.9 R[127] - 67479.2 R[128] - 13700.7 R[132] - 4988.79 R[133] - 2189.3 R[135] - 7011.05 R[136] - 2.74998 R[137] - 716. R[138] - 2842.71 R[139] - 5811.6 R[140] - 15762.2 R[141] - 5284.57 R[142] - 11773.5 R[143] - 2260.35 R[145] + 5.55112*10^-17 R[147] - R[148] - R[149] - R[150] - R[151] - 4259.86 R[152] - 4. R[153] - 3.99998 R[154] - 50.0001 R[158] - 26233.8 R[159] - 6.81633*10^6 R[160] - 10846.3 R[161] - 10332.9 R[166] - 552.255 R[169],

R[126] = 3. R[2] + 3. R[18] + 3. R[19] - 3. R[83] + 3. R[162] + 3. R[163] + 3. R[165],

R[146] = R[45] + 2.05676*10^-6 R[47] + 1.1595*10^-7 R[48] + 8.81823*10^-9 R[88] + 9.07949*10^-9 R[110] + 5.67597*10^-8 R[122] + 2.*10^-6 R[124] + 1.15964*10^-7 R[128] + 2.43722*10^-8 R[132] + 1.11013*10^-8 R[133] + 6.22083*10^-8 R[136] + 8.81836*10^-9 R[137] + 8.81846*10^-9 R[138] + 3.11043*10^-8 R[139] + 9.07949*10^-9 R[140] + 4.18677*10^-6 R[141] + 1.1587*10^-8 R[142] + 7.25124*10^-8 R[143] - R[147] - R[148] - R[149] - R[150] - R[151] + 3.21266*10^-7 R[152] + 3.28951*10^-6 R[159] + 3.30122*10^-6 R[161] + 1.2786*10^-7 R[166] + 2.1241*10^-7 R[169],

R[164] = - 0.9 R[18] - 3.88804 R[47] - 7.94444 R[48] - 0.025 R[75] - R[83] + 0.00007296 R[86] - 0.654215 R[88] - 0.000498673 R[89] - 0.0125 R[90] - 58.3488 R[91] - 27.4725 R[92] - 15.7836 R[93] - 32.2838 R[94] - 0.0125 R[95] - 20.8732 R[98] - 58.3486 R[99] - 53.5262 R[102] - 27.4725 R[103] - 76.0725 R[104] - 27.6962 R[105] - 12.16 R[106] - 38.8425 R[107] - 3.9625 R[108] - 15.7837 R[109] - 32.2968 R[110] - 87.4888 R[111] - 29.355 R[112] - 65.2825 R[113] - 23.66 R[114] - 145.684 R[115] - 60.1963 R[116] - 57.36 R[117] - 22.2775 R[118] - 3.0625 R[121] - 21.2218 R[122] - 4.025 R[123] - 58.4665 R[124] + 0.015 R[125] - 193.749 R[127] - 54.2385 R[128] - 76.2222 R[132] - 27.7644 R[133] - 12.1599 R[135] - 39.2246 R[136] - 0.0541663 R[137] - 4.01667 R[138] - 15.8284 R[139] - 32.2968 R[140] - 87.741 R[141] - 29.3716 R[142] - 65.7289 R[143] + 0.333333 R[144] - 12.5575 R[145] + 3.46945*10^-18 R[147] - 0.025 R[148] - 0.025 R[149] - 0.025 R[150] - 0.025 R[151] - 23.6788 R[152] - 0.025 R[153] - 0.0249999 R[154] - 0.900002 R[158] - 145.877 R[159] - 37868.5 R[160] - 60.3907 R[161] - 57.5577 R[166] - 3.07503 R[169]}

**Appendix C: Basic Pathways Expansion of General Pathway**

Given a general 169-dimensional pathway vector ***R****=[R(j)]*, the issue is how to find an 89-dimensional weight vector ***C_BP_****=[c(j)]* satisfying ***R_cell_****=****WC_BP_***. To that end, the singleton structure of the Basic Pathways matrix ***W*** is summarized.

**Singleton Structure of *W***: The 169×89-dimensional matrix ***W*** = *[W(i,j)]* has 88 singleton rows with indices{ 2, 6, 17, 18, 19, 41, 43, 44, 46, 47, 48, 56, 57, 62, 63, 65, 66, 67, 68, 69, 70, 75, 83, 86, 88, 89, 91, 93, 94, 95, 96, 98, 100, 101, 102, 103, 104, 105, 109, 111, 112, 113, 114, 115, 116, 117, 118, 119, 121, 122, 123, 124, 127, 128, 130, 132, 133, 134, 136, 137, 139, 140, 141, 142, 143, 144, 145, 147, 148, 149, 150, 151, 152, 153, 154, 155, 156, 157, 158, 159, 160, 161, 163, 165, 166, 167, 168, 169}. The 89 columns of ***W*** are the Basic Pathway vectors, 74 of which are singleton, that is, contain at least one non-zero entry from a singleton row. The indices of the singleton columns of ***W*** and the indices of their singleton entries (in brackets {}) are 2 {2}, 6 {6}, 8 {95}, 9 {158}, 10 {134}, 11 {153}, 12 {127}, 13 {150}, 14 {130}, 15 {148}, 17 {17, 144}, 18 {18}, 19 {19}, 20 {167}, 21 {91}, 23 {93}, 24 {94}, 26 {98}, 28 {102}, 29 {104}, 30 {105}, 34 {111}, 35 {112}, 36 {113}, 37 {114}, 38 {115}, 39 {116}, 40 {117}, 41 {41, 118}, 42 {121}, 43 {43, 123}, 44 {44, 145}, 46 {46, 160}, 47 {47}, 48 {48}, 50 {103}, 51 {109}, 52 {110}, 53 {122}, 54 {124}, 55 {128}, 56 {56, 132}, 57 {57, 133}, 58 {136}, 60 {139}, 61 {140}, 62 {62, 141}, 63 {63, 142}, 64 {143}, 65 {65, 152}, 66 {66, 159}, 67 {67, 161}, 68 {68, 166}, 69 {69, 169}, 70 {70}, 71 {96}, 72 {101}, 73 {119}, 74 {147}, 75 {75}, 76{151}, 77 {149}, 78{154}, 79 {155}, 80 {156}, 81 {157}, 82 {163}, 83 {83}, 84 {165}, 85 {168}, 86 {86}, 87 {137}, 88 {88}, 89 {89}. Several of the columns of ***W*** have more than one singleton entry, and fifteen of the columns of ***W*** are singleton deficient. The 74 indices of the singleton columns of ***W*** are collected in the vector ***Si****=[Si(j)]* and the 15 indices of the singleton deficient columns of ***W*** are in the vector ***NSi****=[NSi(j)]*. For each index in ***Si***, the row index of the singleton entry to be used in calculating the weights *c(j)* are collected in the vector ***Sir****=[Sir(j)]*. The 74 entries of ***Sir*** are 2, 6, 95, 158, 134, 153, 127, 150, 130, 148, 144, 18, 19, 167, 91, 93, 94, 98, 102, 104, 105, 111, 112, 113, 114, 115, 116, 117, 118,121, 123, 145, 160, 47, 48, 103, 109, 110, 122, 124, 128, 132, 133, 136, 139, 140, 141, 142, 143, 152, 159, 161, 166, 169, 70, 96, 101, 119, 147, 75, 151, 149, 154, 155, 156, 157, 163, 83, 165, 168, 86, 137, 88, 89. Of the 89 weights *c(j),* 74 of them are determined by singleton theory giving

*c(Si(j)) = R(Sir(j)/W(Sir(j),Si(j)), j=1,…,74.* [1]

The remaining 15 weights are obtained by solving 15 of the 169 linear equations in

*c(1) = (R(135)-0.018685 R(83) – 3.0×10^(-6) R(86))/1.5386,*

*c(3) = R(129) – 2 R(75) – 2 R(148),*

*c(4) = (R(125) - (100 R(18) + 38750 R(19) + R(45) + 5283.25716 R(47) + 129021.85048 R(48) + 2R(75) - 0.000040006 R(83) - 0.026269 R(86) + 46.50366 R(88) + 0.1791 R(89) + 1.5 R(90) + 21005.55498 R(91) + 9890.097545 R(92) + 5681.84089 R(93) + 11622.15 R(94) + 1.5 R(95) + 7514.48982 R(98) + 21006.2877 R(99) + R(100) + R(101) + 134886.15 R(102) + 9890.432767 R(103) + 27386.1 R(104) + 9970.65 R(105) + 4377.6 R(106) + 13983.30125 R(107) + 1426.5 R(108) + 5681.85 R(109) + 11623.19335 R(110) + 31495.95 R(111) + 10567.8 R(112) + 23501.7 R(113) + 8517.6 R(114) + 52446.15 R(115) + 21670.65 R(116) + 20649.6 R(117) + 8019.9 R(118) + 2 R(119) + 1102.49995 R(121) + 7549.96436 R(122) + 1449.0 R(123) + 21019.53233 R(124) + 61999.7088 R(127) + 134958.47172 R(128) + 27401.3012 R(132) + 9977.57384 R(133) + 4378.59791 R(135) + 14022.09925 R(136) + 5.49997 R(137) + 1432.0 R(138) + 5685.42408 R(139) + 11623.19335 R(140) + 31524.33015 R(141) + 10569.13141 R(142) + 23547.013648 R(143) + 4520.7 R(145) + 2 R(148) + 2 R(149) + 2 R(150) + 2 R(151) + 8519.71702 R(152) + 8 R(153) + 8 R(154) + 100.0 R(158) + 52467.67487 R(159) + 1.36327×10^7^ R(160) + 21692.54069 R(161) + 20665.80821 R(166) + 1104.50866 R(169)))/8.*

*c(5) = R(131)-6.90575R(47)-18.652 R(48)-R(88) – 10^(-6)R(89)-2.92658 R(141),*

*c(7) = (R(90)-15,500 R(19) – 1,669.9 R(47) -4,282.1 R(48) – R(70))/2,*

*c(16) = R(162) – R(83*

*c(22) = R(92) – R(86),*

*c(25) = (R(97)-10 R(18))/0.2,*

*c(27) = R(100),*

*c(31) = R(106) - 0.095125 R(47) - 3.0 ×10^(-6) R(86) - 0.7456 R(99) - 0.345903 R(103) - 0.095125 R(122) - 4.562269 R(124) - 7.988025 R(141) - 0.613084 R(152) - 6.277492 R(159) - 6.29983 R(161) - 1.013376 R(169),*

*c(32) = R(107) - 0.91239 R(47) - 1.864 R(48) - 0.14176 R(88) + 4.26154×10^(-6)R(89) - 0.91239 R(122) - 1.864 R(128) - 0.391778 R(132) - 0.17845 R(133) - 0.14176 R(137) - 0.14176 R(138) - 0.014737 R(141) - 1.16564 R(143) - 0.0479 R(166),*

*c(33) = R(108)-R(88),*

*c(45) = R(45) – R(147) – R(148) – R(149) – R(150) – R(151),*

*c(49) = R(99) – 2.09293 R(141) – 0.16063 R(152 – 1.64476 R(159) – 1.65061 R(161) – 0.10621R(169),*

*c(59) = R(138)- 8.222834 R(89)*
